# The estrogen signaling pathway reprograms prostate cancer cell metabolism and supports proliferation and disease progression

**DOI:** 10.1172/JCI170809

**Published:** 2024-04-16

**Authors:** Camille Lafront, Lucas Germain, Gabriel H. Campolina-Silva, Cindy Weidmann, Line Berthiaume, Hélène Hovington, Hervé Brisson, Cynthia Jobin, Lilianne Frégeau-Proulx, Raul Cotau, Kevin Gonthier, Aurélie Lacouture, Patrick Caron, Claire Ménard, Chantal Atallah, Julie Riopel, Éva Latulippe, Alain Bergeron, Paul Toren, Chantal Guillemette, Martin Pelletier, Yves Fradet, Clémence Belleannée, Frédéric Pouliot, Louis Lacombe, Éric Lévesque, Étienne Audet-Walsh

**Affiliations:** 1 Department of Molecular Medicine, Université Laval, Quebec City, Québec, Canada.; 2 Endocrinology and Nephrology Division, CHU de Québec – Université Laval Research Center (CRCHUQ-UL), Quebec City, Québec, Canada.; 3 Cancer Research Center (CRC) of Université Laval, Quebec City, Québec, Canada.; 4 Department of Obstetrics, Gynecology and Reproduction, Université Laval, Quebec City, Québec, Canada.; 5 Reproduction, Mother and Youth Health Division, CRCHUQ-UL, Quebec City, Québec, Canada.; 6 Department of Medicine, Université Laval, Quebec City, Québec, Canada.; 7 Oncology Research Division, CRCHUQ-UL, Quebec City, Québec, Canada.; 8 Department of Pathology, CHU de Québec-Université Laval, Quebec City, Québec, Canada.; 9 Department of Surgery,; 10 Faculty of Pharmacy, and; 11 Department of Microbiology-Infectious Diseases and Immunology, Université Laval, Quebec City, Québec, Canada.; 12 Infectious and Immune Diseases Research Division, CRCHUQ-UL, Quebec City, Québec, Canada.; 13 ARThrite Research Center, Université Laval, Quebec City, Québec, Canada.

**Keywords:** Endocrinology, Oncology, Glucose metabolism, Prostate cancer, Sex hormones

## Abstract

Just like the androgen receptor (AR), the estrogen receptor α (ERα) is expressed in the prostate and is thought to influence prostate cancer (PCa) biology. Yet the incomplete understanding of ERα functions in PCa hinders our ability to fully comprehend its clinical relevance and restricts the repurposing of estrogen-targeted therapies for the treatment of this disease. Using 2 human PCa tissue microarray cohorts, we first demonstrate that nuclear ERα expression was heterogeneous among patients, being detected in only half of the tumors. Positive nuclear ERα levels were correlated with disease recurrence, progression to metastatic PCa, and patient survival. Using in vitro and in vivo models of the normal prostate and PCa, bulk and single-cell RNA-Seq analyses revealed that estrogens partially mimicked the androgen transcriptional response and activated specific biological pathways linked to proliferation and metabolism. Bioenergetic flux assays and metabolomics confirmed the regulation of cancer metabolism by estrogens, supporting proliferation. Using cancer cell lines and patient-derived organoids, selective estrogen receptor modulators, a pure anti-estrogen, and genetic approaches impaired cancer cell proliferation and growth in an ERα-dependent manner. Overall, our study revealed that, when expressed, ERα functionally reprogrammed PCa metabolism, was associated with disease progression, and could be targeted for therapeutic purposes.

## Abstract 2

ERα is heterogeneously expressed in prostate cancer and functionally reprograms cell metabolism to promote proliferation when present.

## Introduction

Prostate cancer (PCa) is the most common cancer for men in 112 countries (1). This disease is highly dependent on the androgen receptor (AR), a transcription factor that modulates several biological pathways essential for the growth and survival of PCa cells. Notably, AR regulates cancer cell metabolism to synthesize energy, such as promoting glycolysis, mitochondrial respiration, and fatty acid β-oxidation, as well as inducing cancer cell proliferation (2–5). This dependency of PCa cells on AR activity is the reason that hormonal therapies used to treat PCa either target the production of these hormones through androgen deprivation therapies (ADTs), or the AR signaling pathway using anti-androgens (2, 5). Tumor cells initially respond favorably to these treatments but inevitably evolve to the life-threatening form of the disease named castration-resistant PCa (CRPC) (2, 5, 6); thus, there is an urgent need to find new therapeutic targets to treat this lethal disease.

In addition to androgens, estrogens, notably the most potent endogenous estrogen estradiol (E_2_), can also modulate PCa cell biology (as reviewed in refs. 7–9). For example, the combination of both androgens and estrogens is essential for the induction of prostate carcinogenesis in preclinical models (10–13). Moreover, mice with KO of *Cyp19a1*, which encodes the aromatase enzyme essential for estrogen biosynthesis, fail to develop PCa despite exhibiting increased androgen production (14). Mice with aromatase overexpression, which leads to an increase of the estrogens/androgens ratio, do not develop PCa either (15). In addition, plasma E_2_ levels are positively correlated with high-grade PCa (16) and, in patients undergoing ADTs, with evolution to CRPC (17). Consequently, all these data suggest that the estrogen signaling pathway is as important as the androgen pathway for PCa biology.

The effects of estrogens on prostate cells are thought to be mostly mediated by the estrogen receptors ERα and ERβ (8). They are both transcription factors of the nuclear receptor family like the AR, however with opposite effects in the prostate. ERβ is thought to be a tumor suppressor (18–20), whereas ERα is associated with oncogenic functions (10, 21, 22). In vivo models support an oncogenic role for ERα, as its genetic ablation in mouse models blocks the initiation of PCa following treatment with testosterone plus E_2_ (8). Conversely, mice that no longer express ERβ (βER-KO) exhibit increased hyperplasia and androgen signaling (20). Thus, the oncogenic effects of E_2_ in the prostate are likely conducted through the activation of ERα.

Considering these data, ERα represents a potentially effective therapeutic target in PCa. One of the very first ADTs was to give high doses of estrogens to patients, which generated a negative feedback loop in the hypothalamic/pituitary/testicular axis and thus induced a pharmacological castration (2). However, this approach was not intended to directly target ERα’s action in the prostate. To target ERα and the “endogenous” estrogen signaling pathway, as opposed to high exogenous estrogen doses, many drugs are currently available to inhibit the action of this receptor in the context of ERα-positive breast cancer (23), namely selective estrogen receptor modulators (SERMs). Several studies have attempted to evaluate the efficacy of SERMs in different clinical settings, such as treating high-grade prostate intraepithelial neoplasia (HGPIN), to prevent PCa recurrence following surgery, treating treatment-naive bone metastatic PCa, or treating CRPC. However, conflicting results were obtained, with either positive responses (24–26) or no significant changes (27–29). In these studies, no stratification of PCa patients was performed on the basis of the presence or absence of ERα prior to testing for SERMs, which possibly explains such conflicting results. Another limitation of using SERMs to treat PCa is our incomplete understanding of the role of ERα as a transcription factor in the prostate and PCa, given, notably, that the most commonly used PCa cell lines do not express ERα, or express a mutated AR that can be activated by E_2_ (e.g., LNCaP cells) (2, 7).

In this study, our objective was to elucidate the role of estrogens, and particularly of ERα, in the biology of PCa. We first used a clinically validated approach (that is normally used for breast cancer) to determine the expression of ERα in PCa samples. Despite its heterogeneity, the expression of ERα positively correlated with more aggressive prostate tumors and clinical progression. We then used in vivo preclinical mouse models (WT and PCa), human PCa cell lines, and patient-derived organoids (PDOs) to study the cellular effects of modulating the estrogen signaling pathway in PCa. We observed that hundreds of genes were differentially expressed, both in vitro and in vivo, highlighting the fact that the reprogramming of cancer cell metabolism was a major function of ERα in PCa and supported the aberrant proliferation of these cancer cells. Finally, we demonstrated in preclinical models that, when ERα was expressed, SERMs could be used as efficient therapeutic agents against ERα-expressing prostate tumors.

## Results

### ERα expression is heterogeneous in PCa and, when expressed, is associated with a more aggressive disease.

**Figure 1:**
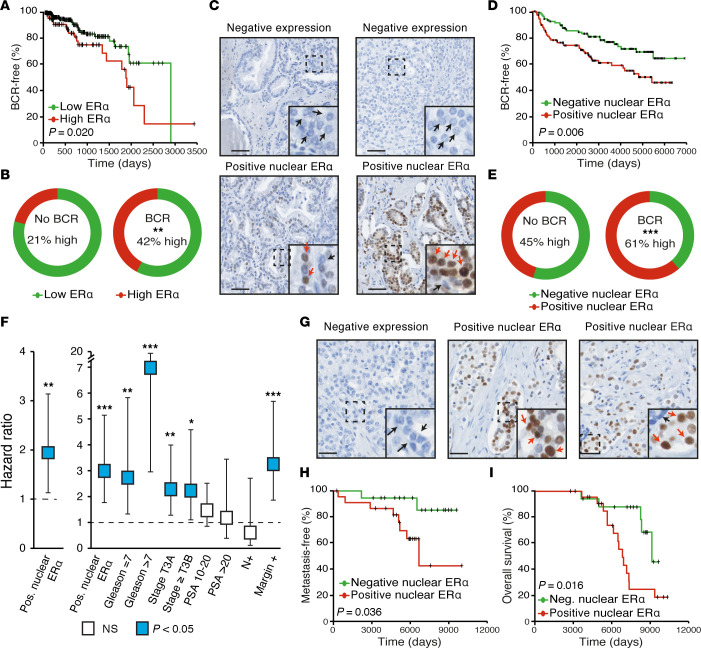
ERα expression is heterogenous in PCa and, when nuclear (active), is associated with BCR. (**A**) Kaplan-Meier of BCR-free survival following radical prostatectomy in patients from TCGA-PRAD cohort with high or low ERα protein expression levels (no distinction between nuclear and cytoplasmic localization). (**B**) Proportions of patients from TCGA cohort with high or low ERα protein expression levels, with and without BCR (**P < 0.0019, χ^2^ test). (**C**–**F**) Analysis of the Belledant et al. (32) cohort. (**C**) Representative images of ERα IHC in 4 patients with PCa. Black and red arrows, respectively, highlight negative and positive staining. Scale bars: 50 μm. Original magnification, ×3.1 (enlarged insets in **C** and **G**). (**D**) Kaplan-Meier BCR-free survival following radical prostatectomy in patients with positive versus negative nuclear levels of ERα. (**E**) Proportions of patients from the TMA cohort with positive or negative nuclear levels of ERα, with and without BCR (***P < 0.001, χ^2^ test). (**F**) Cox regression analyses of the effect of positive (Pos.) nuclear ERα levels on the risk of BCR (*P < 0.05, **P < 0.01, and ***P < 0.001). Boxes illustrate HRs with their respective 95% CIs. Results are shown without (left) and with (right) additional BCR risk factors. Reference groups for covariables: Gleason score of 6; T2c stage and below; presurgery PSA levels under 10 ng/mL; negative lymph node invasion and negative margins. (**G**–**I**) Analysis of an independent cohort of patients who received neoadjuvant hormonotherapy before surgery. (**G**) Representative IHC images of ERα expression in 4 patients with PCa. Black and red arrows, respectively, highlight negative and positive staining. Scale bars: 50 μm. (**H** and **I**) Kaplan-Meier survival analysis in patients with positive versus negative (Neg.) ERα nuclear levels in the development of metastasis (**H**) and overall survival (**I**). For Kaplan-Meier survival curves, the log-rank test *P* value is shown in the inset.

We first studied ERα total protein levels by reanalyzing proteomics data from The Cancer Genome Atlas (TCGA) consortium (the prostate adenocarcinoma [PRAD] data set) (30, 31), with protein levels separated according to low versus high expression levels. We found that high ERα protein levels were associated with a shorter biochemical recurrence–free (BCR-free) survival rate, the first indication of PCa progression following surgery (Figure 1A). In patients with BCR, 42% had high ERα protein levels compared with 21% of patients without BCR (Figure 1B; *P* = 0.002). Despite associating ERα total protein levels with BCR, proteomics analyses did not distinguish between ERα levels in the different cells from the tumor microenvironment, nor did the analyses distinguish between active (nuclear) or inactive (cytoplasmic) receptors.

Consequently, we then performed an IHC study of ERα in human PCa samples, similar to what is routinely performed for breast cancer. Indeed, in the breast cancer field, the expression pattern of ERα is evaluated before prescribing (or not) hormonal therapies. To determine whether such a clinical trajectory could be translated to PCa, we then investigated the expression profiles of ERα in prostate tumors using the clinical pipeline for defining ERα expression status in patients with breast cancer at our local hospital, using a clinically validated antibody for this receptor (clone EP1, Dako). The specificity of the ERα antibody was further confirmed using the established breast cancer cell lines MCF7 (ERα-positive) and MDA-MB-231 (ERα-negative) (Supplemental Figure 1A; supplemental material available online with this article; https://doi.org/10.1172/JCI170809DS1). We then assessed ERα expression levels in an established prostate tissue microarray (TMA) comprising tissues from 239 patients (see Supplemental Table 1 for the cohort description) (32, 33).

First, expression of ERα in human PCa was highly heterogeneous between tumors, being either absent or present in nuclei, cytoplasm, and/or stroma (Figure 1C and Supplemental Figure 1, B–E). ERα staining was stronger in stromal cells, as reported previously (34–36), and was high in 70% of the samples (Supplemental Figure 1F). Less studied in cancer cells due to lower expression, positive nuclear ERα staining in cancer cells, indicative of an activated receptor, was detected in 51% of patients’ tumors (Supplemental Figure 1F). Following radical prostatectomy, nuclear ERα positivity was associated with a shorter BCR-free survival rate (log-rank *P* value of 0.006; Figure 1D). Indeed, 61% of patients with BCR had positive ERα nuclear expression compared with 45% of patients without BCR (Figure 1E; *P* < 0.001). In univariate Cox regression analyses, positive ERα nuclear levels were associated with a HR of 1.94-fold higher risk of BCR following surgery compared with negative ERα nuclear levels (Figure 1F; left). Importantly, this association between nuclear ERα (active) status and BCR remained significant when the model was adjusted for other variables associated with BCR in multivariate analyses, such as the Gleason score, tumor stage, prostate-specific antigen (PSA) levels at diagnosis, nodal invasion status, and surgical margins (HR for positive nuclear ERα: 3.02; Figure 1F; right). On the contrary, cytoplasmic and stromal positivity for ERα was not significantly associated with a BCR-free survival rate (Supplemental Figure 1, G and H).

Next, we validated these results in an independent data set comprising data on 41 patients who received neoadjuvant ADTs before surgery (with 32 patients of 41 who received both ADT and anti-androgens; see cohort description in Supplemental Table 2). Consequently, even though these patients did not have a “clinical CRPC” at surgery, the samples studied were composed of cancer cells that survived castration and were evolving to lethal CRPC. In this cohort, ERα was quantified using the same pipeline and threshold established for the discovery cohort, again by reviewers blinded to the clinical data. In this setting, nuclear ERα protein detection was positive in 54% of the samples (22 of 41; Figure 1G and Supplemental Figure 1, I and J). In this data set, which is representative of more aggressive tumors, most patients experienced BCR (>60%). Importantly, positive nuclear ERα expression was significantly associated with a faster time to metastasis and decreased overall patient survival (Figure 1, H and I; multivariate analyses using Cox regressions were not performed due to the lack of statistical power). This cohort allowed us to link nuclear ERα expression in cancer cells with the evolution to lethal CRPC.

As seen in the discovery cohort, stromal ERα levels were much higher than in the epithelial/tumoral compartment but, again, were not associated with disease progression in the survival analyses (Supplemental Figure 1, K and L). These results, even though stromal ERα is most probably important in PCa biology (see Supplemental Discussion), led us to focus our investigation on the functional role of ERα specifically in cancer cells and the epithelial compartment.

Overall, using a clinically validated ERα antibody in 2 TMAs, these results first indicated that ERα expression is heterogeneous between patients and that it is not expressed in all tumors. Consequently, if a patient is given any ERα-targeted therapy, its expression in cancer cells should first be validated. Secondly, when expressed, often only a low percentage of cells are positive for ERα (>1%–10%). Yet, positive nuclear (active) ERα levels were significantly associated with PCa progression following prostatectomy, and even so in tumors from patients treated with neoadjuvant ADTs in relation to metastases and overall survival. Together, these results confirm that ERα can be expressed in human prostate tumors and suggest that ERα-positive or ERα-negative status may apply to PCa tumors and be pertinent for prognosis and repurposing of anti-estrogen therapies.

### Modulation of the normal mouse prostate transcriptome in vivo by androgens and estrogens.

**Figure 2:**
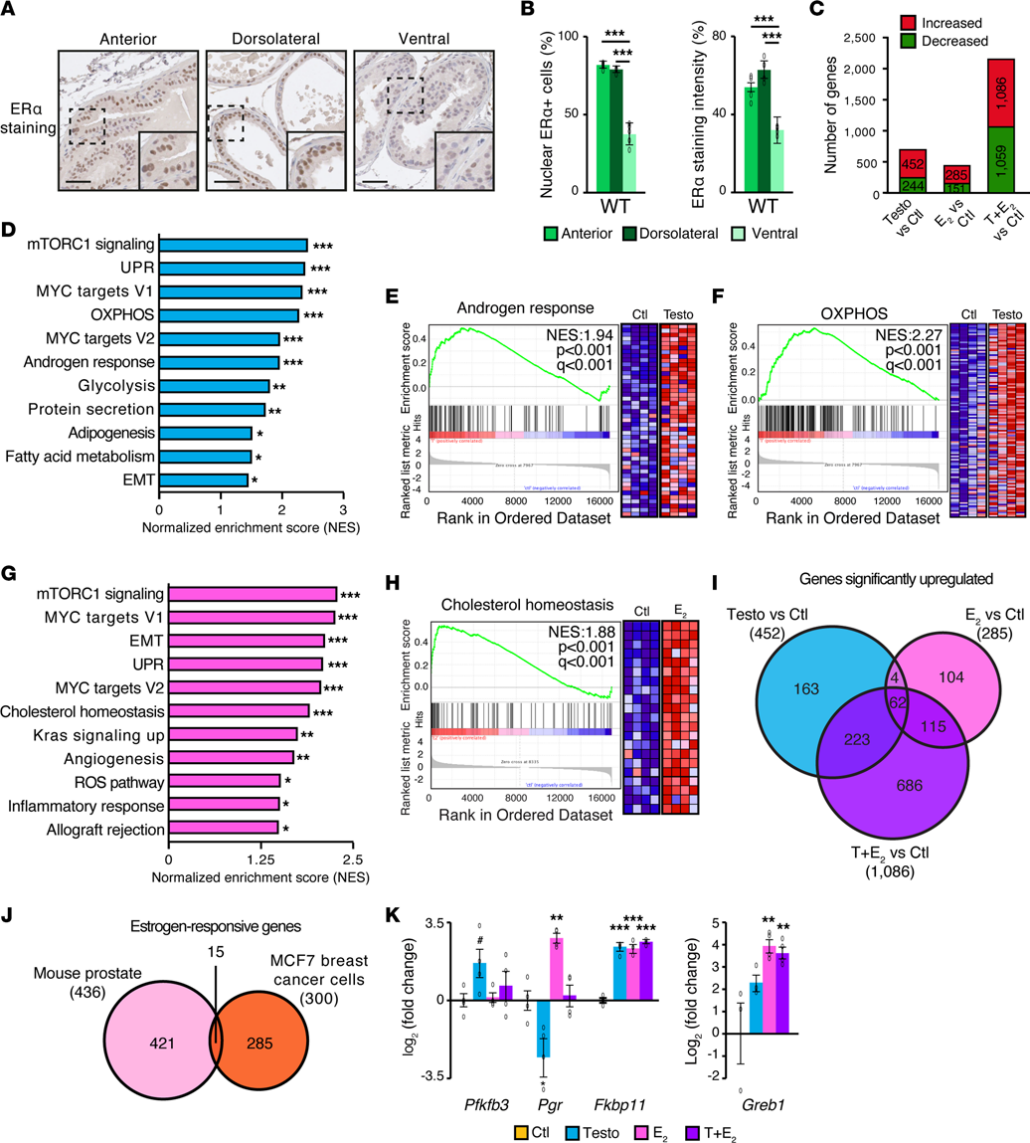
Estrogens modulate the normal prostate transcriptome in vivo, activating oncogenic pathways similar to those activated with androgen stimulation. (**A**) Representative IHC images of ERα in normal mouse prostate lobes. Scale bars: 50 μm. Original magnification, ×1.68 (enlarged insets). (**B**) Quantification of ERα-positive staining and ERα staining intensity in normal mouse prostate lobes (*n* = ~2,700 cells/animal, *n* = 5 animals/lobe). (**C**–**I**) RNA-Seq analyses of the murine prostate transcriptome 24 hours after injections with vehicle (Ctl), testosterone (Testo), E_2_, or both (T+E_2_). Mice were castrated 3 days before injections to ensure hormonal deprivation. (**C**) Number of significantly differentially expressed genes (DEGs) following pairwise comparisons between conditions. The thresholds used were a fold change of 1.75 or more or –1.75 or less and a *P* value with a FDR of less than 5%. (**D**) GSEA normalized enrichment score (NES) following treatment with testosterone. (**E** and **F**) GSEA diagrams and heatmaps for the androgen response (**E**) and the OXPHOS (**F**) gene sets following testosterone treatment in vivo. (**G**) GSEA NES for enrichment following E_2_ treatment in vivo. (**H**) GSEA diagram and heatmap for the cholesterol homeostasis gene set following E_2_ treatment. For **E**, **F** and **H**, NESs, *P* values, and *q* values are indicated on each diagram, and only core genes of each pathway are shown. *q < 0.05, **q < 0.01, and ***q < 0.001 in GSEA (**D** and **G**). (**I**) Venn diagram of upregulated genes for each pairwise comparison. (**J**) Venn diagram of estrogen-responsive genes in breast cancer cells (MCF7), using the data set from (41), and in the mouse prostate. Circle and overlap sizes are not proportional to the number of genes. (**K**) qRT-PCR analysis of positive controls for androgenic (*Pfkfb3* and *Fkbp11*) and estrogenic regulation (*Pgr*, *Fkbp11*, and *Greb1*). For **B** and **K**, results are shown as the average with SEM (*n =* 4 mice/treatment); ^#^*P* < 0.10; **P < 0.01 and ***P < 0.001, by 1-way ANOVA.

To gain preliminary insights into the influence of ERα on PCa biology, we first sought to determine the ERα transcriptome in the normal prostate. Mouse studies showed that ERα-positive cells were widely distributed throughout the prostate epithelium, albeit at higher percentages in the anterior and dorsolateral prostate lobes (>75% ERα-positive cells) than in the ventral prostate (37% ERα-positive cells) (Figure 2, A and B). Staining intensity was also studied as an indirect indicator of the relative amount of nuclear ERα positivity per epithelial cell and showed a similar pattern between the lobes (>60% intensity in both the anterior and dorsolateral lobes, versus ~30% intensity in the ventral prostate). Irrespective of the prostate lobe, ERα staining was mostly nuclear.

Since androgens can be converted into estrogens by the aromatase enzyme, it is reasonable to investigate the estrogen signature in parallel with androgens’ effects. To this end, mice were first castrated to inhibit both androgen and estrogen production by the testes. After 72 hours to ensure steroid deprivation, animals were then treated for 24 hours with the vehicle, testosterone, E_2_, or both hormones to study the androgen and estrogen transcriptional signatures in vivo in the normal prostate. In this short-term setting (similar to the settings defined by Pihlajamaa et al., to study the androgen response) (37), the prostate weight was not altered 4 days after castration (Supplemental Figure 2A), as opposed to the long-term impact of castration that normally leads to a greater than 90% decrease in prostate weight (38). Given that the estrogen transcriptional response was, to the best of our knowledge, never defined in the normal prostate or in PCa, we then performed RNA-Seq analyses using this experimental design. First, in the WT mouse prostate, treatment with testosterone was found to alter the expression of 696 genes (Figure 2C). In parallel, E_2_ led to the significant modulation of 436 genes (Figure 2C). Interestingly, activation of both pathways simultaneously yielded the greatest transcriptional response, with 1,086 and 1,059 genes up- and downregulated, respectively (Figure 2C). All genes significantly modulated by each treatment are listed in Supplemental Table 3.

To identify the biological pathways regulated by androgens, estrogens, or both, we performed gene set enrichment analysis (GSEA) (Figure 2, D–H, and Supplemental Figure 2, B–D). As expected, activation of the AR by testosterone induced a transcriptional response linked to the androgen response, as well as activation of key oncogenic pathways in PCa (4, 39), including the mTORC1 and MYC signaling pathways (Figure 2, D and E, and Supplemental Figure 2B). Testosterone also upregulated pathways linked to cell metabolism in the normal prostate, inducing genes associated with oxidative phosphorylation (OXPHOS) and glycolysis, the 2 major pathways leading to ATP synthesis (Figure 2, D and F, and Supplemental Figure 2C). In addition, we observed an enrichment of pathways linked to lipid metabolism, such as fatty acid metabolism and adipogenesis pathways (Figure 2D), as reported previously in the mouse prostate (37). Overall, AR activation in the normal prostate induced pathways associated with proliferation and metabolism.

Interestingly, treatment with E_2_ induced a transcriptional signature generally similar to the androgen-dependent signature, with notable upregulation of genes linked to protein synthesis and cellular proliferation such as the mTORC1 and MYC signaling pathways (Figure 2G and Supplemental Figure 2D). E_2_ also induced specific pathways not targeted by androgens in the prostate, such as the cholesterol homeostasis signature, KRAS activation, and pathways related to immunity and angiogenesis (Figure 2, G and H). Even though testosterone could be aromatized into E_2_, the small overlap between genes regulated by these 2 individual treatments suggests that minimal aromatization, if any, occurred during the 24-hour treatment time frame of the current study (Figure 2I). Indeed, the circulating hormone levels in mice 24 hours after injection of testosterone, E_2_, or both clearly showed specific hormonal exposure (Supplemental Figure 2E).

The combination of both hormones further increased total transcriptional regulation (Figure 2, C and I), but most of these modulated genes were part of the same biological pathways already upregulated by individual treatments, such as the mTORC1 and MYC signaling pathways and cell metabolism pathway genes (Supplemental Figure 2, F and G). Quantitative real-time reverse transcription PCR (qRT-PCR) confirmed the enrichment of metabolic genes following all 3 hormonal combinations (Supplemental Figure 2H).

Of note, the GSEA early and late estrogen response gene signatures, established using mostly breast cancer models (40), were not significantly modulated by E_2_ in the normal prostate. As such, these results suggest that the transcriptional response modulated by estrogens was distinct between the normal prostate and the classic “estrogen response” transcriptional signatures. To confirm this supposition, we compared the top 300 identified estrogen-responsive genes in the MCF7 breast cancer cell model (41) with the estrogen-responsive genes identified here in the mouse prostate and observed little overlap, with only 15 of 300 genes (5%) common to both lists (Figure 2J). In these 15 genes, we identified well-known ERα target genes, such as *Greb1* and *Pgr*, as also being positively regulated by estrogens in the prostate (Figure 2K). Comparison with a second estrogen-treated MCF7 data set (42) also indicated very few genes shared with the mouse prostate’s estrogen response (Supplemental Figure 2I).

Overall, these results show that, in the normal mouse prostate, E_2_ stimulation leads to a distinct transcriptional signature from the “classic” estrogen response that partially mimics androgen stimulation by promoting biological pathways linked to cell proliferation and metabolism.

### Reprogramming of the mouse PCa transcriptome in vivo by androgens and estrogens.

**Figure 3:**
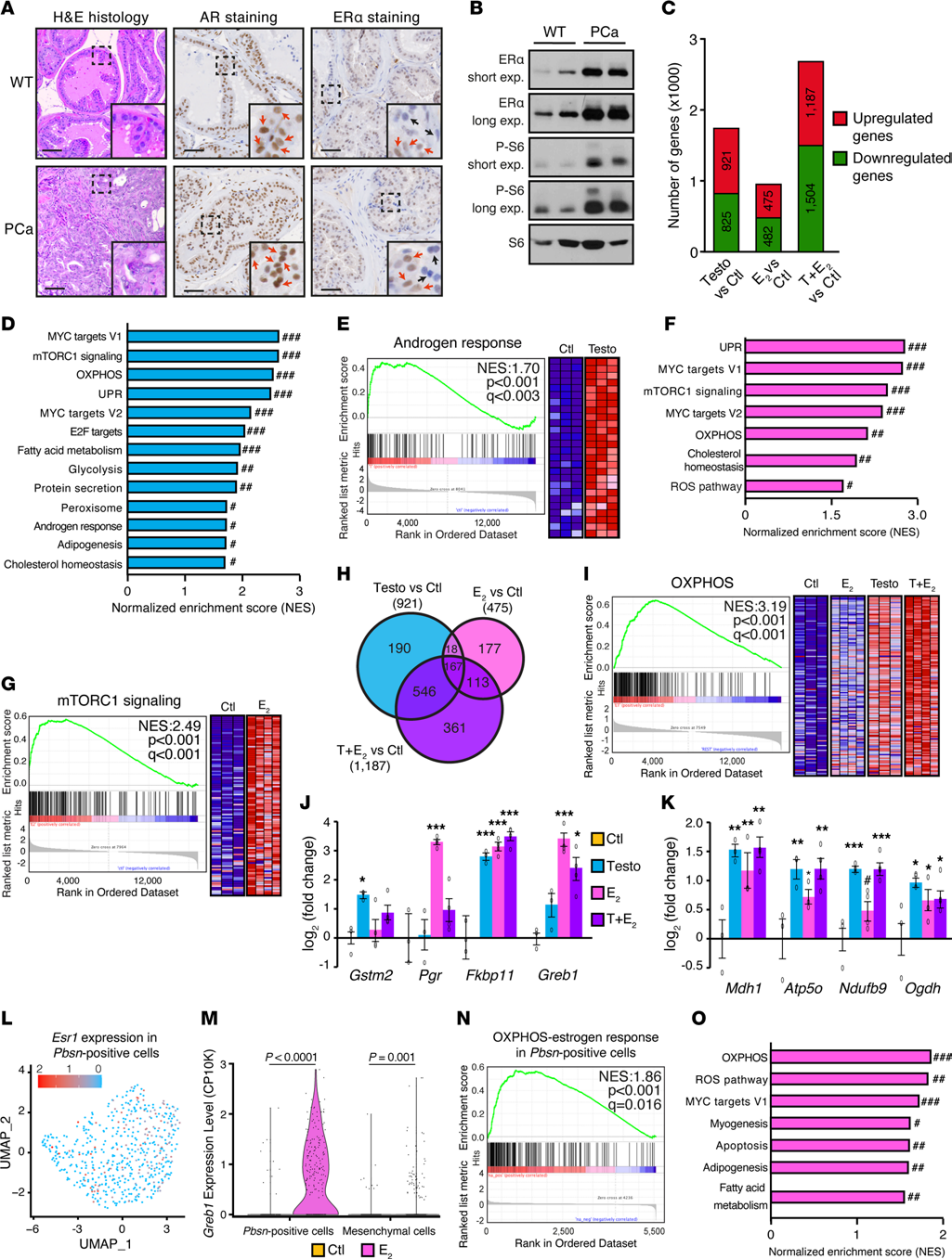
Estrogens activate oncogenic pathways in a PCa mouse model. (**A**) Representative of H&E staining and staining for AR and ERα in prostates from 24-week-old WT and PCa-developing mice. Black and red arrows, respectively, highlight negative and positive staining. Scale bars: 50 μm. Original magnification, ×3.1 (enlarged insets). (**B**) Western blot of prostate samples from WT and PCa-developing mice. Phosphorylated S6 (p-S6) shows activation of the mTOR signaling following prostate-specific deletion of *Pten* in tumors. S6 was used as the loading control. exp., exposure. (**C**–**I**) RNA-Seq analyses of mouse PCa tumors following a 24-hour treatment in vivo with vehicle, testosterone, E_2_, or both. Mice were castrated 3 days before injections to ensure steroid deprivation. (**C**) Number of DEGs following pairwise comparisons. (**D** and **F**) NES of GSEA following treatment with testosterone (**D**) or E_2_ (**F**). ^#^*q* < 0.05, ^##^*q* < 0.01, and ^###^*q* < 0.001. (**E**, **G**, and **I**) GSEA diagrams and heatmaps for the androgen response following testosterone treatment (**E**), the mTORC1 gene set following E_2_ treatment (**G**), and the OXPHOS gene set following testosterone plus E_2_ treatment (**I**). Only core genes are shown. (**H**) Venn diagram of upregulated genes for each pairwise comparison. (**J** and **K**) qRT-PCR analysis of positive controls (**J**) and metabolic genes (**K**) following treatments. Results are shown as the mean ± SEM (3–4 mice/condition). (**L**–**O**) Single-cell RNA-Seq analyses from tumoral murine prostates, with and without treatment with E_2_ (*n* = 2 mice/condition). (**L**) *Esr1* expression in *Pbsn*-positive epithelial cells (in log scale of [counts/10K (CP10K) + 1]). (**M**) *Greb1* expression in mesenchymal and epithelial *Pbsn*–positive clusters. (**N** and **O**) NES of GSEA analysis enriched following E_2_ treatment in *Pbsn*-positive epithelial cells (**O**), with the GSEA diagram for the OXPHOS gene set (**N**). *P < 0.05, **P < 0.01, and ***P < 0.001, by 1-way ANOVA (**J** and **K**) or 2-tailed Student’s *t* test (**M**).

After defining the estrogen transcriptional response in the normal prostate, we then studied this hormonal response in an established transgenic mouse model that develops PCa (C57BL/6J PB-Cre4*^+/–^* *Pten^fl/fl^*) (Figure 3A; left) (43). Most tumor cells had strong nuclear AR expression (Figure 3A middle, and Supplemental Figure 3A). As observed in human samples (Figure 1), nuclear ERα expression was heterogenous in mouse tumors (Figure 3A, right, and Supplemental Figure 3A). Compared with the normal prostate, the number of nuclear ERα–positive cells in murine tumors remained mostly the same, with only a slight increase in the dorsolateral lobes (Supplemental Figure 3B). However, given the increased cellularity within the tumors, total ERα levels were higher, as shown by Western blot analyses (Figure 3B).

We next performed bulk RNA-Seq experiments in this PCa mouse model using a methodology similar to one previously described (37). Testosterone treatment modulated the expression of 1,746 genes (Figure 3C); that is, 2-fold more genes were expressed following testosterone treatment than in the normal prostate (Figure 2C). In the case of E_2_, a total of 957 genes were significantly modulated (Figure 3C), which again was a 2-fold greater number than in the normal prostate (Figure 2C) and correlated with increased ERα expression in prostate tumors. Hormone cotreatment induced the greatest transcriptional response, with the modulation of a total of 2,691 genes (Figure 3C). All genes that were significantly modulated by each treatment are listed in Supplemental Table 4.

Second, we conducted GSEA analyses to highlight the biological pathways regulated by androgens and estrogens. Activation of the AR in PCa induced gene signatures similar to those seen in the normal prostate, such as the androgen response, MYC targets, mTORC1 signaling, OXPHOS, and fatty acid metabolism (Figure 3, D and E, and Supplemental Figure 3, C–E). Some new gene signatures specifically regulated in mouse PCa were observed, such as those for cholesterol homeostasis.

Like androgens, we found that multiple oncogenic pathways were induced by estrogens, such as pathways for mTORC1 signaling, MYC targets, cholesterol homeostasis, and ROS (Figure 3, F–H, and Supplemental Figure 3, C–E), which were also similarly induced in the normal prostate (Figure 2, G and H). Notably, the OXPHOS pathway was upregulated (Figure 3I), but was not found to be enriched by estrogens in the normal prostate (Figure 2G).

Finally, as observed in the normal mouse prostate, the combination of testosterone and E_2_ led to a stronger transcriptional response (Figure 3, C and H), while stimulating mostly the same gene signatures, such as those for OXPHOS, MYC targets, mTORC1 signaling, and fatty acid metabolism, as individual treatments did (Supplemental Figure 3, C–F). Altogether, these results indicate that both androgens and estrogens had a major effect on the mouse PCa transcriptome in vivo.

Furthermore, E_2_ treatment strongly induced expression of the well-known ERα target genes *Greb1* and *Pgr* (Figure 3J), as well as of metabolic genes (Figure 3K). Most of the estrogenic response in this mouse PCa model was distinct from the classic estrogen response, with less than 11% overlap with the MCF7 estrogen response (Supplemental Figure 3, G and H). Clearly, the estrogen transcriptome was distinct in breast cancer compared with that of the prostate and PCa; yet, the prostate-specific estrogen signature showed an important intersection between the mouse prostate and PCa tissues, with an overlap of 63% of estrogen-responsive genes (Supplemental Figure 3I).

Given that the prostate has complex cell populations (38), we next wanted to better identify the estrogenic signature in the epithelial/tumoral component using the prostate-specific *Pten*-KO model. To this end, we performed single-cell RNA-Seq in PCa-developing mice with and without 24-hour treatment with E_2_. As expected, a substantial diversity of cell types was detected, including various epithelial cell populations, mesenchymal/stromal cell subgroups, and immune cell types (Supplemental Figure 4A). These cell subtypes were identified with specific markers described by Karthaus and colleagues (38) and included *Epcam* and *Krt8* for the epithelial, *Krt5* for the basal, and *Col5a2* and *Rspo3* for the mesenchymal/stromal compartments (Supplemental Figure 4, B–F). *Esr1*, which encodes ERα, was detected in mesenchymal (stromal) cells (Supplemental Figure 4G), consistent with high protein levels in the stroma (Figure 1 and Supplemental Figure 1). Importantly, *Esr1* was also expressed in epithelial cells expressing epithelial luminal markers, such as *Pbsn* and *Krt8* (Figure 3L, and Supplemental Figure 4, D, G, and H). These *Pbsn*-positive cells (Supplemental Figure 4H), corresponding to both luminal cells actively secreting prostatic fluid as well as to cells in the tumoral compartment with directed *Pten* deletion in this PCa mouse model, exhibited the modulation of 138 genes following E_2_ stimulation, notably the induction of *Greb1* expression (Figure 3M and Supplemental Figure 4, I and J). Of note, *Esr2*, which encodes ERβ, was undetectable in almost all cell types analyzed (Supplemental Figure 4K). Next, we performed GSEA analyses to study the estrogenic response in these *Pbsn*-positive cells, highlighting OXPHOS as the major pathway enriched following E_2_ treatment (Figure 3, N and O), as well as other pathways promoting proliferation like those for MYC targets and fatty acid metabolism (Figure 3O, and Supplemental Figure 4L). Altogether, these results confirm that *Esr1* (ERα) was expressed in both the stromal and epithelial/tumor components of the prostate, and that, importantly, estrogens induced a metabolic gene signature in the epithelial/tumor compartment.

### Functional reprogramming of human PCa cell metabolism by estrogens.

**Figure 4:**
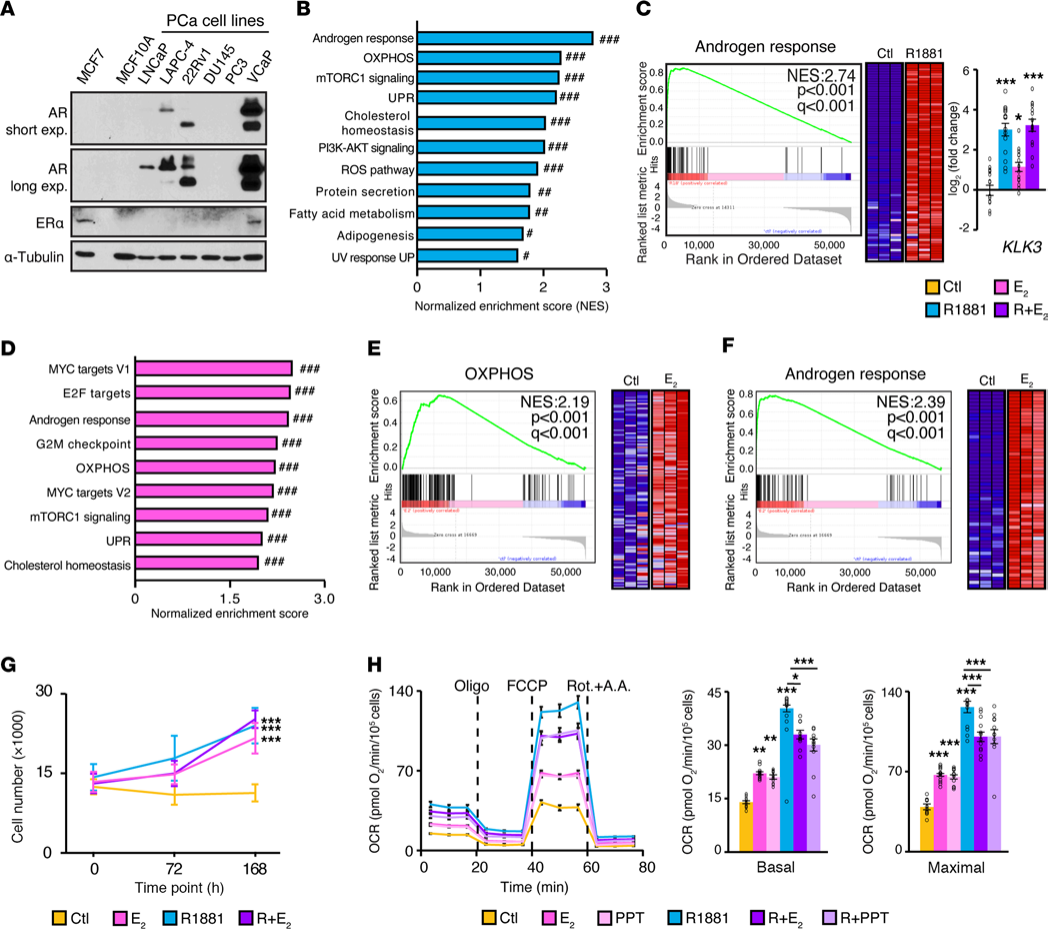
The ERα transcriptional program promotes PCa cell metabolism and proliferation. (**A**) Western blot of AR and ERα expression in in vitro models: 1 ERα-positive breast cancer cell line (MCF7), 1 ERα-negative mammary gland cell line (MCF10A), and 6 human PCa cell lines (α-tubulin was used as a loading control). exp., exposure. (**B**–**F**) RNA-Seq analyses of VCaP cells following 24 hours of treatment with vehicle, the synthetic androgen R1881, E_2_, or both (R + E_2_). (**B**) GSEA NES following treatment with R1881. (**C**) GSEA diagrams and heatmap for the androgen response gene set following treatment with R1881 and qRT-PCR analysis of *KLK3* expression (encodes PSA). Values are shown as the average with the SEM of 4 independent experiments performed in triplicate. (**D**) GSEA NESs showing enrichment following treatment with E_2_. ^#^*q* < 0.05, ^##^*q* < 0.01, and ^###^*q* < 0.001 (**B** and **D**). GSEA diagrams and heatmaps for the OXPHOS (**E**) and androgen response (**F**) gene sets following treatment with E_2_ in VCaP cells. For **C**, **E**, and **F**, the NES, *P* values, and *q* values are indicated on each diagram, and only core genes for each pathway are shown. (**G**) VCaP proliferation assay following treatment with either R1881, E_2_, or both. One representative experiment of 4 independent experiments is shown. Results are shown as the mean ± SEM (*n =* 6–8/treatment group). (**H**) VCaP OCR profiles following 72 hours of treatment with either R1881, E_2_, or both. Complete mitochondrial stress test results with basal and maximal OCR capacities are shown. Oligo, oligomycin; Rot.+A.A., rotenone + antimycin A. One representative independent experiment of 3 is shown. Data show the mean of normalized data to cell numbers ± SEM (*n =* 10–12/treatment). *P < 0.05, **P < 0.01, and ***P < 0.001, by 1-way ANOVA (**C**, **G**, and **H**).

We then assessed the estrogenic response in human PCa cell lines. Given the usage of nonspecific antibodies (7, 44, 45), conflicting reports were published regarding ERα and ERβ expression status in human in vitro PCa models. Consequently, we first verified the expression of both ERs in commonly used PCa cell lines using validated antibodies with appropriate controls such as ERα-positive (MCF7) and -negative (MCF10A) cell lines (Figure 4A). The majority of PCa cell lines tested did not express detectable/high protein levels of ERα, except VCaP cells. After longer film exposure, we also detected ERα expression in PC3 cells, but at very low levels (data not shown and ref. 7). The AR status of PCa cell lines could be clearly distinguished. ERβ expression was also evaluated with the anti–CWK-F12 (DSHB) antibody, validated for its specificity (44), but none of the cell lines tested displayed detectable protein levels (data not shown). As such, the heterogeneous expression of ERα observed in PCa cell lines partially mimicked the heterogeneity previously observed in patients (Figure 1).

Since VCaP expressed both the AR and ERα, we used this human PCa cell line to study the estrogen transcriptional response by RNA-Seq. It must be noted that VCaP cells were isolated from a patient’s vertebral metastasis after his cancer became resistant to ADTs and the anti-androgen flutamide; thus, this in vitro model was established, by definition, from a CRPC tumor (2, 46). After steroid deprivation for 48 hours, VCaP cells were treated for 24 hours with the synthetic androgen R1881, E_2_, or a combination of both, before RNA-Seq analyses (all significantly modulated genes are listed in Supplemental Table 5). AR activation induced a strong androgen response and also regulated multiple pathways linked to cell proliferation and metabolism, notably the mTORC1 signaling pathway, the OXPHOS gene signature, and the cholesterol homeostasis signature (Figure 4, B and C, and Supplemental Figure 5, A and B). Most of the regulated pathways were also observed in vivo in the normal mouse prostate (Figure 2) and in mouse PCa (Figure 3).

Multiple pathways regulated by E_2_ in VCaP cells were also shared with those induced by estrogens in mouse tumors. Indeed, signaling pathways linked to proliferation (MYC targets, G_2_M checkpoint), protein regulation (unfolded protein response [UPR] and mTORC1 signaling), and cholesterol homeostasis were upregulated following hormone treatment (Figure 4D and Supplemental Figure 5, A, C, and D). In particular, estrogens enriched the OXPHOS pathway in VCaP cells (Figure 4, D and E), as seen in vivo in mouse PCa (Figure 3) but not in the normal prostate (Figure 2). Finally, the androgen response, a tumorigenic pathway in VCaP cells, was also enriched with estrogens (Figure 4, D and F). Genes comprised in this pathway notably include *KLK3* (encodes PSA), which was significantly upregulated following each hormonal treatment (Figure 4C, right). This indicates that E_2_, just like androgens, has oncogenic functions in this cell line. As observed in vivo, the combination of both hormones led to enrichment of the same observed pathways seen with individual treatments (Supplemental Figure 5, A, E, and F). Altogether, these results confirm that E_2_ treatment induced a major transcriptional response in PCa cells, promoting oncogenic pathways and inducing the expression of metabolic genes important for PCa biology, such as genes implied in mitochondrial respiration (OXPHOS).

We next interrogated the functional effects of this transcriptional signature on cancer cell biology. As previously reported (47), E_2_ significantly stimulated VCaP cell proliferation (Figure 4G and Supplemental Figure 5G). Importantly, in this cell line that exhibits high AR dependency (2), the effect of E_2_ on proliferation was as strong as that of R1881. Note that other human PCa cell lines, such as DU145, 22Rv1, and LAPC-4 (see Supplemental Figure 5H and ref. 7), that do not express ERα did not show any significant modulation by either E_2_ or propyl pyrazole triol (PPT, a specific agonist of ERα), irrespective of their AR status. We next wanted to validate that E_2_ not only regulates the expression of genes associated with OXPHOS, but that it also functionally regulates mitochondrial activity. To do so, we measured the oxygen consumption rates (OCRs) of treated VCaP cells during a mitochondrial stress test (Figure 4H). As predicted from RNA-Seq, both androgens and estrogens increased the basal and maximal respiratory capacities of VCaP cells. Indeed, E_2_ treatment increased mitochondrial DNA content (Supplemental Figure 5I). The use of PPT also confirmed that this estrogenic regulation of mitochondrial respiration was ERα dependent. To the contrary, knockdown of *ESR1* with siRNAs abolished the E_2_-mediated induction of mitochondrial activity, further validating the specificity of this hormonal response (Supplemental Figure 5, J and K). Finally, the coactivation of both receptors also led to a significant increase in basal and maximal cell respiration compared with the control condition, although the effect of the cotreatment was not additive and led to a smaller increase of the OCR compared with androgens alone.

**Figure 5:**
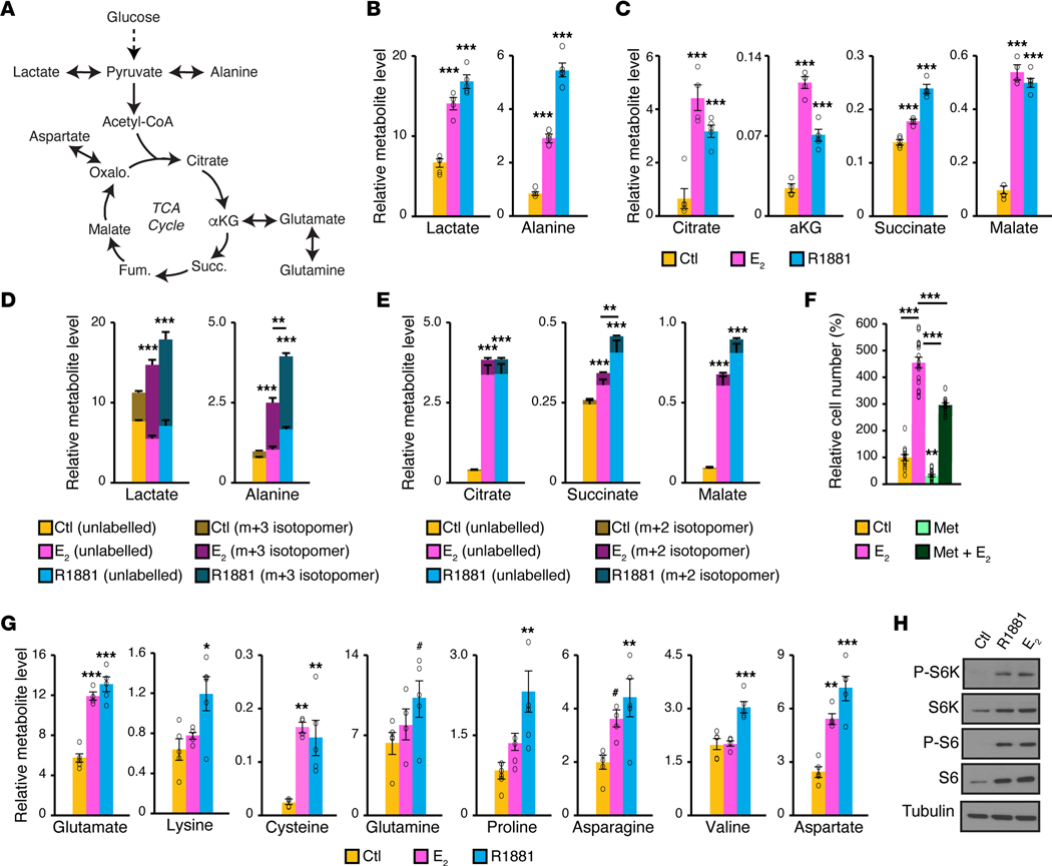
ERα activation induces cancer cell metabolism, notably by promoting glucose consumption and usage. (**A**) Schematic overview of glucose metabolism through glycolysis to allow pyruvate synthesis, which can then fuel the mitochondrial TCA cycle for respiration. Note that not all enzymatic reactions are shown (dashed lines symbolize intermediate steps). αKG, α-ketoglutarate; Succ., succinate; Fum., fumarate; Oxalo., oxaloacetate. (**B** and **C**) Quantification of lactate (**B**), alanine (**B**), and TCA cycle intermediates (**C**) in VCaP cells following 72 hours of treatment with E_2_ or the synthetic androgen R1881 by gas chromatography–mass spectrometry (GC-MS). (**D** and **E**) Quantification of ^13^C incorporation from ^13^C-glucose in lactate and alanine (**D**) and TCA cycle intermediates (**E**) in VCaP cells following 72 hours of treatment with E_2_ or R1881. ^13^C-glucose allowed the enrichment of a heavier isotopomer with a mass of plus 3 (m+3) for lactate and alanine and a mass of plus 2 (m+2) for citrate, succinate, and malate if it feeds the TCA cycle. (**F**) Changes in VCaP cell numbers following 168 hours of treatment with either E_2_, the inhibitor of mitochondrial respiration metformin (Met), or both (Met + E_2_). The changes in cell numbers were normalized in percentages according to the control treatment. Results are shown as the mean ± SEM of 2 independent experiments (*n =* 16/treatment group). (**G**) Quantification of amino acids connected to energy synthesis pathways in VCaP cells following 72 hours of treatment with E_2_ or R1881 by GC-MS. For **B**–**E** and **G**, results are shown as the mean ± SEM of 1 representative experiment (*n =* 5/conditions) of 3 independent experiments. (**H**) Western blot of the mTOR signaling pathway with phosphorylation of downstream targets (S6 and S6K) following hormone treatment. α-Tubulin was used as a loading control. *P < 0.05, **P < 0.01, and ***P < 0.001, by 1-way ANOVA, respective to control conditions or as indicated. For **D** and **E**, *P* values are only shown for metabolites with ^13^C labeling. ^#^*P* < 0.10 (**G**).

To further decipher the metabolic effects of androgens and estrogens, we conducted metabolomics analyses. First, the fate of pyruvate, the main product of glycolysis, was studied. Once synthesized, pyruvate can be converted into the amino acid alanine or be used to produce ATP, either through lactate synthesis or by directly fueling the TCA cycle that supports mitochondrial respiration (Figure 5A). Regardless of the hormone treatment, alanine and lactate (Figure 5B) and TCA cycle intermediates (Figure 5C), including citrate and malate, were all increased following AR or ERα activation. The observations that the levels of all TCA cycle intermediates measured were increased following treatment with E_2_ or R1881 (Figure 5C), thus fueling the electron transport chain to support mitochondrial respiration (Figure 4), are consistent with the RNA-Seq results (Figures 3 and 4). Interestingly, stable isotope tracer analyses using ^13^C-labeled glucose confirmed increased metabolic fluxes through aerobic glycolysis (lactate, Figure 5D, left), alanine synthesis (Figure 5D, right), and TCA cycle activity (Figure 5E), with both E_2_ and R1881 significantly inducing ^13^C enrichment of downstream intermediates. Some differences were observed, but mostly regarding the fold increase in metabolite levels. For example, androgens increased alanine levels by more than 5-fold compared with vehicle, as opposed to E_2_, which was increased more than 2-fold (Figure 5B, right) and consistent with a smaller flux of ^13^C from glucose into alanine (Figure 5D, right). These results show that E_2_ stimulation promoted PCa cell metabolism, notably by increasing glucose consumption and usage in cancer cells, as observed following AR activation. As such, we hypothesized that the E_2_-dependent metabolic program was essential for the E_2_-dependent activation of proliferation. Indeed, treatment with metformin, an inhibitor of mitochondrial respiration (48), significantly impaired the E_2_-mediated increase in proliferation, demonstrating that regulation of bioenergetic pathways by estrogens was essential to promote maximal cancer cell proliferation (Figure 5F).

Another important pathway induced at the mRNA level was the mTORC1 pathway, which is often associated with protein synthesis that requires energy and amino acids. Accordingly, all hormone treatments significantly increased the levels of the most detectable amino acids, including glutamate, asparagine, cysteine, proline, and aspartate (Figure 5G). Consequently, both androgens and estrogens promoted ATP-generating pathways, namely aerobic glycolysis and mitochondrial respiration pathways, and also stimulated biomass production through increased amino acid levels. In line with this hypothesis, E_2_ stimulation activated the mTOR signaling pathway, as shown by phosphorylation of its downstream targets S6 and S6K (Figure 5H), which is similar to the results obtained following AR activation (Figure 5H and as described previously in refs. 4, 49).

### Effect of anti-estrogen treatments in ERα-positive PCa.

**Figure 6:**
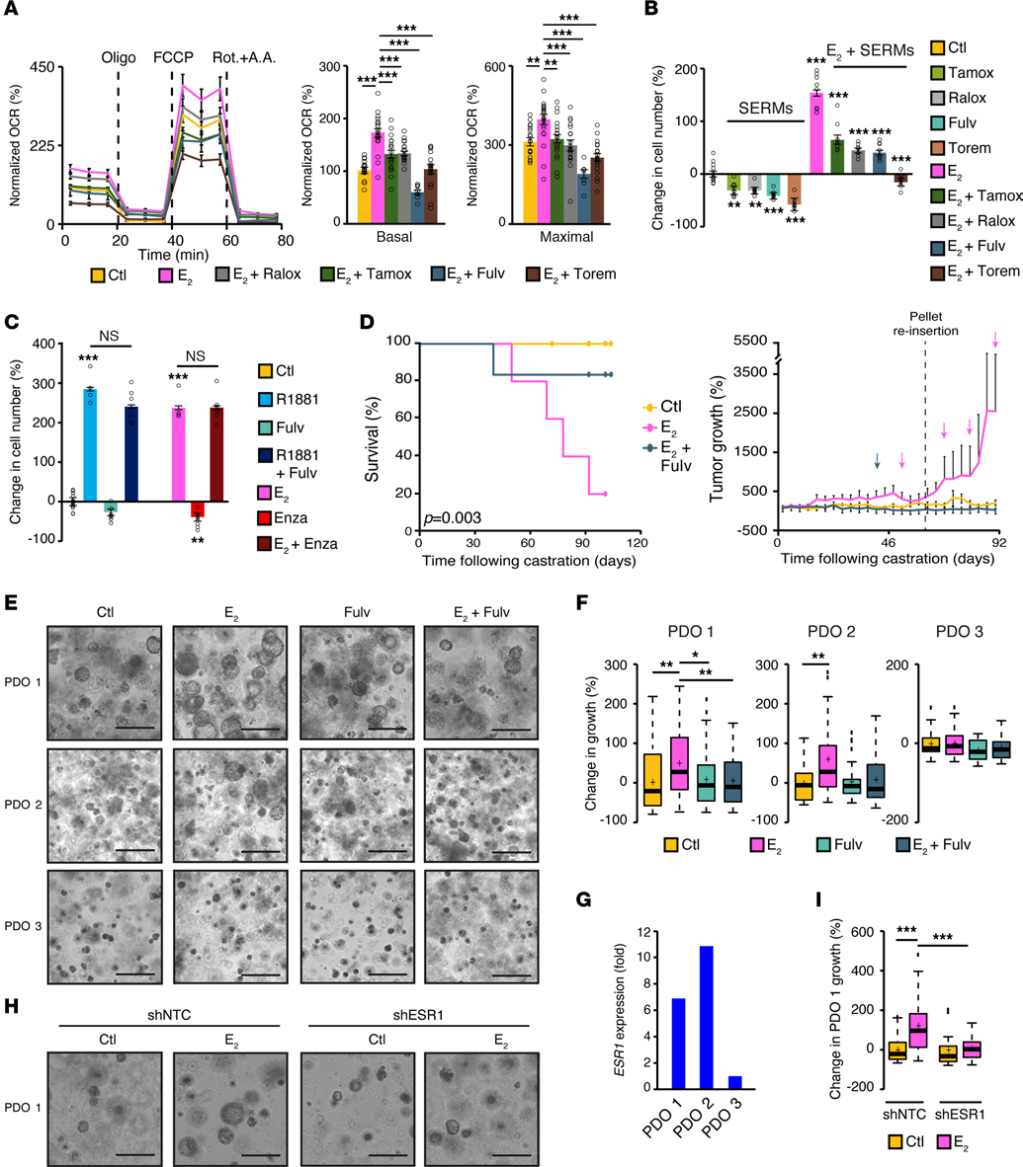
SERMs and fulvestrant inhibit E_2_-dependent stimulation of mitochondrial respiration, proliferation, and growth of PCa cells. (**A**) VCaP OCR profiles following a 72-hour treatment with E_2_, tamoxifen (Tamox), raloxifene (Ralox), toremifene (Torem), and fulvestrant (Fulv). Results from a complete mitochondrial stress test of 1 experiment are presented, with basal and maximal OCR capacities shown as the average of 2 of 3 independent experiments. Data indicate the mean ± SEM (*n =* 8–12/treatments per experiment). Changes in VCaP cell number following 168 hours of treatment with anti-estrogens cotreated with E_2_ (**B**), or with hormone cotreatment with fulvestrant or enzalutamide (**C**), normalized to control. One representative experiment of 3 independent experiments is shown. Data indicate the mean ± SEM (*n =* 6–8/condition). (**D**) Kaplan-Meier of survival and tumor growth of castrated mice with VCaP xenografts under either a placebo or E_2_ pellet treatment and injected weekly with vehicle or fulvestrant (*n* = 5–10 mice/condition). The log-rank test *P* value is shown. Changes in tumor growth were quantified on the basis of tumor volume at castration adjusted at 0%. Tumor growth is shown up to 90 days, at which point most E_2_-treated tumors were harvested. Colored arrows indicate mice reaching ethical limit points. (**E** and **F**) Bright-field images (**E**) and changes in organoid growth (**F**) of 3 PDO lines after 14–15 days of treatment with vehicle, E_2_, fulvestrant, or both. (**G**) qRT-PCR analysis of *ESR1* expression in the PDO lines shown in **E**. Results are shown as a fold change compared with PDO 3. (**H** and **I**) Bright-field images (**H**) and changes in organoid growth (**I**) in PDO 1 after 15 days of treatment with vehicle and E_2_, with and without *ESR1* knockdown. Scale bars: 300 μm (**E** and **H**). Results in **F** and **I** are shown as the mean ± SEM (*n =* 4 replicates/condition). NS, nonsignificant; *P < 0.05, **P < 0.01, and ***P < 0.001, by 1-way ANOVA.

Next, we wanted to determine whether targeting ERα could block the metabolic and proliferative effects of estrogens in PCa by using ERα-positive breast cancer drugs, such as the pure anti-estrogen fulvestrant and SERMs (tamoxifen, raloxifene, and toremifene). We performed a mitochondrial respiration study following cotreatment with SERMs or fulvestrant and estrogens (Figure 6A). As expected, E_2_ significantly increased the respiratory capacities of VCaP cells and, importantly, tamoxifen, raloxifene, toremifene, and fulvestrant were able to impair or completely block this increase of mitochondrial capacities (Figure 6A). In line with this finding, treatment with SERMs or fulvestrant blocked the E_2_-mediated stimulation of PCa cell proliferation (Figure 6B), consistent with an ERα-specific response, as shown using siRNAs against *ESR1* (Supplemental Figure 5I). Moreover, cotreatment with fulvestrant impaired the E_2_-dependent transcriptional regulation in VCaP cells, as validated by qRT-PCR (*PGR*, *E2F1, BRCA1*, and *KLK3*; Supplemental Figure 6A). Furthermore, cotreatment with fulvestrant blocked the E_2_-mediated increase in respiration and proliferation, without altering the AR-dependent effects (Figure 6C and Supplemental Figure 6B). Similarly, treatment with the anti-androgen enzalutamide did not block the estrogenic effect on respiration (Supplemental Figure 6C) or the E_2_-mediated increase in proliferation (Figure 6C), again demonstrating the specificity of the estrogenic response versus the AR signaling.

In addition, to reinforce the notion that the estrogen signaling pathway can bypass anti-androgen treatments, we also used a VCaP subline resistant to enzalutamide (formerly known as VCaP-ER [ref. 50], named herein VCaP-EnzR to avoid confusion). In these cells, and as observed in parental cells, we observed an induction of mitochondrial respiration and cancer cell proliferation following E_2_ exposure, demonstrating that the estrogen signaling pathway can conserve oncogenic functions even after the acquisition of EnzR (Supplemental Figure 6, D and E). The addition of the anti-estrogen fulvestrant affected this hormonal regulation, but treatment with enzalutamide, which specifically blocks the AR, had no effect on the estrogenic response. We then used parental VCaP cells in xenograft assays to evaluate the effect of E_2_ on PCa in an in vivo context. First, VCaP cells were injected into the flank of immunocompromised mice to allow tumor engraftment. When tumors became palpable, mice were castrated to ensure steroid deprivation. During surgery, hormone-releasing pellets were also inserted subcutaneously, and mice were separated into 2 groups, receiving either a placebo or an E_2_-releasing pellet. Importantly, in this context in which no more androgens were in circulation, the presence of estrogens induced the growth of VCaP xenografts despite castration (Figure 6D). Furthermore, treatment with fulvestrant blocked the VCaP xenografted cells from becoming resistant to surgical castration, despite the presence of E_2_. Altogether, these findings confirm the oncogenic characteristics of the estrogen signaling pathway in PCa, independently of the AR, and emphasize ERα’s potential as a therapeutic target for patients with ERα-positive PCa.

To further support the clinical efficiency of SERMs in primary human PCa, we conducted, as a proof of principle, a pilot study using PDOs from prostate tumor tissues. In 2 PDO series, we observed a significant increase in organoid growth following E_2_ treatment (Figure 6, E and F). Importantly, cotreatment with fulvestrant completely blocked this E_2_-dependent growth. Interestingly, the PDO 2 line originated from a patient who previously received neoadjuvant ADT prior to prostatectomy, thus suggesting that this PDO line could represent PCa transitioning to CRPC. In a third PDO line, however, we observed no positive regulation of growth by E_2_ (Figure 6, E and F). According to the differential response to E_2_, *ESR1* transcript levels (ERα mRNA) were much higher in the E_2_-responsive PDOs than in the E_2_-nonresponsive PDO (Figure 6G). Moreover, to confirm that the effect of E_2_ indeed occurred via the activation of ERα, we performed a knockdown experiment in an E_2_-responsive PDO line using a doxycycline-inducible shRNA against *ESR1* (Supplemental Figure 6F). The results showed that the induction of growth by E_2_ was abrogated following *ESR1* knockdown (Figure 6, H and I), further emphasizing the link between ERα expression and sensitivity to both E_2_ and anti-estrogens in PCa cells.

**Figure 7:**
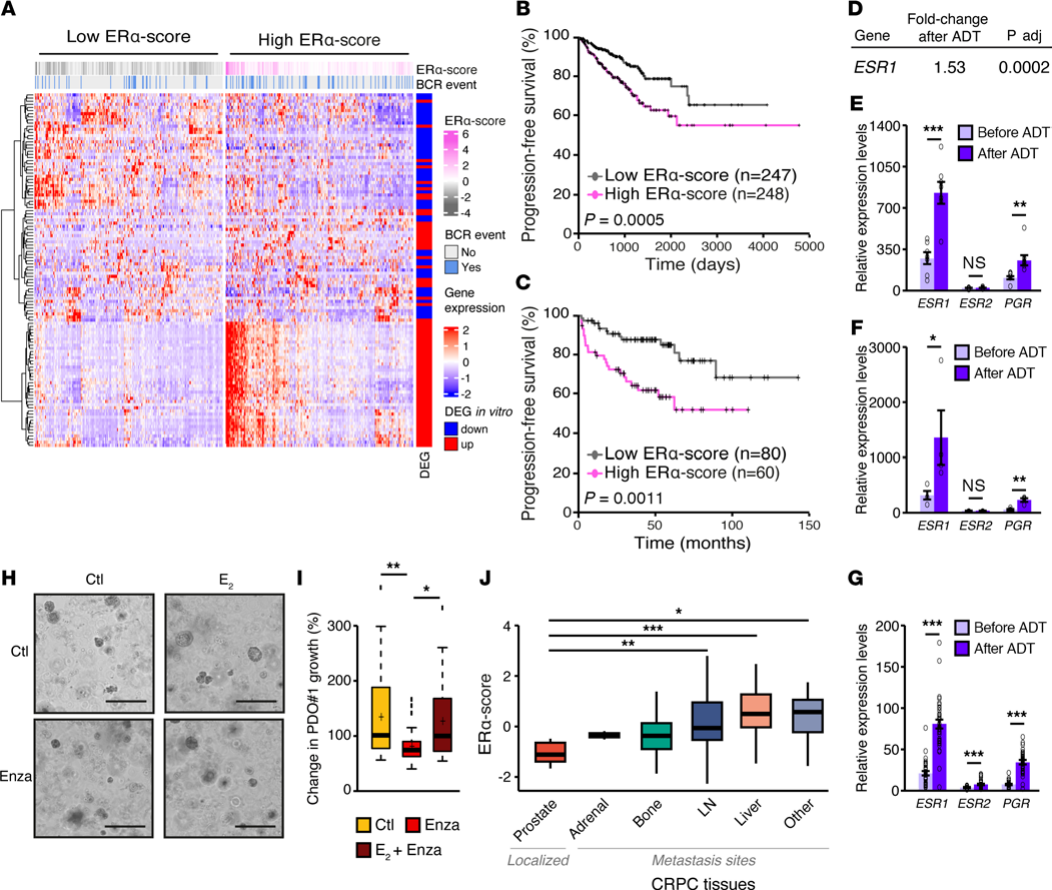
*ESR1* expression is increased following ADT, and its transcriptional signature is associated with PCa progression. (**A**) Heatmap of the ERα-score in patients from TCGA-PRAD data set (30, 31). The ERα-score is the predicted transcriptional activity of ERα. The legend shows DEGs with increased (red) or decreased (blue) expression following E_2_ treatment in VCaP cells. (**B** and **C**) Kaplan-Meier of BCR-free survival following surgery for patients from TCGA-PRAD (**B**) and the Taylor et al. (**C**) data sets, separated by high and low ERα-scores. The log-rank *P* values are shown. (**D**) *ESR1* (encodes ERα) expression in PCa tumors before and after ADT in the Eur Uro 2017 data set (52). adj, adjusted. (**E**) *ESR1*, *ESR2*, and *PGR* gene expression in PCa tumors before and after ADT in the Eur Uro 2014 data set (53) (*n =* 7 paired samples). (**F**) *ESR1*, *ESR2*, and *PGR* gene expression in PCa tumors before and after ADT plus docetaxel in the BioMed Central (BMC) cancer data set (54) (*n =* 4 paired samples). (**G**) *ESR1*, *ESR2*, and *PGR* gene expression in PCa tumors before and after ADT in the GSE183100 data set (*n =* 73 samples). (**H** and **I**) Bright-field images (scale bars: 300 μm) (**H**) and changes in organoid growth (**I**) of the PDO 1 line after treatment with vehicle and the anti-androgen enzalutamide (Enza) cotreated or not with E_2_. (**J**) ERα-score in the SU2C data set (55), separated by tumor localization in the prostate (*n =* 5) and metastases in either adrenal glands (*n =* 2), bone (*n =* 82), lymph nodes (LN) (*n =* 79), liver (*n =* 26), and other sites (*n =* 14). NS, nonsignificant; *P < 0.05, **P < 0.01, and ***P < 0.001, by 1-way ANOVA (**I** and **J**) or 2-tailed Student’s *t* test, as appropriate (**E**–**G**).

With this vision of targeting ERα for therapeutic purposes, we then leveraged TCGA PCa RNA-Seq data set (30, 31). On the basis of the E_2_-dependent signature obtained with human VCaP cells (presented in Figure 4), we designed an ERα-score that was applied to this RNA-Seq data set (Figure 7A). Interestingly, most genes upregulated by E_2_ in VCaP cells were also expressed at higher levels in patients with a strong ERα-score — and vice-versa for E_2_-dependent downregulated genes. Patients with a high ERα-score, indicative of a high transcriptional (metabolic) ERα signature, had lower progression-free survival rates (Figure 7B). These results were further validated using the Taylor et al. (51) data set, again demonstrating that patients with high ERα-scores had shorter BCR-free survival rates (Figure 7C, and Supplemental Figure 6G). These results are in line with those shown in Figure 1, bridging the levels of ERα to its cancer-specific signature and PCa progression in patients.

Finally, we wanted to assess whether targeting ERα could also apply to patients with CRPC, as suggested by our findings with the VCaP xenografts (Figure 6D). To this end, we reanalyzed RNA-Seq data from 3 published studies that investigated, in a small number of patients, the transcriptomic changes occurring before and after ADT (52–54). In the study by Shaw et al., expression of the *ESR1* gene increased by 1.5-fold following ADT (adjusted [adj.] *P* = 0.0002), suggesting that *ESR1* is induced in cancer cells that survive ADT (Figure 7D). In line with this hypothesis, *ESR1* relative expression was also significantly increased, by 3.1- and 4.4-fold, after ADT in 2 other data sets (Figure 7, E and F, and Supplemental Figure 6, H and I). The ERα target gene *PGR* was also significantly increased in that context, supporting the hypothesis that both ERα expression and activity are increased during evolution to CRPC. In contrast, the *ESR2* gene, which encodes ERβ (see Discussion), was barely detectable and did not change upon ADT. In a fourth RNA-Seq data set consisting of 73 samples, expression of *ESR1* and *PGR* was again significantly increased in tumor samples following ADT (Figure 7G). These analyses suggested that ERα activation could be linked to treatment resistance in CRPC. Indeed, treatment with enzalutamide alone inhibited PDO growth, but E_2_ stimulation was able to bypass this inhibition and still induce PDO growth (Figure 7, H and I). In line, in the Stand Up 2 Cancer (SU2C) RNA-Seq data set, a higher ERα-score was observed in CRPC metastases, including lymph node and liver metastases, compared with localized tumors (Figure 7J) (55). Altogether, these results demonstrate that the ERα transcriptional signature and expression are associated with PCa progression and resistance to treatments targeting the AR signaling pathway.

## Discussion

The current study demonstrates the heterogeneity of ERα protein levels in human PCa tumors, as well as the effect of ERα, when expressed, on disease progression. Mechanistically, transcriptomic analyses revealed that estrogens promote oncogenic and metabolic gene signatures in prostates of WT and PCa mouse models, as well as in ERα-positive VCaP cells. Accordingly, bioenergetic flux and metabolomics analyses confirmed metabolic regulation by estrogens. Consequently, E_2_ treatment led to the positive regulation of proliferation and growth in VCaP cells (in vitro and in vivo) and PDOs that displayed ERα protein or mRNA expression. Conversely, this induced oncogenic phenotype was blocked by anti-estrogen and SERM treatments. Altogether, the current study demonstrates the role of ERα in promoting PCa cell proliferation and metabolism, as well as its potential to become a personalized therapeutic target for PCa.

Since the role of estrogens in the prostate and PCa was unclear, we first wanted to dissect the transcriptional functions of the estrogen signaling pathway using in vitro and in vivo preclinical models. In all the ERα-positive studied models, treatment with E_2_ induced important transcriptional changes, mostly by modulating genes associated with oncogenic pathways such as MYC and mTORC1, and promoted cancer cell metabolism, notably by increasing the expression of genes involved in mitochondrial respiration. Importantly, these experiments showed substantial overlap in biological pathways modulated by both the androgen and estrogen responses. Indeed, AR is a well-known regulator of the mTORC1 signaling pathway, as well as an important modulator of PCa cell metabolism, notably by promoting mitochondrial biogenesis and activity (3, 4, 56). The AR was shown to fuel mitochondrial respiration through pyruvate usage by regulating the mitochondrial pyruvate carrier gene *MPC2* (57). In the present study, we also observed this androgen-dependent modulation of mitochondrial activity in normal and tumoral contexts, along with positive regulation by E_2_, which highlights the estrogen signaling pathway as a new key orchestrator of prostate and PCa cell metabolism. One of the important pathways induced by estrogens was OXPHOS, as evidenced by transcriptional signatures and changes in mitochondrial respiration. Altogether, our results demonstrate that estrogens promoted a specific transcriptional profile in PCa, with both distinct and overlapping genes and regulatory functions similar to those regulated by androgens and the AR. We thus believe that the activation of ERα partially mimics the action of androgens and, consequently, promotes PCa cell proliferation and disease progression.

Despite having been studied for decades, the effectiveness of anti-estrogen therapies in the context of PCa is still unclear. We believe this could be partly explained by the lack of an accurate assessment of ERα expression status in prostate tumor cells before treatment administration. Indeed, in the breast cancer field, ERα protein levels are first evaluated in tumors to determine if they belong to ERα-positive or -negative subtypes, and this analysis then dictates the adequate treatment. Here, using a clinically validated antibody, several tumors appeared to be ERα-negative, as previously reported (21), whereas other tumors showed positive ERα nuclear staining. This approach could be easily implemented in the clinical setting for PCa prognostication and treatment, since it is routinely performed for breast cancer. Thus, we believe that assessing ERα subtypes will allow the selection of PCa patients with the best chance of responding to anti-estrogenic therapies. Given that molecules targeting ERα have already been approved for ERα-positive breast cancer and various other clinical indications, if our hypothesis is validated in prospective clinical studies, stratification of PCa by ERα status to repurpose anti-estrogens could lead to additional therapeutic options in the PCa clinical landscape.

Since ERβ is also expressed in the prostate, we cannot rule out the possibility that some of the transcriptional changes observed in vivo were ERβ-dependent and not ERα-dependent. Based on work with βER-KO mice, it is often thought that ERβ plays a tumor suppressor role (45). However, this point is still controversial, as other research groups, using slightly different mouse models, did not observe this relationship between ERβ and PCa (58–61). Future work is still required to fully dissect the prostate-specific response to E_2_ and the functional interaction between ERα and ERβ. In the present study, as most pathways transcriptionally regulated by E_2_ were associated with oncogenic functions, and since we observed barely to no detectable levels of ERβ/*Esr2* in our models, we believe they are mostly regulated by ERα. Moreover, in 2 of 3 different data sets in which *ESR2* expression was investigated, there were no significant post-ADT changes in patients, as opposed to the significant increase detected in *ESR1* relative expression (Figure 7, E–G). These data also highlighted the very low expression levels of *ESR2* in PCa tumors, further confirming that the changes observed following estrogen stimulation in our different models were induced by ERα activation.

Indeed, we used several genetic and pharmacological tools to ensure that the estrogenic response was specific to ERα. These tools included siRNAs, an shRNA, and cells that do not express ERα (similar to a KO). ERα-positive models included the mouse normal and tumoral prostate, parental VCaP and VCaP-EnzR cells, and some PDO lines, while ERα-negative models included PCa cell lines such as 22Rv1 and DU145, as well as 1 PDO line. The E_2_-dependent transcriptional, metabolic, and pro-proliferative functions were observed in ERα-positive models but not in ERα-negative models. The only exception was LNCaP cells, an ERα-negative cell line but with a mutated AR that can bind to E_2_ (but not to PPT; Supplemental Figure 6). For pharmacological tools, we used the specific ERα ligand PPT, as well as tamoxifen, raloxifene, toremifene, and fulvestrant, molecules that have been well characterized in vitro and in vivo in patients. Most of these molecules have distinct structures (Supplemental Figure 7). The fact that the combination of several ligands (notably the ERα-specific agonist PPT) with distinct molecular structures led to the same conclusions further supports the ERα-specific functions. Overall, using various approaches and models, we clearly demonstrated that activation of the estrogen signaling pathway and the beneficial effects of targeting this pathway in preclinical models, are always observed in an ERα-dependent manner.

Altogether, the results presented here emphasize the need to perform new clinical studies using molecules targeting the estrogen signaling pathway specifically in ERα-positive tumors. We believe that these molecules would be beneficial for both castration-sensitive and castration-resistant PCa, notably in combination with ADT and/or anti-androgens. Several lines of evidence support this hypothesis, such as the TMA results for tumors from patients who received several rounds of treatments targeting the AR, such as anti-androgen treatment, before surgery. In this cohort, the active form of ERα (nuclear ERα) was associated with metastases and death following several years of ADT, thus clearly linking ERα signaling in the context of ADT and lethal CRPC. Second, results from Figure 7, comprising 4 different clinical data sets, show that ADT increased the expression levels of *ESR1*, which encodes ERα. These results support the idea that ERα is indeed highly relevant during PCa treatment and evolution toward CRPC, as demonstrated by the ERα score enrichment in castration-resistant metastases and by the VCaP xenograft’s growth induced by E_2_ in castrated mice. Third, using VCaP cells, which were isolated from a CRPC tumor, and 1 hormone-naive ERα-positive PDO line, we showed that E_2_ could bypass AR signaling to promote proliferation, growth, and metabolism, even when the anti-androgen enzalutamide was present. These experiments demonstrate that, at least in preclinical models, ERα activity can bypass AR blockade. This is in line with results from our TMA’s validation cohort with neoadjuvant ADT and from RNA-Seq data before and after ADT. Interestingly, in a recent multisample, whole-genome analysis, *ESR1* amplification was observed during the transition of cancer cells to metastatic CRPC (62), supporting our results. *ESR1* amplifications are rare (or even absent) in primary hormone-naive PCa tumors on the cBioPortal from TCGA consortium, but *ESR1* amplification was detected in metastatic CRPC samples, further strengthening the link between the estrogen signaling pathway and PCa progression following AR-targeted treatments. Future clinical studies considering the ERα status are thus needed to maximize the potential of repurposing of SERM drugs and anti-estrogens for the treatment of PCa.

Overall, our study supports the clinical relevance of ERα as a potential therapeutic target for the management of ERα-positive PCa tumors. Given the availability of both ERα clinical-grade antibodies and ERα-targeted drugs, repurposing of SERMs and anti-estrogens could rapidly be tested in prospective clinical studies in combination with anti-androgens in patients with PCa and a progressive disease.

## Methods

All materials and methods can be found in the Supplemental Methods.

### Sex as a biological variable.

We only studied biological males, given that the prostate is specific to biological males.

### Statistics.

For all details regarding statistics, please refer to the Supplemental Files. In brief, a *P* value of 0.05 or less or an adjusted *P* value for multiple testing of 0.05 or less was considered significant. When comparing 2 groups, a 2-tailed Student’s *t* test was used. When comparing 3 or more groups, 1-way ANOVA was used with Dunnett’s (referring to 1 control group) or Tukey’s (comparing several groups) analysis. For survival analyses, log-rank and Cox regression analyses were performed.

### Study approval.

All human and animal studies were approved by the appropriate IRBs (respectively, the Research Ethics Committee of the CRCHUQ-UL, Quebec City, Québec, Canada; the Université Laval Research and Ethics Animal Committee, Quebec City, Québec, Canada). For PDOs, written informed consent from patients was received before participation in the project (CRCHUQ-UL, 2021-5661). For mouse work, the study was approved by the Université Laval Research and Ethics Animal Committee (CHU-22-1206) in Quebec City.

### Data availability.

In vivo and in vitro RNA-Seq data sets generated for the current study are available in the Gene Expression Omnibus (GEO) database (GEO GSE254635 and GSE256370). Other data can be found in the Supporting Data Values file in the supplement or by contacting the corresponding author.

## Author contributions

EAW designed the study. CL, LG, GHCS, CW, LB, HH, HB, CJ, LFP, RC, KG, AL, PC, CM, CB, MP, and EAW conducted experiments, acquired data, and provided reagents. HH, HB, CA, JR, E Latulippe, AB, PT, CG, YF, FP, LL, and É Lévesque acquired human tissues for TMA and PDO analyses. CL and EAW analyzed data, designed figures, and wrote the manuscript. All authors reviewed and approved the manuscript.

## Supplementary Material

Supplemental data

Unedited blot and gel images

Supplemental table 1

Supplemental table 2

Supplemental table 3

Supporting data values

## Version 1 04/16/2024

In-Press Preview

## Version 2 06/03/2024

Electronic publication
