# Supplementary material for: The estrogen signaling pathway reprograms prostate cancer cell metabolism and supports proliferation and disease progression
Source: J Clin Invest. 2024 Apr 16;134(11):e170809. doi: 10.1172/JCI170809 (PMC11142735; doi:10.1172/JCI170809)
Supplement: Supplemental data [file jci-134-170809-s113.pdf]

Supplemental Material

The estrogen signaling pathway reprograms prostate cancer cell metabolism and supports cancer cell proliferation and disease progression

Camille Lafront, Lucas Germain, Gabriel H. Campolina-Silva, Cindy Weidmann, Line Berthiaume, Hélène Hovington, Hervé Brisson, Cynthia Jobin, Lilianne Frégeau-Proulx, Raul Cotau, Kevin Gonthier, Aurélie Lacouture, Patrick Caron, Claire Ménard, Chantal Atallah, Julie Riopel, Éva Latulippe, Alain Bergeron, Paul Toren, Chantal Guillemette, Yves Fradet, Martin Pelletier, Clémence Belleannée, Frédéric Pouliot, Louis Lacombe, Éric Lévesque, and Étienne Audet-Walsh

Supplementary material and methods

Supplementary discussion

Supplementary Figures S1 – S7

Supplementary Tables S1, S2, S6, S7, and S8 (*Supplemental Tables S3, S4 and S5 are submitted individually*)

SUPPLEMENTARY MATERIAL AND METHODS

Cell culture

LNCaP, LAPC-4, 22Rv1, DU145, PC3, VCaP, MCF7, MCF10A, and MDA-MB-231 cell lines were all obtained from ATCC and were cultivated in media and conditions recommended by ATCC. VCaP-EnzR were generously provided by P. Toren's lab and were cultivated in DMEM-F12 supplemented with 15% fetal bovine serum and 1% sodium pyruvate + enzalutamide (1 µM; ApexBio). All cells were kept in incubators at 37°C and 5% CO_2 and cultured for no more than four months. Mycoplasma testing was performed at least every three to four months in the laboratory. The confluence was maintained below 75% and media were changed every two to three days. For steroid deprivation, cells were seeded in phenol red-free culture media with 1% penicillin + streptomycin, 1% sodium pyruvate, and supplemented with 5% charcoal-stripped serum (CSS) for 48h before conducting experiments.

Mouse models and animal facility

Mice used for this study (models C57BL/6J *Pten*$^{fl/fl}$ [WT] and C57BL/6J PB-Cre4$^{+/-}$;*Pten*$^{fl/fl}$ [PCa] (1)) were obtained from The Jackson Laboratory and bred and housed at the animal house of the CRCHUQ-UL, according to the guidelines and regulations of the Canadian Council on Animal Care (CCAC). They followed a 12h light:12h dark cycle at 22°C, and all work performed was approved beforehand by Université Laval Research and Ethic Animal Committee (CHU-12-2206).

Tissue microarrays of patients and statistical analyses

We used a previously established and validated tissue microarray dataset (Supplemental Table S1) (2, 3). Four µm thick paraffin sections of the TMA were deparaffinized, hydrated, and stained for ERα expression at the Pathology Laboratory of L'Hôtel-Dieu de Québec (CHUQc-UL), using the routine protocol for human ERα-positive breast cancer determination on Dako Autostainer with Dako anti-ERα antibody (clone EP1). Digital images were visualized with the software NDP.view2 (Hamamatsu) for visual scoring by two experienced readers (H. Brisson and H. Hovington) blinded to tumor characteristics and clinical outcomes. The frequency of nuclear staining was scored on the whole TMA core's surfaces as 0 if absent in all cells, 1 if 1-4 cells were positive, 2 if 5-9 cells were positive, and 3 if >10 cells were positive per 100 cells. These numbers represented 0% positive cells for a score of 0, 1-4% positive cells for a score of 1, 5-10% positive cells for a score of 2, and >10% positive cells for a score of 3. For every tumor, 3 cores were available, and the average of ERα score was used for analysis. For statistical analysis, nuclear ERα expression was dichotomized as low expression, corresponding to an average score of less than 1, and high expression, corresponding to a score of ≥ 1-3.

The validation cohort was a TMA comprised of tumor samples from 41 additional men with high-risk PCa. These patients underwent neoadjuvant treatment with hormonal therapy prior to their surgery, as described previously (3). Specific clinical details of this cohort are available in Supplemental Table S2. As for the discovery cohort, ERα staining was performed at our local pathology laboratory using the Dako anti-ERα antibody (clone EP1). Images were scored by the same reviewers, again blinded to tumor characteristics and clinical outcomes. Survival analyses were then performed using the same threshold, as established in the discovery cohort, of the ERα score (average of the three cores/tumor).

Western blots

For whole cell lysates using mouse prostate or cell lines, samples were processed as previously described (4) and resuspended in buffer K supplemented with protease and phosphatase inhibitors before performing Western Blots with primary antibodies: α-tubulin (11H10, #2125, Cell Signaling), AR (clone EPR1535[2], ab133273, Abcam), human ERα (D-12, sc-8005, Santa Cruz), mouse ERα (clone 6F11, #MA1-27107, Invitrogen), ribosomal protein S6 (C-8, sc-74459, Santa Cruz) and phospho-S6 (p-S6; 50.Ser 235/236, sc-293144, Santa Cruz). For the mTOR signaling investigation, the other antibodies used were p70 S6 kinase (S6K; E8K6T, #34475, Cell Signaling) and phospho-p70 S6 kinase (p-S6K; Thr389, #9205, Cell Signaling).

Mouse tissue immunostaining and histology

Mouse prostates of at least 24 weeks of age were formalin-fixed, paraffin-embedded, and thereafter sectioned at 5 µm using a microtome (HistoCore MULTICUT 14051856372, Leica). The slides were heated at 60°C for 15 min, then deparaffined and rehydrated. For Hematoxylin and Eosin (H&E), staining was realized using the H&E Staining kit (ab245880) and slides were mounted with Permount (ThermoFisher Scientific). For IHC, antigen demasking was carried out in 10 mM citrate buffer (pH 6.0) at 110°C for 10 min in a pressure cooker. Tissues sections were then sequentially treated for 15 min with a 0.3% hydrogen peroxide solution, followed with an incubation of 1h in TBS 1X buffer containing 5% goat serum and 1% BSA. Then, the slides were incubated overnight at 4°C with primary antibodies against ERα (clone 6F11, #MA1-27107, Invitrogen ; dilution 1/50) or AR (clone EPR1535[2], ab133273, Abcam; dilution 1/50). After washes with TBS 1X, slides were exposed for 1h at room temperature with either goat anti-mouse or anti-rabbit secondary antibodies (both at 1/200; Biotin-SP [long spacer] AffiniPure Goat Anti-Mouse IgG [H+L] [code 115-065-003] and Biotin-SP [long spacer] AffiniPure Goat Anti-Rabbit IgG [H+L] [code 111-065-003], Jackson ImmunoResearch Lab). The slides underwent another incubation with the VECTASTAIN Elite ABC reagent (Vector Laboratories), then the precipitation reaction was carried out with DAB (0.05% w/v) mixed with hydrogen peroxide (0.01% v/v) in 0.05M Tris-HCl buffer. Counterstaining was done with modified Mayer's Hematoxylin, then the slides were dehydrated before being mounted with Permount. The number of cells with ERα nuclear labeling was determined using image-assisted analysis with ImageJ (5). For this purpose, the number of positive and negative cells was determined in 10 acini/ducts randomly selected from each prostatic lobe (anterior, dorsolateral and ventral), encompassing proximal, median, and distal regions. On average, 2,700 cells were considered per animal, and the data were represented as the percentage of cells with ERα nuclear staining ($n = 5$ for WT, $n = 4$ for PCa). ERα staining intensity was quantified in each prostate lobe by measuring the pixel intensity in 200-immunoreactive epithelial cell nuclei (6). For this analysis, cell nuclei were not counterstained with hematoxylin. Results were expressed as percentages calculated relative to the highest value.

In vivo RNA-seq data and analyses

Sixteen male C57BL/6J *Pten*$^{fl/fl}$ (WT) and 14 male C57BL/6J PB-Cre4$^{+/-}$;*Pten*$^{fl/fl}$ (PCa) mice of at least 24 weeks of age were castrated. After 72h, to ensure steroid deprivation, they received s.c. injections of either vegetable oil mixed with ethanol 96% (vehicle; 1:10 v/v), E_2 (30 µg/g of mouse weight; Sigma), testosterone (30 µg/g of mouse weight), or a combination of both hormones. After 24h, prostates were harvested for RNA purification using the QIAGEN RNeasy Mini Kit, and RNA quality was controlled on TapeStation 2200 bioanalyzer (Agilent). Then, mRNA library preparation was performed with the NEBNext Ultra II Directional RNA library prep

kit before sequencing at the Next Generation Sequencing Platform of the CRCHUQ-UL with a NovaSeq 6000 (paired-end, 100 bp sequence length, depth of 20-25M reads). Raw sequencing quality was verified with FastQC (7) and MultiQC (8), then data were cleaned using Trim Galore/Cutadapt (9, 10). The reads were then pseudo-aligned with Kallisto (11) using the Gencode vM25 murine reference transcriptome. The aligned and quantified data were analyzed using DESeq2 (12). Genes significantly modulated were selected based on an FDR value < 0.05 and a fold-change ≥ 1.75 or ≤ -1.75 (Supplemental Table S3 for WT, and Supplemental Table S4 for PCa). The Gene Set Enrichment Analysis (GSEA) tool was used (13), along with Metascape for enrichment network visualization (14).

In vitro RNA-seq data and analyses

After steroid deprivation of VCaP cells, fresh CSS-phenol red-free medium was added, containing either vehicle (ethanol 96%), E_2 (10 nM) and/or the synthetic androgen R1881 (10 nM; Toronto Research Chemicals). Triplicates were performed for each treatment group. VCaP cells were harvested 24h later for RNA purification with the QIAGEN RNeasy Mini Kit. Sequencing and analyses were performed as per in vivo RNA-seq (see above), except that the reads were aligned using the Gencode v27 human reference transcriptome. Genes significantly modulated were selected based on an FDR value < 0.1 and a fold-change ≥ 1.75 or ≤ -1.75 (Supplemental Table S5).

In vivo single-cell RNA-seq data and analyses

Four male C57BL/6J PB-Cre4$^{+/-}$;*Pten*$^{fl/fl}$ (PCa) mice of at least 24 weeks of age underwent castration. Three days later, they were injected s.c. with either vegetable oil mixed with ethanol 96% (vehicle; 1:10 v/v) or E_2 (30 µg/g of mouse weight). After 24h, prostates were harvested, individually minced, then followed multiple digestion steps based on the murine prostate cell isolation protocol established by Frégeau-Proulx *et al.* (15, 16). Each digestion media was supplemented with the ROCK inhibitor Y-27632 dihydrocloride (10 µM; Tocris) and DNAse I Solution (0.1 mg/mL; Sigma). Additionally, digestion media of E_2 samples were supplemented with 1 nM E_2. After straining, cells were sorted by FACS (FACSAria™ Fusion Flow Cytometer) with viability marker 7-AAD in EDTA-free buffer, and kept in PBS 1X + BSA 0.4% at a concentration of 500-1,000 cells/µL until sequencing. Cells were then rapidly processed at our local Genomic Centre at the Centre de recherche du CHU de Québec – Université Laval, using the scRNA-seq library preparation kit from 10X Genomics as per the manufacturer's instructions, before sequencing with a NovaSeq 6000. Sequencing data were then analyzed using Nextflow (17) with nf-core (18) single cell pipeline "scrnaseq" v2.3.2 (19). Results from Cellranger were exported and analyzed using R package Seurat v4.3.0.1 (20). Cells from the same treatment group were merged, and then control- and estradiol-treated cells were associated using Seurat's *IntegrateData* method. Clusters were annotated using Karthaus and colleagues' gene list (21). Differential expression (control versus E_2) was measured after selecting specific clusters using Seurat's FindMarkers method with MAST parameter, with no fold-change threshold. The output was either filtered for downstream analyses (heatmaps) or ordered by decreasing fold-change as input for GSEA pre-ranked (13) using mouse's hallmark gene set. Genes were considered differentially expressed with an adjusted *p*-value (FDR) inferior to 0.05 (no filtration was done for absolute fold-change).

Weight ratio of prostate/total mouse body and hormone serum concentrations

Sixteen male C57BL/6J *Pten*$^{fl/fl}$ (WT) were treated as described above with control, testosterone, E_2, or both hormones for 24h, following three days of castration to ensure steroid deprivation in circulation. Mice were then anesthetized with isoflurane and blood was collected by cardiac puncture. Following coagulation at room temperature for 30 min, samples were then centrifugated to isolate serum. Both testosterone and E_2 were then quantified in serum by liquid chromatography with tandem mass spectrometry (LC-MS-MS), with methodology from Tiwari and colleagues (22).

Xenograft assays

Nude mice (Crl:CD1-*Foxn1*^{nu}) were first injected 1M of VCaP cells resuspended in a 1:1 ratio with growth factor reduced Matrigel (Corning) on the right flank, for a total injection volume of 200 µL per mouse. Tumors were then allowed to engraft. When tumors became palpable and measurable (between 1 to 2 months following VCaP cells injection), mice were castrated to ensure steroid deprivation of both androgens and estrogens and to mimic androgen deprivation therapy (ADT) in patients. During surgery, a placebo or an E_2-releasing pellet (0.5 mg/pellet, 60-day release; Innovative Research of America) was implanted subcutaneously. Then, one week after surgery, animals began to receive weekly s.c. injections of fulvestrant (mixed 1:1 v/v with vegetable oil, 0.25 mg/g of mouse weight for a maximal dosage of 8 mg per injection; TEVA Pharmaceutical Industries), or its vehicle (mix of ethanol [5%], benzyl alcohol [5%], benzyl benzoate [7,5%], castor oil [32,5%], and vegetable oil [50%], all in v/v), based on the injectable fulvestrant's nonmedical ingredients listed by TEVA Pharmaceutical Industries. Animals were then followed for the duration of the study where their weight and tumor mass were measured twice per week by experienced technicians blinded to treatment groups, until reaching ethical limit points. A new pellet was re-implanted 60 days after castration to ensure constant hormonal release throughout the experiment. Exclusion criteria comprised issues unrelated to VCaP xenografted tumors, including problems at castration, development of ulcer at the injection site, or if mice were found dead during the study without a detectable growing tumor following castration. The injections caused ulcerations over time at the site of injection, and the study was stopped in agreement with our local ethical committee after reaching significance between our experimental groups.

ERα-score analysis in the TCGA and the Taylor *et al.* PCa datasets

Public TCGA prostate adenocarcinoma sequencing data were downloaded from the UCSC Xena plateform during the Spring 2023 (https://xena.ucsc.edu/) and cBioPortal (https://www.cbioportal.org/) to get clinical and expression data (default RSEM expression data). Only primary tumors were kept, filtering out normal and metastatic samples. The ERα-score was evaluated using genes detected to be either up- or down-regulated in VCaP cells following E_2 treatment compared to control cells. Only genes detected in the TCGA dataset were considered (n = 113). The score was defined as the geometric mean, using R package MetaIntegrator's *geomean* method, of the up-regulated genes minus the geometric mean of down-regulated genes. This score was standardized across all samples using the z-score method. Samples with a score superior to the median were considered as "high ERα-score" whereas those with a score inferior to the median were considered as "low ERα-score". Kaplan Meier survival curves were generated considering the biochemical recurrence (BCR, representative of the progression-free interval [PFI] indicator).

For clustering of the ERα signature genes, the Ward.D2 method was used. The same approach was performed using the dataset from Taylor *et al.* (23).

Quantitative RT-PCR

After treatment for 24h and RNA purification with either QIAGEN RNeasy Mini Kit or EZ-10 Spin Column Total RNA Miniprep Super Kit, the cDNA of prostate mouse samples and of VCaP cells was generated with the LunaScript RT SuperMix Kit (New England Biolabs). These samples were then used for quantitative RT-PCR (qRT-PCR) with the Luna Universal qPCRMasterMix (New England Biolabs). After obtaining Ct values, delta delta Ct were calculated and normalized to LOG fold-change using either two to four mouse housekeeping genes (*Pum1*, *Tbp*, *Eef2*, and *Leng8*) or two to three human housekeeping genes (*PUM1*, *TBP* and *ACTN)*. For PDOs (with or without shRNA), RNA extraction was carried out with the Monarch Total RNA Miniprep kit (New England Biolabs), and cDNA was synthetized with the LunaScript RT SuperMix Kit. Gene expression was normalized to power using *PUM1* and *TBP* housekeeping genes. All primers sequences can be found in Supplementary Tables S6 and S7. Two to three technical replicates were performed for each sample in qRT-PCR analyses.

DNA ratio experiments in VCaP cells were carried following 72h of treatments (either vehicle [ethanol 96%] or E_2 [10 nM]). DNA was extracted with the DNeasy Blood & Tissue kit from QIAGEN, then samples were used for quantitative PCR experiments (same methodology as described above). Human primers sequences targeting specific genomic (*HPCAL4*, *EP300*) and mitochondrial genes (*CYTB*, *ND1*) are detailed in Supplemental Table S7.

Extracellular Flux Analyses

Parental VCaP and VCaP-EnzR cells were directly seeded in Seahorse XFe96 microplates. After steroid deprivation for 48h, cells were treated either with: vehicles (ethanol 96% and DMSO), E_2 (10 nM), R1881 (10 nM), the ERα-specific ligand PPT (5 nM; 1,3,5-tris(4-hydroxyphenyl)-4-propyl-1H-pyrazole; Santa Cruz Biotechnology), the anti-estrogen fulvestrant (19 nM or 1 μM; ICI 182,780; Santa Cruz Biotechnology), 4-OH-tamoxifen (100 nM; Santa Cruz Biotechnology), raloxifene hydrochloride (285 nM; Tocris), toremifene citrate (10 μM; Santa Cruz Biotechnology), or enzalutamide (10 μM). For co-treatment experiments with SERMs or anti-androgens, E_2 and R1881 were used at a concentration of 5 nM. Three days later, media were replaced by Seahorse XF assay medium (RPMI with no phenol red or minimal DMEM without HEPES, supplemented with 1% penicillin and streptomycin, 1 mM sodium pyruvate, 10 mM glucose and 2 mM glutamine, pH adjusted at 7.4). After equilibration, the plate was transferred to the XFe96 instrument (Agilent/Seahorse) to measure oxygen consumption rates (OCR) using a standard mitochondrial stress test (24, 25). After the assays, cell number was determined using CyQUANT (Invitrogen) to normalize data.

For knockdown experiments of *ESR1* in parental VCaP, cells were firstly transfected in phenol red-free media supplemented with 5% CSS, with a mix of OPTIMEM media (Gibco), Hiperfect (QIAGEN) and siRNA (either siESR1 [ON-TARGETplus Human *ESR1* siRNA; Horizon Discovery] or siC [ON-TARGETplus Non-targeting Control Pool; Horizon Discovery]). The next day, transfected cells were seeded in Seahorse XFe96 microplates along with treatments, either with vehicle (ethanol 96%), E_2 (10 nM) or R1881 (10 nM). The extracellular flux experiments and cell quantification were carried as described above.

Metabolomics with Gas Chromatography-Mass Spectrometry (GC-MS)

After steroid deprivation for 48h, media were changed, and VCaP cells were treated with either vehicle (ethanol 96%), E_2 (10 nM), and/or R1881 (10 nM). Three days later, cells and media were harvested for GC-MS analysis, as previously described (16, 26), using an Agilent 8890 GC equipped with a DB5-MS+DG capillary column coupled to an Agilent 5977B MS instrument. Analyses were performed using the MassHunter Workstation Software (Agilent) and the NIST/EPA/NIH Mass Spectral Library (NIST 2017).

For stable isotope tracer analyses, cells were firstly plated in standard phenol red-free culture media supplemented with 5% CSS for at least 48h. Then, standard media was renewed with treatments described above. One day later, media were changed for phenol red-free DMEM without glucose, supplemented with 5% CSS, 2 mM glutamine, 1% penicillin + streptomycin and 1% sodium pyruvate. ^{13}C-labelled glucose (10 mM; ACP Chemicals) was added, along with treatments previously described. Two days later, after a total of three days of hormonal treatments and 48h of incubation with ^{13}C-labelled glucose, cells and media were harvested for GC-MS analysis of specific metabolite isotopomers as previously described (16, 26).

Primary culture and patient-derived organoids (PDOs) experiments

PDOs were developed from prostate biopsies of consenting subjects in the context of a project approved by the research ethics committee of the CRCHUQ-UL (2021-5661) that followed the standards described in the Declaration of Helsinki, using the isolation protocol developed by Frégeau-Proulx and colleagues (16). In brief, prostate tissue samples were taken from freshly removed radical prostatectomy specimens in neoplastic regions that indicated tumorigenesis as later confirmed by pathology reports, under the supervision of experienced pathologists (C. Atallah, J. Riopel and É. Latulippe). Following enzymatic digestions of prostate samples, a single-cell suspension was obtained and seeded for primary culture. We used the tumor samples from patients CW488T, CW530, and CW492T, named herein as PDO#1, PDO#2, and PDO#3, respectively.

To study the impact of E_2 and other treatments, primary PCa cells were plated in droplets of 40 μL containing 75% growth factor-reduced Matrigel and 25% medium containing cells (15,000 cells/droplet) as described previously (16). Then, 24h after seeding, media were changed for complete media supplemented with R-Spondin, Noggin and R1881 (10 nM), and containing either vehicles (ethanol 96% and DMSO), E_2 (5 nM), fulvestrant (1 μM) or enzalutamide (10 μM). Media were renewed with treatments every two to three days. Quantification assays were performed on Day 14 or 15 using an EVOS M5000 Imaging System (ThermoFisher Scientific). Measures of organoid diameters and calculation of areas for growth quantification were performed at several fields/droplets using ImageJ.

For shESR1-mediated knockdown experiments in PDO #1, a similar methodology as described in Gonthier and colleagues (27) was employed. Briefly, after selecting sequences targeting ESR1 from the RNAi Consortium (TRC) whole-genome library (listed in Supplemental Table S8), the shRNA was cloned in the EZ-Tet-pLKO-Puro vector (Addgene plasmid # 85966; Watertown, MA, USA), then transfected into Lenti-X HEK293T cells (along with psPAX2 and pMD2.G) in order to harvest the viruses four days later. For Matrigel droplets seeding, PDO #1 cells were directly resuspended in a mix of 25% supernatant-containing viruses (either shESR1 or shRNA Non-Target Control [NTC], sequence published by Lu et al. (28)) and incubated 30 min at RT before adding 75% growth-factor reduced Matrigel. A day later, media were renewed with doxycycline hyclate (100 ng/mL; Sigma) to induce the shRNA. Then, 48h following plating, treatments were added, either vehicle (ethanol 96%) or E_2 (5 nM), along with doxycycline. Media

was changed every two to three days with doxycycline and treatments. Quantification analyses were performed at Day 15, as described above.

Hormonal treatments for in vitro proliferation assays

After steroid deprivation for 48h, parental VCaP cells were treated with vehicles (ethanol 96% and DMSO), E_2 (0.1-10 nM), R1881 (10 nM), fulvestrant (1 µM), 4-OH-tamoxifen (500 nM), raloxifene hydrochloride (500 nM), toremifene citrate (10 µM), enzalutamide (10 µM), or metformin (2 mM; Sigma). For co-treatment experiments with SERMs or anti-androgens, E_2 and R1881 were used at a concentration of 5 nM. In the case of VCaP-EnzR cells, they were treated with either vehicles (ethanol 96% and DMSO), E_2 (5 nM), fulvestrant (1 µM), or enzalutamide (10 µM) following 48h of steroid deprivation. As for the other cell lines used (MCF7, LNCaP, LAPC-4, 22Rv1, PC3, DU145), they were treated with either the vehicles (ethanol 96% and DMSO), E_2 (10 nM), and PPT (1 nM) after steroid deprivation in phenol red-free media for 48h. Media were renewed every two to three days until the end of the assay. Cell number was calculated using crystal violet assay as previously described (29).

Statistics

For differences between two conditions, statistical significance was assessed using a Student's T test. XL STATS was used for survival analyses, including Kaplan Meier curves, log rank test, and Cox regression (see figure legends for the description of covariables). For the study of ERα protein expression levels in the TCGA cohort (30), ERα (ESR1) protein levels were downloaded from the cBioPortal platform in March 2023 (31, 32), along with clinical data related to biochemical recurrence. Only patients with information on ERα protein levels, time to PSA testing, and recurrence information were kept for analysis (n = 308). ERα protein levels were dichotomized as low (quartiles 1-3) or high (quartile 4) for analysis. Prism10 was used for multiple group comparisons, using 1-way ANOVA with post hoc tests (Tukey, Dunnett, or Benjamini, Krieger and Yekutieli).

SUPPLEMENTARY DISCUSSION

There has been considerable attention regarding ERα expression in the prostate and in PCa in the last decades. Initial studies demonstrated that it is mostly expressed in the stromal compartment (33-41), with lower or absent expression in the epithelium. Results presented in the current study are consistent with previous reports, as ERα was at higher levels in stromal cells and detected more often in the stromal compartment. Even when considered as positive in tumor cells, only a low percentage of these cells showed ERα nuclear staining (Supplemental Fig. S1). This is probably why ERα expression levels in tumor cells was overlooked in previous investigations and that most attention was given to ERα activity in stromal cells. Moreover, note that the antibody targeted against ERα used in the current study is approved for clinical purposes (for breast cancer) and has thus been thoroughly validated.

Regarding the role of ERα in stromal cells, studies have found that it is implied in the prostate development (42). Indeed, specific knock-out of *Esr1*, encoding ERα in mice, showed a disruption in prostate branching in the stromal compartment, but not in the epithelium (43). In a similar way, the impact of estrogen imprinting on prostate development seems to be mediated by stromal ERα. As such, neonatal exposure to estrogenic compounds leads to an increase of ERα stromal expression, which can then disrupt epithelial cell differentiation and cause epithelial hyperplasia and inflammation later in life (41, 44).

These effects induced by stromal ERα have been thought to affect the prostate epithelial compartment through paracrine signaling (33, 41, 44-47). Indeed, it is believed that part of the estrogenic impact observed in epithelial cells is mediated by stromal ERα (48), and therefore that both epithelial and stromal ERα are responsible for the estrogenic response in the epithelium (46). One example of this signalization would be the paracrine secretion of the protein ENO1 by activated stromal ERα, which then promotes epithelial cell migration (49).

In the context of PCa, the impact of stromal ERα on disease progression is still unclear. On the one hand, a study revealed that there is a weaker prostate stromal ERα expression in ethnic groups with higher risk to develop PCa (50), and that ERα+ cancer-associated fibroblasts (CAFs), following estrogen stimulation, seems to suppress tumor invasion (51, 52). This would suggest that stromal ERα have a protective effect on tumorigenesis. On the other hand, other reports demonstrated that estrogens stimulate the proliferation of stromal cells and ERα+ CAFs (53, 54), which could instead favor PCa development. As such, much remains to be done to fully comprehend the impact of stromal ERα in prostate development and in PCa, as well as the functional interaction between the estrogen signaling pathways in stromal and epithelial cells.

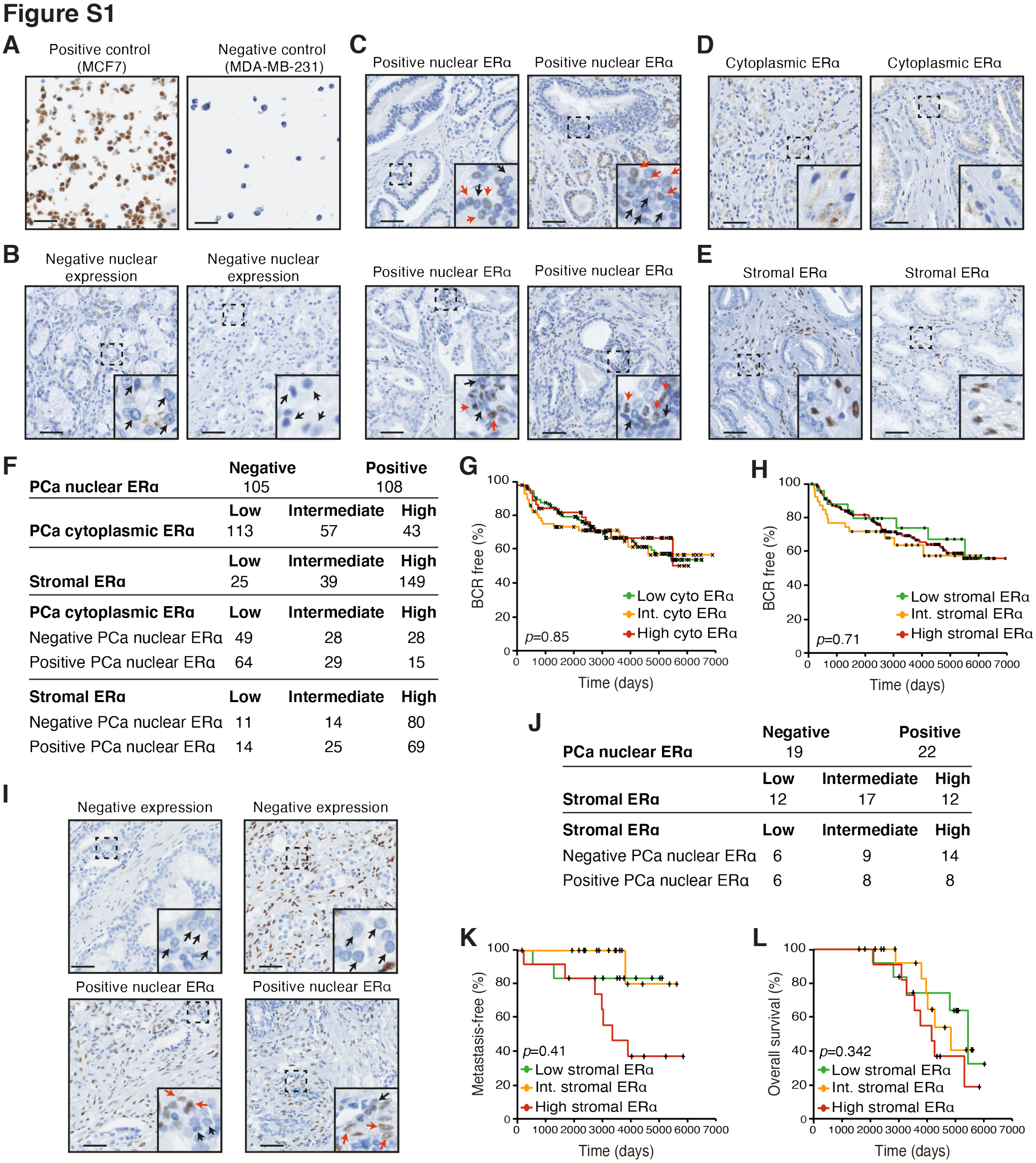

Fig. S1. (**A**) Validation of ERα antibody in immunohistochemistry (IHC) using the positive control MCF7, an ERα-positive human breast cancer cell line, and the negative control MDA-MB-231, an ERα-negative human breast cancer cell line. Scale = 50 µm. (**B-H**) Histological analysis of ERα in

the Belledant *et al.* TMA cohort (2). Representative images of ERα-negative (**B**), or ERα-positive nuclear (**C**), ERα-positive cytoplasmic (**D**), or ERα-positive stromal (**E**) staining in human PCa samples (each images representing a different patient). For nuclear ERα, examples of positive (red arrow) or negative (black arrow) staining are shown in the insets. Scale = 50 µm. (**F**) Categorization of biopsies samples regarding their cytoplasmic, nuclear, and stromal ERα expression (either negative or positive, or low, intermediate or high). Numbers indicate the number of patients in each category. (**G**, **H**) Kaplan-Meier survival analysis in patients with low, intermediate, or high levels of ERα cytoplasmic (**G**) or stromal (**H**) levels. Survival was defined as biochemical recurrence (BCR)-free survival following radical prostatectomy. The log-rank test *p* value is shown inset. (**I-L**) Histological analysis of ERα in a second independent TMA cohort from patients that received neoadjuvant hormono-therapy. (**I**) Representative images of ERα-negative or ERα-positive nuclear staining in human PCa samples (each images representing a different patient). Examples of positive (red arrow) or negative (black arrow) staining are shown in the insets. Scale = 50 µm. (**J**) Categorization of biopsies samples regarding their nuclear and stromal ERα expression (either negative or positive, or low, intermediate or high). Numbers indicate the number of patients in each category. (**K**, **L**) Kaplan-Meier survival analysis in patients with low, intermediate, or high levels of ERα stromal levels regarding development of metastasis (**K**) and overall survival (**L**). The log-rank test *p* value is shown inset.

Figure S2

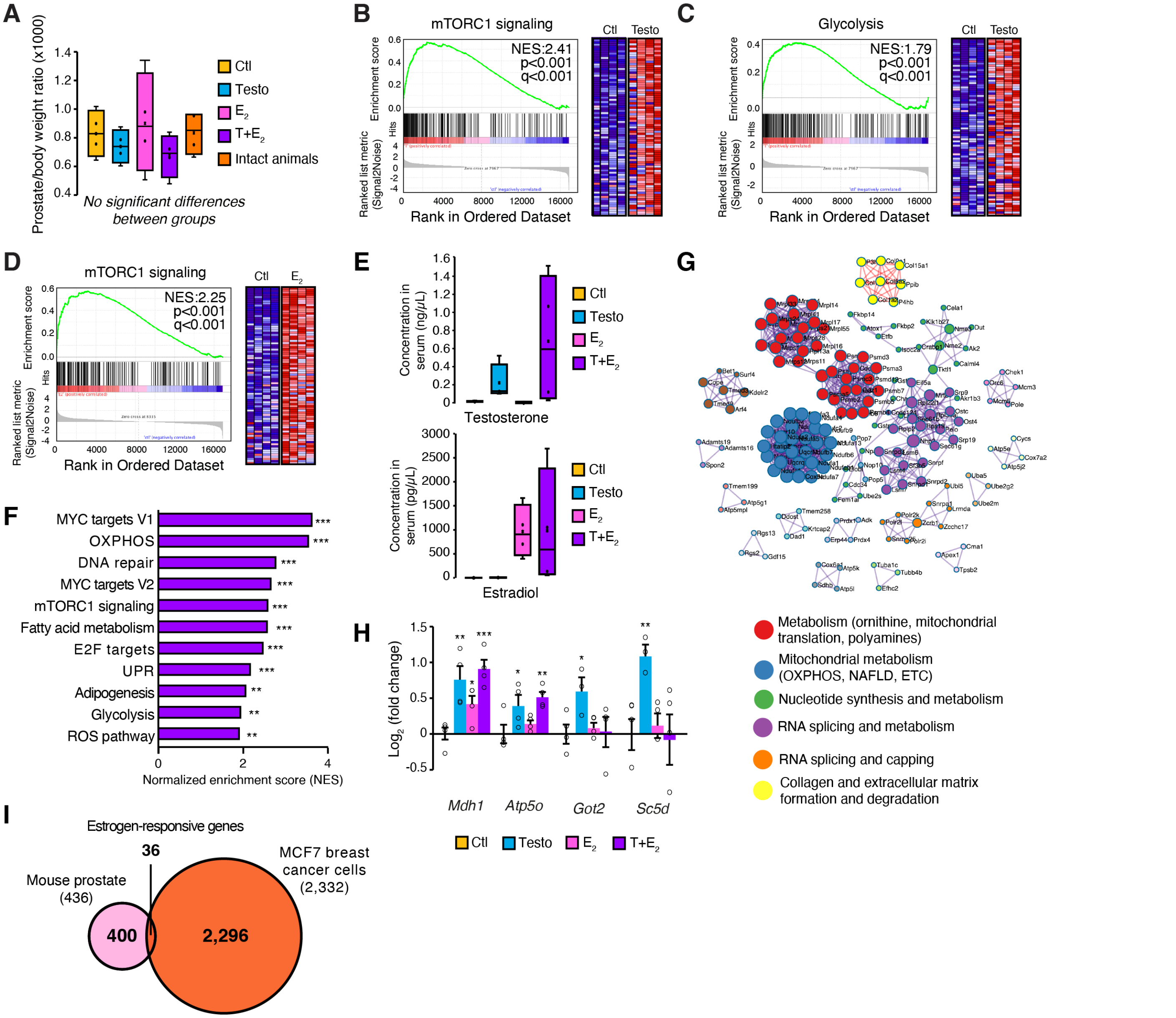

Fig. S2. (**A**) The prostate weight over total body weight ratio, with and without castration and hormonal treatments, of C57BL/6J *Pten*$^{fl/fl}$ (WT) mice (n = 3-4 per condition). Animals were castrated and three days later, to ensure steroid deprivation in circulation, they received s.c. injections with vehicle (Ctl), testosterone (Testo), estradiol (E_2) or both hormones (T+E_2). Twenty-four hours later, animals, either intact or castrated with hormonal injections, were weighted then sacrificed, and their prostate tissues were harvested and weighted in order to calculate

prostate/body weight ratio. (**B**, **C**) Gene Set Enrichment Analysis (GSEA) diagrams and heatmaps for the mTORC1 signaling (**B**) and the glycolysis (**C**) gene sets following treatment with testosterone in vivo in prostates of WT mice. Normalized enrichment score (NES), *p*-values and false discovery rates (FDR, *q*-values) are indicated on each diagram. Only core genes of each pathway are shown. (**D**) GSEA diagram and heatmap for the mTORC1 signaling gene set following treatment with E_2 in same model. NES, *p*-values and *q*-values are indicated on the diagram. Only core genes of the pathway are shown. (**E**) Concentration of circulating testosterone (up) and estradiol (down) in serum of castrated WT mice, 24h *post*-injection of either vehicle, Testo, E_2 or T+E_2. (**F**) NES of GSEA pathways enriched following treatment with T+E_2 compared to control, Testo, and E_2 treatments in normal murine prostate. $^*q < 0.05$, $^{**}q < 0.01$ and $^{***}q < 0.001$. (**G**) Metascape enrichment network visualization of the upregulated genes by T+E_2 compared to control in normal murine prostate, showing inter-clusters similarities of enriched genes notably in cell metabolism. Legend only shows major biological pathways. (**H**) qRT-PCR validation of metabolic genes significantly up-regulated by androgens (*Mdh1*, *Atp5o*, *Got2* and *Sc5d*), estrogens (*Mdh1*) or co-treatment (*Mdh1* and *Atp5o*) in normal murine prostate. Results are shown as the mean ± standard error of the mean (S.E.M.), $n = 4$ mice/group treatment $^*p< 0.05$, $^{**}p< 0.01$ and $^{***}p < 0.001$. (**I**) Venn diagram indicating only a small overlap between estrogen-responsive genes (comprising both significantly up- and down-regulated genes following treatment) in the MCF7 breast cancer cell line, using the dataset from (55), and in the normal mouse prostate. Circle and overlap sizes are proportional to the number of genes.

Figure S3

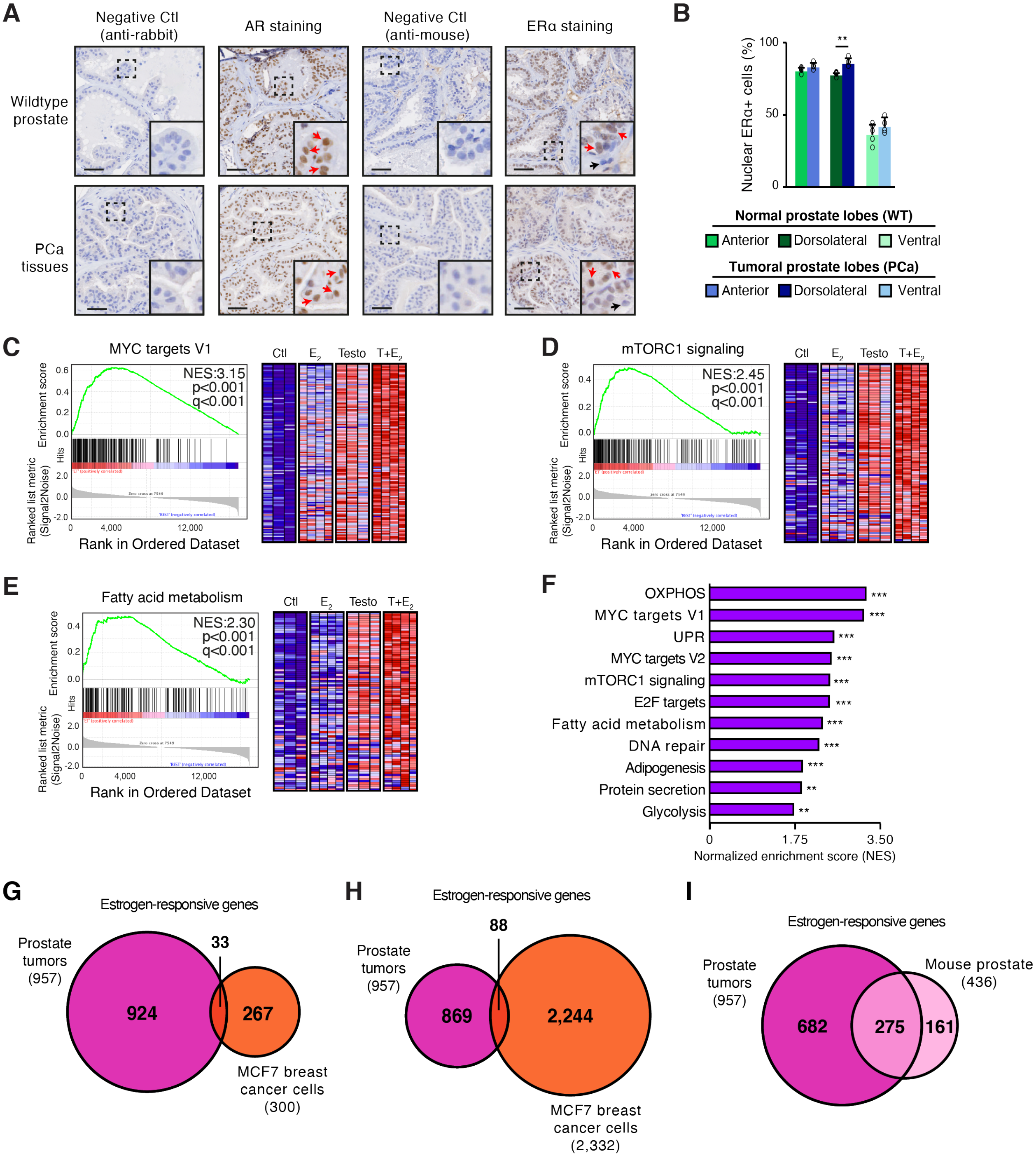

Fig. S3. (**A**) Representative images of immunohistochemistry (IHC) of AR and ERα in prostate from mice with or without PCa (models PB-Cre4$^{+/-}$;*Pten*$^{fl/fl}$ and C57BL/6J *Pten*$^{fl/fl}$, respectively). Red arrows in AR and ERα staining indicates positive nuclear protein levels, while black arrows

ERα staining indicates negative cells. Negative controls for each antibody (anti-rabbit for AR and anti-mouse for ERα) are shown. Scale = 50 µm. (**B**) Quantification of nuclear ERα-positive (ERα+) cells in the different lobes (anterior, dorsolateral and ventral) of the normal mouse prostate and in mice with PCa (n = 5 of each lobe for WT, n = 4 of each lobe for PCa). (**C**-**E**) Gene Set Enrichment Analysis (GSEA) diagrams and heatmaps for the MYC targets V1 (**C**), mTORC1 signaling (**D**), and fatty acid metabolism (**E**) gene sets following T+E_2 treatment in vivo in prostates of PCa-developping mice. Normalized enrichment score (NES), p-values and false discovery rates (FDR, q-values) are indicated on each diagram (results are compared to all three other treatment conditions). Only core genes of the pathway are shown. (**F**) NES of GSEA analysis enriched following treatment with T+E_2 in PCa-developing mice. *$q < 0.05$, **$q < 0.01$ and ***$q < 0.001$. (**G**) Venn diagram indicating a small overlap between estrogen-responsive genes in breast cancer cells (MCF7), using the dataset from (56), and in PCa-developing mice. Circle and overlap sizes are proportional to the number of genes. (**H**) Venn diagram indicating only a small overlap between estrogen-responsive genes (comprising both significantly up- and down-regulated genes following treatment) in the MCF7 breast cancer cell line, using the dataset from (55), and in mouse tumor tissues. Circle and overlap sizes are proportional to the number of genes. (**I**) Venn diagram indicating an important overlap between estrogen-responsive genes in the normal mouse prostate (from Fig. 2) and PCa-developing mice. Circle and overlap sizes are proportional to the number of genes.

Figure S4

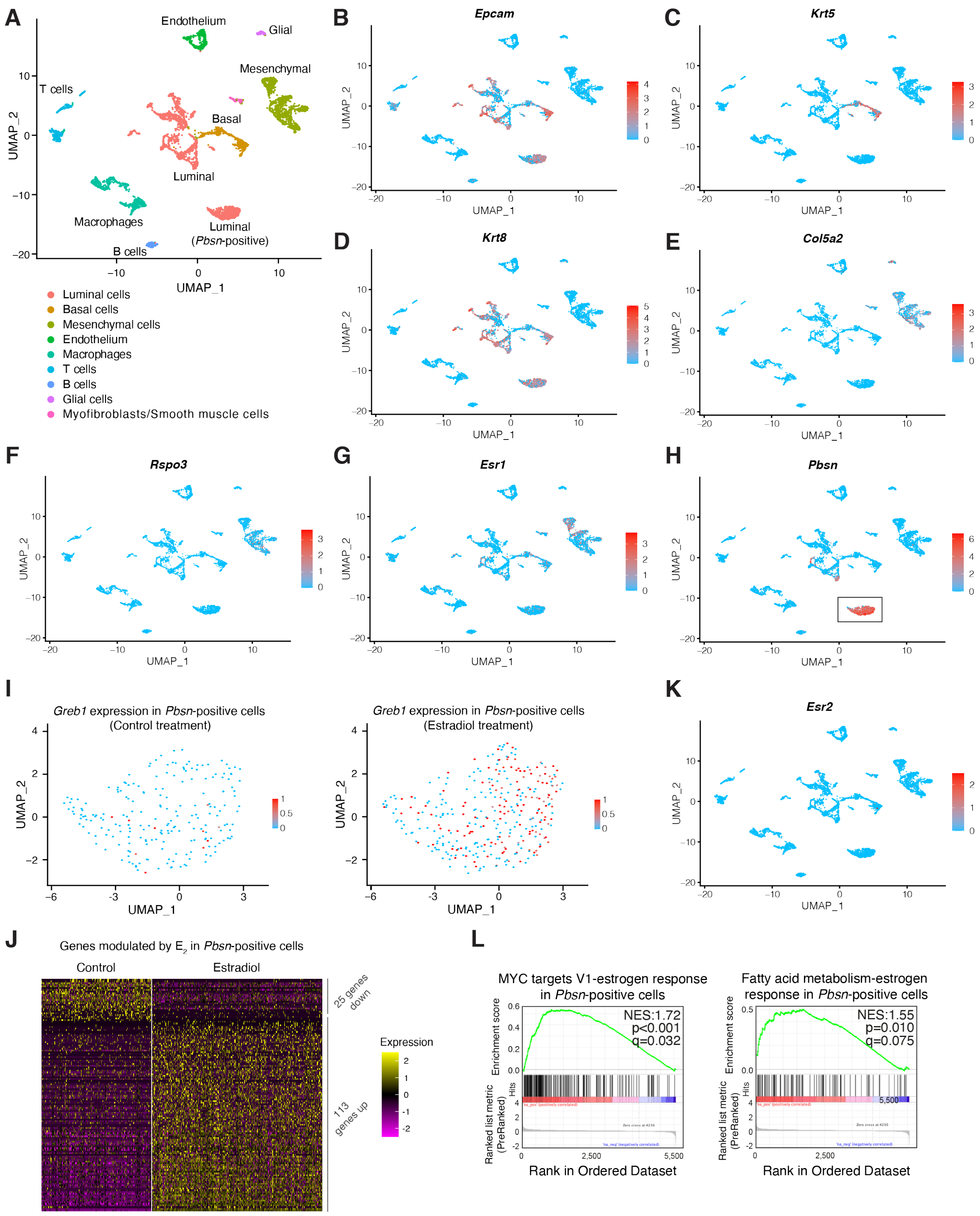

A
Endothelium
Glial
Mesenchymal
T cells
Basal
Luminal
Macrophages
Luminal (Pbsn-positive)
B cells
UMAP_1
UMAP_2
Luminal cells
Basal cells
Mesenchymal cells
Endothelium
Macrophages
T cells
B cells
Glial cells
Myofibroblasts/Smooth muscle cells
B
Epcam
C
Krt5
D
Krt8
E
Col5a2
F
Rspo3
G
Esr1
H
Pbsn
I
Greb1 expression in Pbsn-positive cells (Control treatment)
Greb1 expression in Pbsn-positive cells (Estradiol treatment)
K
Esr2
J
Genes modulated by E2 in Pbsn-positive cells
Control
Estradiol
25 genes down
113 genes up
Expression
L
MYC targets V1-estrogen response in Pbsn-positive cells
NES:1.72
p<0.001
q=0.032
Fatty acid metabolism-estrogen response in Pbsn-positive cells
NES:1.55
p=0.010
q=0.075
Enrichment score
Hits
Ranked list metric (PreRanked)
Rank in Ordered Dataset

Fig. S4. (**A**) Annotated UMAP of 5,483 individual cells from four tumoral murine prostate samples (two control and two E_2-treated) showing cell populations from single-cell analyses. Prostate samples were taken from mouse model PB-Cre4$^{+/-}$;*Pten*$^{fl/fl}$ with prostate-specific *Pten* loss (driven by the *Pbsn* promoter) leading to tumorigenesis. Annotations have been made using Karthaus *et al.* gene list (57). (**B-H**) Expression in log scale of counts per 10k (CP10K) + 1 pseudo count for *Epcam* (**B**), *Krt5* (**C**), *Krt8* (**D**), *Col5a2* (**E**), *Rspo3* (**F**), *Esr1* (**G**) and *Pbsn* (**H**) for all cells. (**I**) Expression in CP10K of *Greb1* in control cells (left) and E_2-treated cells (right). Only the *Pbsn*-positive epithelial cluster is shown (as defined by the rectangle in [**H**]). (**J**) Heatmap of differentially expressed genes following E_2 treatment in *Pbsn*-positive epithelial cluster, by using a threshold of adjusted *p*-value (FDR) inferior to 0.05. Expression is shown as relative expression in Z-score. (**K**) Expression in log scale of counts per 10k (CP10K) + 1 pseudo count for *Esr2* for all cells. (**L**) Gene Set Enrichment Analysis (GSEA)-Pre-rank analysis of MYC targets V1 (left) and fatty acid metabolism (right) pathways, using genes ordered by decreasing $\log_2$ fold-change between E_2-treated cells and control cells in *Pbsn*-positive luminal cluster (as defined by the rectangle in [**H**]). Normalized enrichment score (NES), *p*-values and false discovery rates (FDR, *q*-values) are indicated on each diagram.

Figure S5

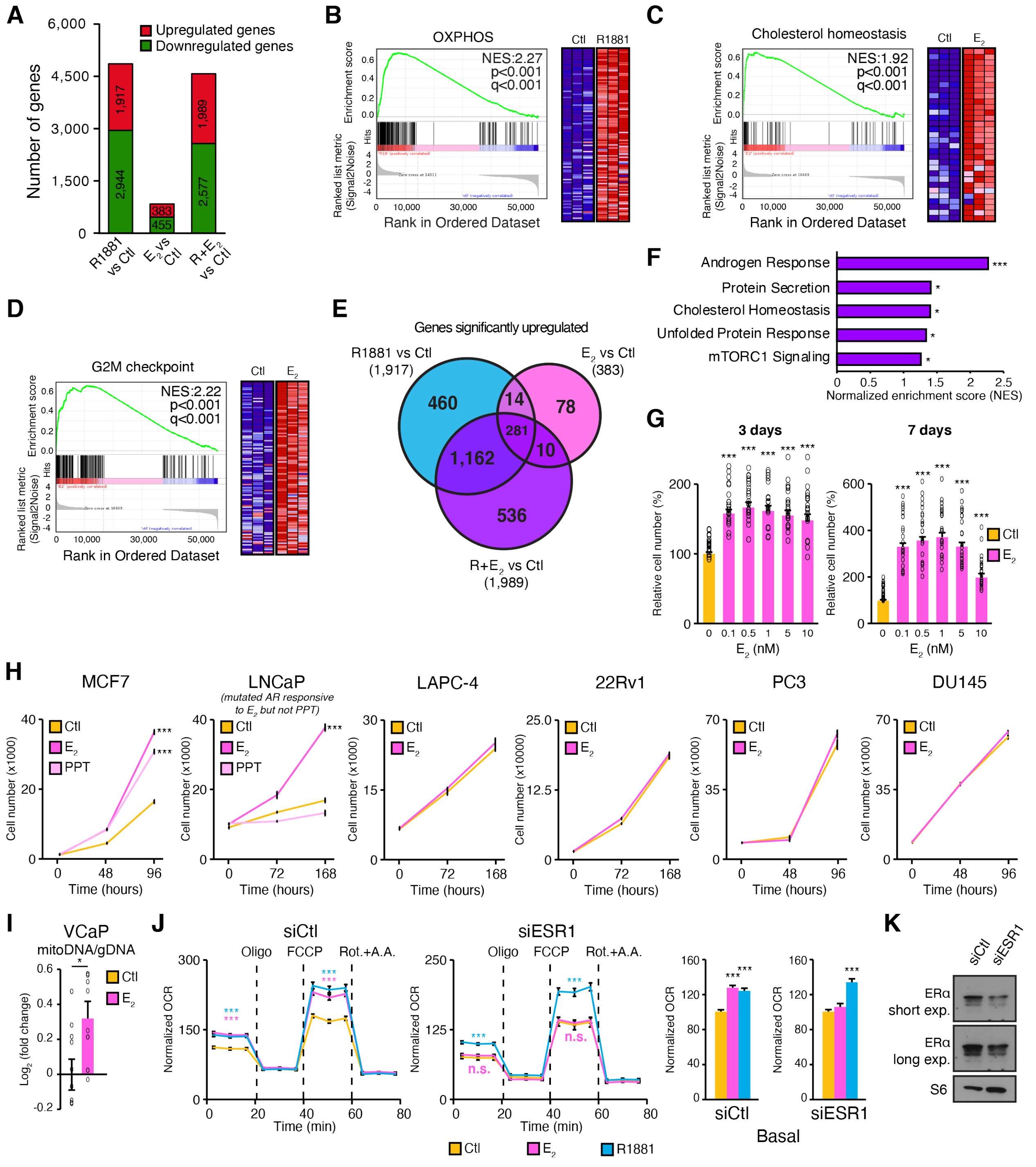

Fig. S5. (**A**) Number of differentially expressed genes following pair-wise comparisons (R1881 *vs* Ctl, E_2 *vs* Ctl, R+E_2 *vs* Ctl) in VCaP cells. The number of up-regulated genes and down-regulated genes for each pair-wise comparison is indicated. Thresholds used were a fold-change ≥ 1.75 and

a significant p value < 0.05 with false-discovery rates (FDR) < 0.1. (**B**) Gene Set Enrichment Analysis (GSEA) diagrams and heatmaps for oxidative phosphorylation (OXPHOS) gene set following treatment with R1881 in VCaP cells. Normalized enrichment score (NES), p-values and false discovery rates (FDR, q-values) are indicated on the diagram. Only core genes of the pathway are shown. (**C**, **D**) GSEA diagrams and heatmaps for cholesterol homeostasis (**C**) and G2M checkpoint (**D**) gene sets following treatment with E_2 in VCaP cells. NES, p-values and q-values are indicated on each diagram. Only core genes of each pathway are shown. (**E**) Venn diagrams of significant up-regulated genes for each pair-wise comparison (R1881 *vs* Ctl, E_2 *vs* Ctl, R+E_2 *vs* Ctl) in VCaP cells. (**F**) NES of GSEA analysis enriched following treatment with R+E_2 compared to control, R1881, and E_2 treatments in VCaP cells. *$p < 0.05$ and ***$p < 0.001$. (**G**) Proliferation assays following E_2 stimulation in a dose-dependent manner in VCaP cells for 3 or 7 days of treatment. Results are shown as the average with standard error of the mean (S.E.M.) of three independent experiments. The changes in cell numbers were normalized in percentages, according to the control (Ctl) treatment set at 100%. ***$p < 0.001$. (**H**) Proliferation assays following E_2 stimulation and the ERα-specific ligand PPT (1 nM to ensure specific ERα activation) for the positive control MCF7 breast cancer cells and in ERα-negative PCa cells LNCaP, LAPC4, 22Rv1, and DU145 cells. Note that LNCaP have a mutated AR that binds E_2, thus mimicking androgen stimulation. PC3 cells have detectable but really low levels of ERα, hence no response to estrogenic stimulation. One representative experiment out of three per cell line is shown as the mean ± S.E.M. (n = 8/treatment group). (**I**) Changes (in $\log_2$ fold-change) of mitochondrial over genomic DNA ratio (mitoDNA/gDNA) following estrogen stimulation in VCaP cells. The mean ± S.E.M. of three independent experiments is represented, with the control group set at 0, n = 3 samples/group treatment. *$p < 0.05$. (**J**) VCaP respiratory profiles following treatment with either R1881, E_2, or

vehicles, with and without knockdown of *ESR1* using siRNAs. Complete mitochondrial stress tests are shown on the left and basal and maximal respiratory capacities are shown on the right. Before measurement of oxygen consumption rates (OCR), VCaP cells were treated with and without hormones for 72h. One representative experiment out of three independent experiments is shown. Results are shown as the mean ± S.E.M. (n = 10–12/treatment group). ***$p < 0.001$. (**K**) siRNAs validation by Western Blot in VCaP cells. Knockdown of ERα expression can be observed in the siESR1 sample as opposed to the siCtl sample. S6 is used as a loading control.

Figure S6

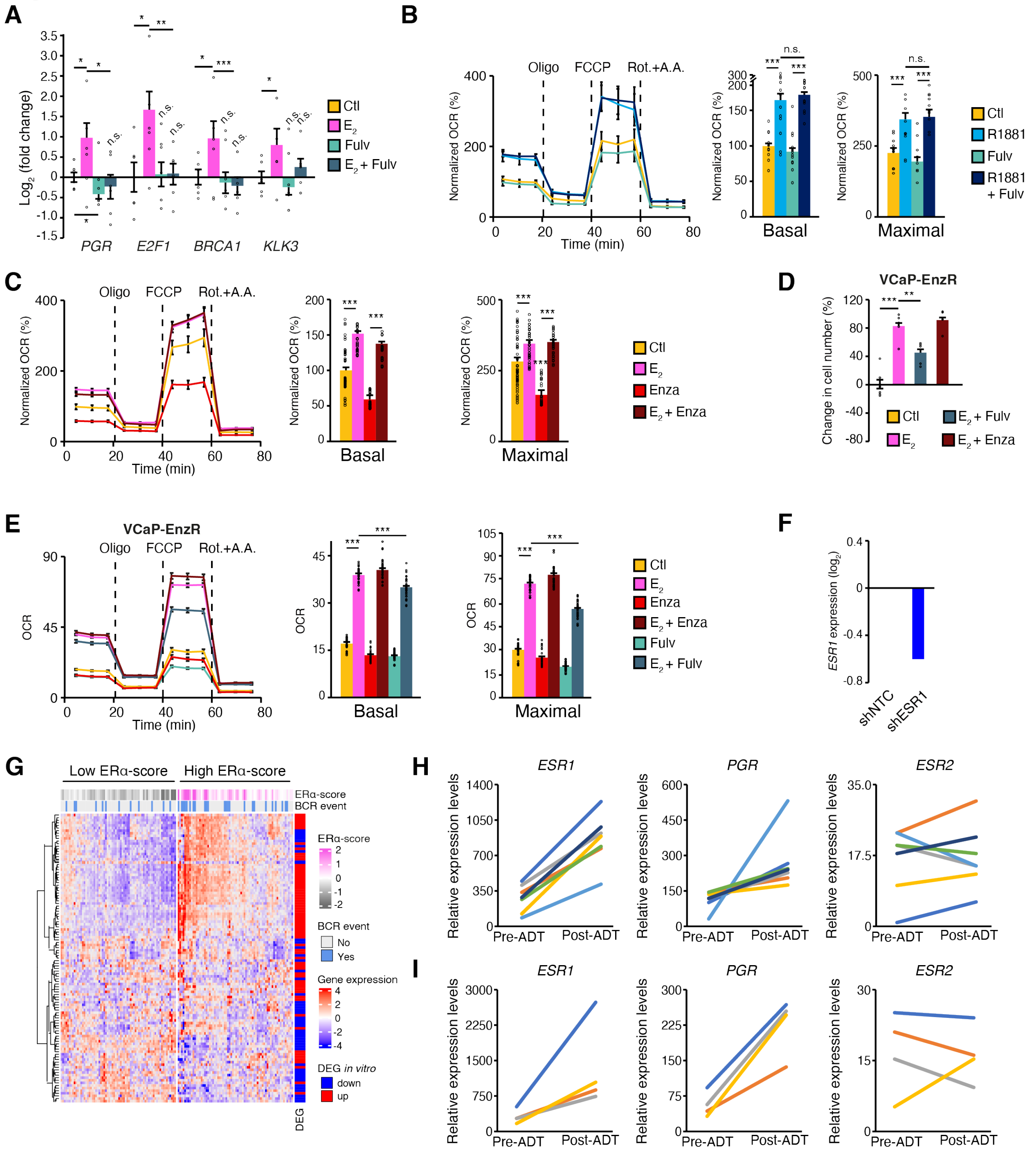

493

494 **Fig. S6.** (**A**) qRT-PCR validation of genes sensitive to estradiol (E_2) stimulation and blockade upon

495 co-treatment with the anti-estrogen fulvestrant (Fulv) in parental VCaP cells. Results are shown as

496 the mean ± standard error of the mean (S.E.M.) of two independent experiments performed in

triplicate biological samples (n = 6/group). (**B**) VCaP respiratory profiles following 72h treatment with R1881 and/or fulvestrant (Fulv). Basal OCR of control treatment was set at 100%. Complete mitochondrial stress tests are shown on the left and basal and maximal respiratory capacities are shown on the right. One representative experiment out of three independent experiments is shown. Results are shown as the mean ± S.E.M. (n = 10–12/treatment group). (**C**) Parental VCaP respiratory profiles following 72h treatment with either E_2, enzalutamide (Enza), both compounds (E_2 + Enza) or vehicles (Ctl). Basal OCR of the control treatment was set at 100%. Complete mitochondrial stress tests are shown on the left and basal and maximal respiratory capacities are shown on the right. One representative experiment out of three independent experiments is shown. Results are shown as the mean ± S.E.M. (n = 10–12/treatment group). (**D**) Proliferation assays following E_2, Fulv, or Enza stimulation in VCaP-EnzR (enzalutamide-resistant) cells. Results are shown as the average and S.E.M. of one out of three independent experiments are shown (n = 8/group). (**E**) VCaP-EnzR respiratory profiles after 72h treatment with and without E_2, Fulv, or Enza. Complete mitochondrial stress tests are shown on the left and basal and maximal respiratory capacities are shown on the right. One representative experiment out of two independent experiments is shown. Results are shown as the mean ± S.E.M. (n = 10–12/treatment group). (**F**) Validation of *ESR1* knockdown by qPCR in the human PDO line #1. *ESR1* levels in organoids transduced with shNTC were set at 0 for comparison (n = 1 due to limited material with PDO). (**G**) Heatmap of ERα gene signature (as defined using RNA-seq data of VCaP cells [Figure 4]) in patients from the Taylor *et al.* dataset (58). Top legend shows ERα-score for each patient (pink = high and gray = low), indicative of the predicated transcriptional activity of ERα in vivo, and biochemical recurrence (BCR), representative of the progression-free interval (PFI) indicator. Right legend shows if the differentially expressed genes (DEG) included in the ERα-signature were

increased (red) or decreased (blue) in vitro following E_2 treatment in VCaP cells. (**H**) *ESR1* (encoding for ERα), *ESR2* (encoding for ERβ) and *PGR* (encoding for the progesterone receptor) relative gene expression in PCa tumors before and after ADT in the Eur Uro 2014 dataset, comprising 7 paired samples. (**I**) *ESR1*, *ESR2* and *PGR* relative gene expression in PCa tumors before and after ADT + docetaxel in the BMC cancer dataset, comprising 4 paired samples. For (**H**, **I**), gene expression changes following treatment is shown by patient (1 patient = 1 color). $*p < 0.05$, $**p < 0.01$ and $***p < 0.001$.

Figure S7

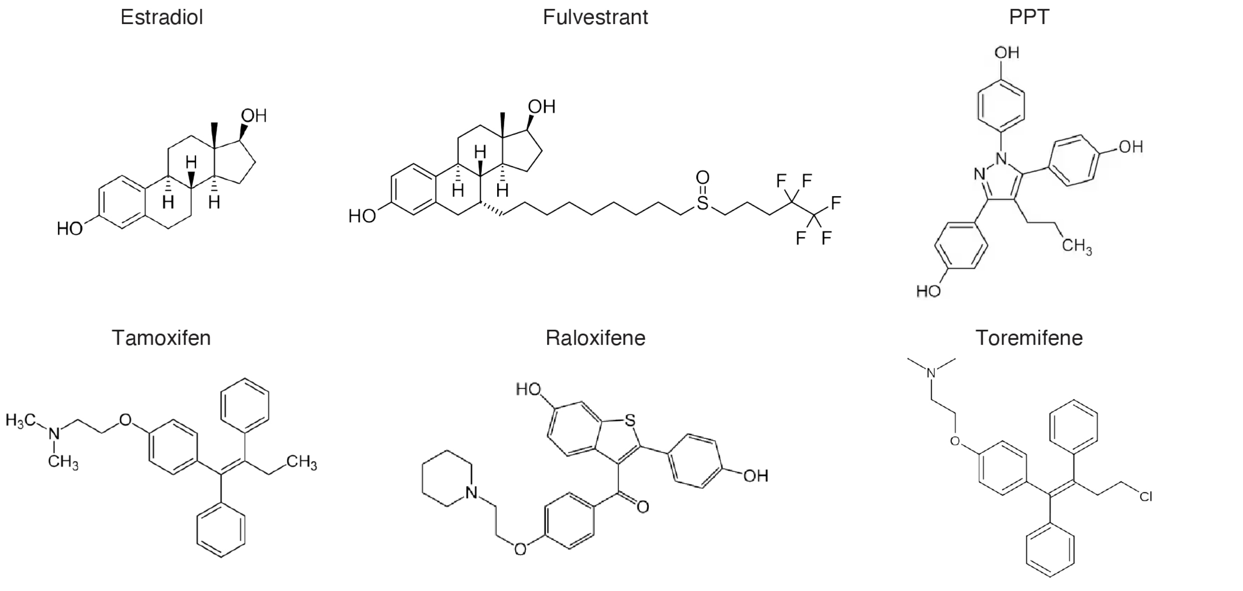

Figure S7. The different ERα ligands used in the current study exhibit distinct molecular structures. The PPT chemical structure was retrieved from the Abcam website.

Supplementary Tables

Supplementary Table 1. Clinical and pathological characteristics of the TMA's discovery cohort (as previously described by Belledant, *et al.* (2)).

**Patients Characteristics**	**Localized PCa (*n* = 239)**
**Age at diagnosis, years**	
Mean	62.9
SD	6.6
Range	43.3–78.4
Follow-up median, months	182
Biochemical recurrence, *n* (%)	73 (31)
Development of metastasis, *n* (%)	15 (6)
**PSA level at diagnosis, *n* (%)**	
≤10 ng/mL	159 (67)
>10–20 ng/mL	62 (26)
>20 ng/mL	17 (7)
**Pathologic Gleason score, *n* (%)**	
≤6	68 (28)
7	124 (52)
≥8	47 (20)
**Pathologic T stage, *n* (%)**	
≤T2c	141 (59)
T3a	63 (26)
≥T3b	35 (15)
**Nodal invasion, *n* (%)**	
N0	232 (97)
N+	7 (3)
**Margin status, *n* (%)**	
Negative	160 (68)
Positive	77 (32)

N: Nodal status.

Supplementary Table 2. Clinical and pathological characteristics of the TMA's validation cohort

Patients Characteristics	PCa with NeoAdj (*n* = 41)
**Age at diagnosis, years**	
Mean	60.5
SD	5.63
Range	44.6–69.3
Follow-up median, years	24.6
Biochemical recurrence, *n* (%)	26 (63)
Development of metastasis, *n* (%)	10 (24)
**PSA level at diagnosis, *n* (%)**	
≤10 ng/mL	14 (34)
>10–20 ng/mL	11 (27)
>20 ng/mL	16 (39)
**Pathologic Gleason score, *n* (%)**	
≤6	12 (29)
7	7 (7)
≥8	22 (54)
**Pathologic T stage, *n* (%)**	
≤T2c	9 (22)
T3a	11 (27)
≥T3b	21 (51)
**Nodal invasion, *n* (%)**	
N0	26 (63)
N+	15 (37)
**Margin status, *n* (%)**	
Negative	20 (49)
Positive	21 (51)
**Metastasic status, *n* (%)**	
Negative	33 (67)
Positive	10 (23)
**Overall survival, *n* (%)**	
Alive	23 (53)
Dead	20 (47)

N: Nodal status.

Note that all patients received neoadjuvant hormonal therapy prior to surgery.

Supplemental Table 6. Quantitative PCR primers of genes in the mouse (*Mus musculus*).

Genes	Sequence (5'→3')	Amplicon size
Atp5o	ATCTGTGGTCAGGCCCTTTG	176
	CCAGAGACACTTTGGGGTCC	
Eef2	CGGGACACGGCTCTTAACAT	165
	CTGCAAGTCTAAGGCAGGCT	
Fkbp11	CGGGCAGTTTGGTAGATGGACGC	184
	GGCGGGTACCCTCGCTTTCC	
Got2	CCTGGTGGACCCATGTTGAA	124
	CCGTTATCATCCCGGTAGGC	
Gstm2	GCCCAAGTGCCTGGATGCCTTC	171
	TTCCCCCAGGTCTGGGCTTTGTA	
Greb1	TGTCCGTCACTTACTGTGCC	268
	TGACTTCAGGCCAAGAGCAG	
Hsd17b10	TTGAGGGCCAGGTATGTGAAC	270
	CTTCTCTCATCTGCCTACAGGTC	
Leng8	TACTTGCCTGTGCCTCACAC	124
	TCACCCACATTGCCTGCTAA	
Mdh1	AAAGACGACAGCTGGCTGAA	105
	TTTCGCAGCAGACATTGCAC	
Mdh2	GACCTGTTCAACACCAACGC	133
	TCCCATGCACTCAAAGAGCC	
Ndufb9	GGCCGCTGAAGGGACAAG	170
	CCCGGAGAGTCTGGGAAGATA	
Ogdh	ACTCGTGGAGGACCACTTG	159
	GCAAGATCAAGTTTGTCTGTGGA	
Pfkfb3	GGGAGAGGTCAGAGAACATGAA	268
	TGCTGCTCTACACAGGAAGC	
Pgr	GCTTGGACTCAGGTCCCTTC	222
	GGGCTCTGGAATTTCTGCCT	
Pum1	AACATCGATGGCCTACAGGG	267
	GACCAGGTCTTCTCTGCACC	
Sc5d	GACAGCAGCTACACCTTCACT	268
	CTGGAAGCCCTGAGATCCAA	
Tbp	GTTGGGCTTCCCAGCTAAGT	94
	CACAAGGCCTTCCAGCCTTA	

For each targeted gene, amplicons were validated with Sanger sequencing.

Supplementary Table 7. Quantitative PCR primers of genes in human (*Homo sapiens*).

Genes	Sequence (5’→3’)	Amplicon size
ACTN	ACATGCAGCCAGAAGAGGAC	89
	ACACCATGCCGTGAATGTCT	
BRCA1	ACTACCCATTTTCCTCCCGC	213
	GGACAGCACTTCCTGATTTTGT	
CYTB	TCTCCGATCCGTCCCTAACA	130
	TGATTGGCTTAGTGGGCGAA	
E2F1	GCTCAAAAGTACCCGGACCA	127
	CCGTGGTGATGAGCAGTTCT	
EP300	AATGAATTGGCGAGAGGTTG	201
	AAACCTCACTCCTGCCACAC	
ESR1	TGGGAATGATGAAAGGTGGGAT	129
	GGTTGGCAGCTCTCATGTCT	
HPCAL4	TTTGGGTGAGGACAGAGGTC	212
	CCAGCCACAAAGAAGATGGT	
KLK3	CTGCTCGTGGGTCATTCTGA	186
	TAGACAGGTCGGTGGGACAA	
ND1	CCCTAAAACCCGCCACATCT	69
	GAGCGATGGTGAGAGCTAAGGT	
PGR	ATCTGCCCACTGACGTGTTT	212
	ACCCTACGCATACCTATTTTCAGAT	
PUM1	ACGGATTCGAGGCCACGTCC	104
	CATTAATTACCTGCTGGTCTGAAGGA	
TBP	TGCCACGCCAGCTTCGGAGA	147
	ACCGCAGCAAACCGCTTGGG	

For each targeted gene, amplicons were validated with Sanger sequencing.

Supplementary Table 8. Sequence of the human shESR1.

shRNA	Sequence (5’→3’)
shESR1	CTAGCCTACAGGCCAAATTCAGATAATACTAGTTTATCTGAATTTGGCCTGTAGTTTTTG
	AATTCAAAAACTACAGGCCAAATTCAGATAAACTAGTATTATCTGAATTTGGCCTGTAGG

References

1. Wang SY, et al. Prostate-specific deletion of the murine Pten tumor suppressor gene leads to metastatic prostate cancer. *Cancer Cell.* 2003;4(3):209-21.
2. Belledant A, et al. The UGT2B28 Sex-steroid Inactivation Pathway Is a Regulator of Steroidogenesis and Modifies the Risk of Prostate Cancer Progression. *European Urology.* 2016;69(4):601-9.
3. Lacombe L, et al. UGT2B28 accelerates prostate cancer progression through stabilization of the endocytic adaptor protein HIP1 regulating AR and EGFR pathways. *Cancer Letters.* 2023;553:215994.
4. Audet-Walsh E, et al. Androgen-Dependent Repression of ERR gamma Reprograms Metabolism in Prostate Cancer. *Cancer Research.* 2017;77(2):378-89.
5. Campolina-Silva GH, et al. Reduced vitamin D receptor (VDR) expression and plasma vitamin D levels are associated with aging-related prostate lesions. *Prostate.* 2018;78(7):532-46.
6. Morais-Santos M, et al. Changes in Estrogen Receptor ERbeta (ESR2) Expression without Changes in the Estradiol Levels in the Prostate of Aging Rats. *PLoS One.* 2015;10(7):e0131901.
7. Andrews S. FastQC - A quality control tool for high throughput sequence data. https://www.bioinformatics.babraham.ac.uk/projects/fastqc/.
8. Ewels P, et al. MultiQC: summarize analysis results for multiple tools and samples in a single report. *Bioinformatics.* 2016;32(19):3047-8 %U https://doi.org/10.1093/bioinformatics/btw354.
9. Krueger F. Trim Galore - A wrapper tool around Cutadapt and FastQC to consistently apply quality and adapter trimming to FastQ files. https://www.bioinformatics.babraham.ac.uk/projects/trim_galore/.
10. Martin M. Cutadapt removes adapter sequences from high-throughput sequencing reads. *EMBnetjournal.* 2011;17(1):10-2 %* Copyright (c) %U http://journal.embnet.org/index.php/embnetjournal/article/view/200.
11. Bray NL, et al. Near-optimal probabilistic RNA-seq quantification. *Nature Biotechnology.* 2016;34(5):525-7.
12. Love MI, et al. Moderated estimation of fold change and dispersion for RNA-seq data with DESeq2. *Genome Biology.* 2014;15(12):550 %U http://genomebiology.biomedcentral.com/articles/10.1186/s13059-014-0550-8.
13. Mootha VK, et al. PGC-1alpha-responsive genes involved in oxidative phosphorylation are coordinately downregulated in human diabetes. *Nature Genetics.* 2003;34(3):267-73.
14. Zhou YY, et al. Metascape provides a biologist-oriented resource for the analysis of systems-level datasets. *Nature Communications.* 2019;10.
15. Frégeau-Proulx L, et al. FACS-Free isolation and purification protocol of mouse prostate epithelial cells for organoid primary culture. *MethodsX.* 2022;9:101843.
16. Frégeau-Proulx L, et al. Multiple metabolic pathways fuel the truncated tricarboxylic acid cycle of the prostate to sustain constant citrate production and secretion. *Molecular Metabolism.* 2022;62:101516.
17. Di Tommaso P, et al. Nextflow enables reproducible computational workflows. *Nat Biotechnol.* 2017;35(4):316-9.

18. Ewels PA, et al. The nf-core framework for community-curated bioinformatics pipelines. *Nat Biotechnol.* 2020;38(3):276-8.
19. Peltzer A, et al. nf-core/scrnaseq: nf-core/scrnaseq v2.3.2 "Sepia Samarium Salmon" (2.3.2). Zenodo. https://doi.org/10.5281/zenodo.8015242. 2023.
20. Hao Y, et al. Integrated analysis of multimodal single-cell data. *Cell.* 2021;184(13):3573-87 e29.
21. Karthaus WR, et al. Regenerative potential of prostate luminal cells revealed by single-cell analysis. *Science.* 2020;368(6490):497-505.
22. Tiwari R, et al. Variability in testosterone measurement between radioimmunoassay (RIA), chemiluminescence assay (CLIA) and liquid chromatography-tandem mass spectrometry (MS) among prostate cancer patients on androgen deprivation therapy (ADT). *Urol Oncol.* 2022;40(5):193 e15- e20.
23. Taylor BS, et al. Integrative genomic profiling of human prostate cancer. *Cancer Cell.* 2010;18(1):11-22.
24. Loehr J, et al. A Nutrient-Based Cellular Model to Characterize Acetylation-Dependent Protein-Protein Interactions. *Frontiers in Molecular Biosciences.* 2022;9.
25. Pelletier M, et al. Extracellular Flux Analysis to Monitor Glycolytic Rates and Mitochondrial Oxygen Consumption. *Conceptual Background and Bioenergetic/Mitochondrial Aspects of Oncometabolism.* 2014;542:125-49.
26. Lacouture A, et al. A FACS-Free Purification Method to Study Estrogen Signaling, Organoid Formation, and Metabolic Reprogramming in Mammary Epithelial Cells. *Frontiers in Endocrinology.* 2021;12.
27. Gonthier K, et al. Isocitrate dehydrogenase 1 sustains a hybrid cytoplasmic-mitochondrial tricarboxylic acid cycle that can be targeted for therapeutic purposes in prostate cancer. *Molecular Oncology.* 2023;17(10):2109-25.
28. Lu G, et al. The myeloma drug lenalidomide promotes the cereblon-dependent destruction of Ikaros proteins. *Science.* 2014;343(6168):305-9.
29. Lafront C, et al. A Systematic Study of the Impact of Estrogens and Selective Estrogen Receptor Modulators on Prostate Cancer Cell Proliferation. *Scientific Reports.* 2020;10(1):4024.
30. Cancer Genome Atlas Research N. The Molecular Taxonomy of Primary Prostate Cancer. *Cell.* 2015;163(4):1011-25.
31. Gao J, et al. Integrative analysis of complex cancer genomics and clinical profiles using the cBioPortal. *Sci Signal.* 2013;6(269):pl1.
32. Cerami E, et al. The cBio cancer genomics portal: an open platform for exploring multidimensional cancer genomics data. *Cancer Discov.* 2012;2(5):401-4.
33. Boibessot C, and Toren P. Sex steroids in the tumor microenvironment and prostate cancer progression. *Endocr Relat Cancer.* 2018;25(3):R179-R96.
34. Cooke PS, et al. Estrogen receptor expression in developing epididymis, efferent ductules, and other male reproductive organs. *Endocrinology.* 1991;128(6):2874-9.
35. Gevaert T, et al. The potential of tumour microenvironment markers to stratify the risk of recurrence in prostate cancer patients. *PLoS One.* 2020;15(12):e0244663.
36. Gangkak G, et al. Immunohistochemical analysis of estrogen receptors in prostate and clinical correlation in men with benign prostatic hyperplasia. *Investig Clin Urol.* 2017;58(2):117-26.
37. Sehgal PD, et al. Tissue-specific quantification and localization of androgen and estrogen receptors in prostate cancer. *Hum Pathol.* 2019;89:99-108.

38. Lau KM, et al. Rat estrogen receptor-alpha and -beta, and progesterone receptor mRNA expression in various prostatic lobes and microdissected normal and dysplastic epithelial tissues of the Noble rats. *Endocrinology.* 1998;139(1):424-7.
39. Schulze H, and Barrack ER. IMMUNOCYTOCHEMICAL LOCALIZATION OF ESTROGEN-RECEPTORS IN THE NORMAL-MALE AND FEMALE CANINE URINARY-TRACT AND PROSTATE. *Endocrinology.* 1987;121(5):1773-83.
40. Lau KM, and To KF. Importance of Estrogenic Signaling and Its Mediated Receptors in Prostate Cancer. *International Journal of Molecular Sciences.* 2016;17(9).
41. Prins GS, and Birch L. Neonatal Estrogen exposure up-regulates estrogen receptor expression in the developing and adult rat prostate lobes. *Endocrinology.* 1997;138(5):1801-9.
42. Vitkus S, et al. Distinct function of estrogen receptor alpha in smooth muscle and fibroblast cells in prostate development. *Mol Endocrinol.* 2013;27(1):38-49.
43. Chen M, et al. Reduced prostate branching morphogenesis in stromal fibroblast, but not in epithelial, estrogen receptor alpha knockout mice. *Asian J Androl.* 2012;14(4):546-55.
44. Prins GS, et al. Estrogen imprinting of the developing prostate gland is mediated through stromal estrogen receptor alpha: Studies with alpha ERKO and beta ERKO mice. *Cancer Research.* 2001;61(16):6089-97.
45. Bonkhoff H, et al. Estrogen receptor expression in prostate cancer and premalignant prostatic lesions. *Am J Pathol.* 1999;155(2):641-7.
46. Risbridger G, et al. Evidence that epithelial and mesenchymal estrogen receptor-alpha mediates effects of estrogen on prostatic epithelium. *Developmental Biology.* 2001;229(2):432-42.
47. Bosland MC. Chapter 2: The Role of Steroid Hormones in Prostate Carcinogenesis. *JNCI Monographs.* 2000;2000(27):39–66.
48. Cunha GR, et al. Role of stromal-epithelial interactions in hormonal responses. *Archives of Histology and Cytology.* 2004;67(5):417-34.
49. Yu L, et al. Estrogen Promotes Prostate Cancer Cell Migration via Paracrine Release of ENO1 from Stromal Cells. *Molecular Endocrinology.* 2012;26(9):1521-30.
50. Haqq C, et al. Ethnic and racial differences in prostate stromal estrogen receptor alpha. *Prostate.* 2005;65(2):101-9.
51. Yeh CR, et al. Estrogen receptor a in cancer associated fibroblasts suppresses prostate cancer invasion via reducing CCL5, IL6 and macrophage infiltration in the tumor microenvironment. *Molecular Cancer.* 2016;15.
52. Slavin S, et al. Estrogen receptor a in cancer-associated fibroblasts suppresses prostate cancer invasion via modulation of thrombospondin 2 and matrix metalloproteinase 3. *Carcinogenesis.* 2014;35(6):1301-9.
53. Zhang ZS, et al. The proliferative effect of estradiol on human prostate stromal cells is mediated through activation of ERK. *Prostate.* 2008;68(5):508-16.
54. Da J, et al. Estrogen Receptor Alpha (ER alpha)-Associated Fibroblasts Promote Cell Growth in Prostate Cancer. *Cell Biochemistry and Biophysics.* 2015;73(3):793-8.
55. Messier TL, et al. Epigenetic and transcriptome responsiveness to ER modulation by tissue selective estrogen complexes in breast epithelial and breast cancer cells. *PLoS One.* 2022;17(7):e0271725.
56. Nishi K, et al. Novel estrogen-responsive genes (ERGs) for the evaluation of estrogenic activity. *Plos One.* 2022;17(8).

57. Karthaus WR, et al. Regenerative potential of prostate luminal cells revealed by single-cell analysis. *Science.* 2020;368(6490):497-+.
58. Taylor BS, et al. Integrative Genomic Profiling of Human Prostate Cancer. *Cancer Cell.* 2010;18(1):11-22.
