# Supplementary material for: The estrogen signaling pathway reprograms prostate cancer cell metabolism and supports proliferation and disease progression
Source: J Clin Invest. 2024 Apr 16;134(11):e170809. doi: 10.1172/JCI170809 (PMC11142735; doi:10.1172/JCI170809)

Western blots

Fig. 3B; ERa short

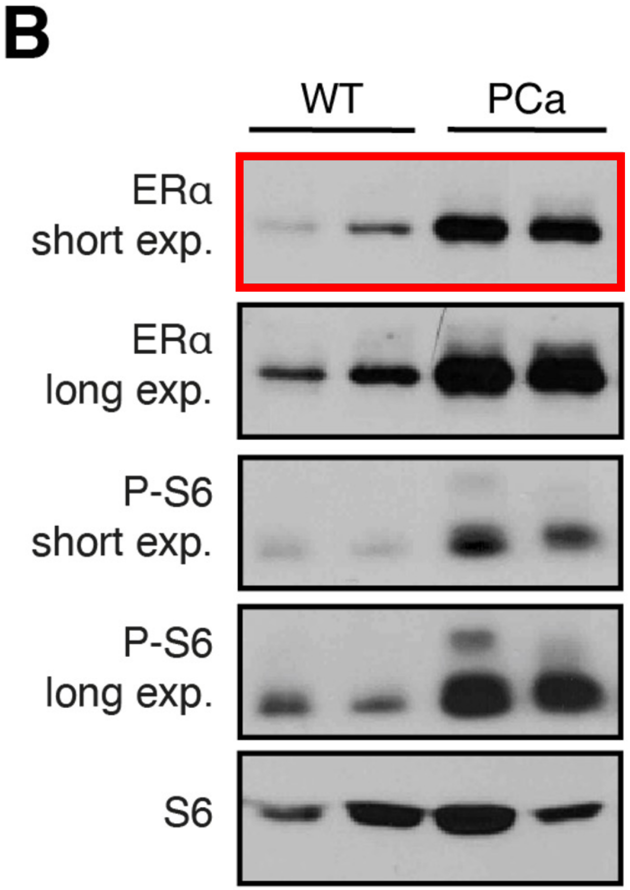

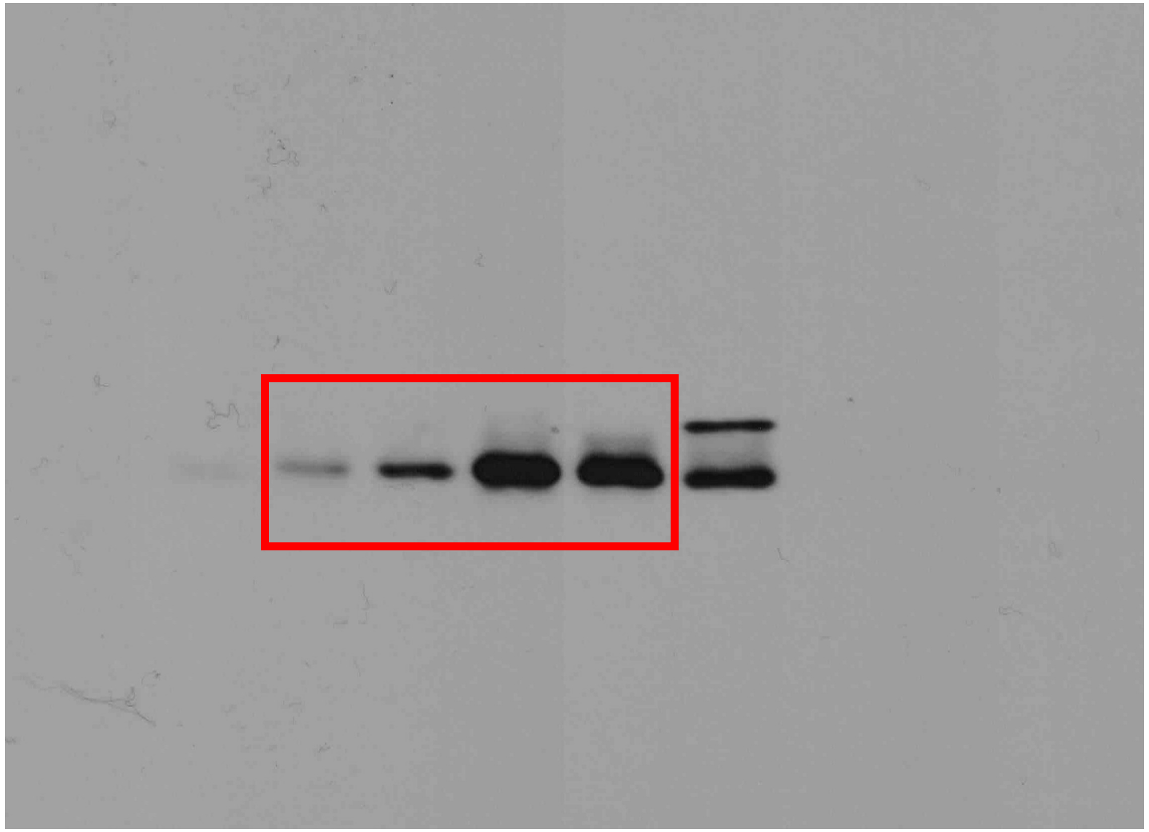

Fig. 3B; ERa long

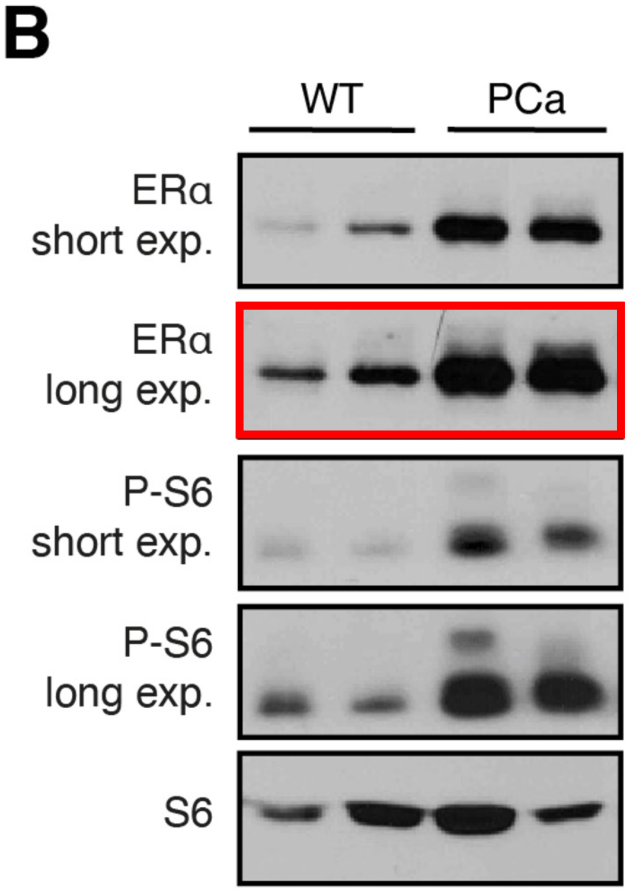

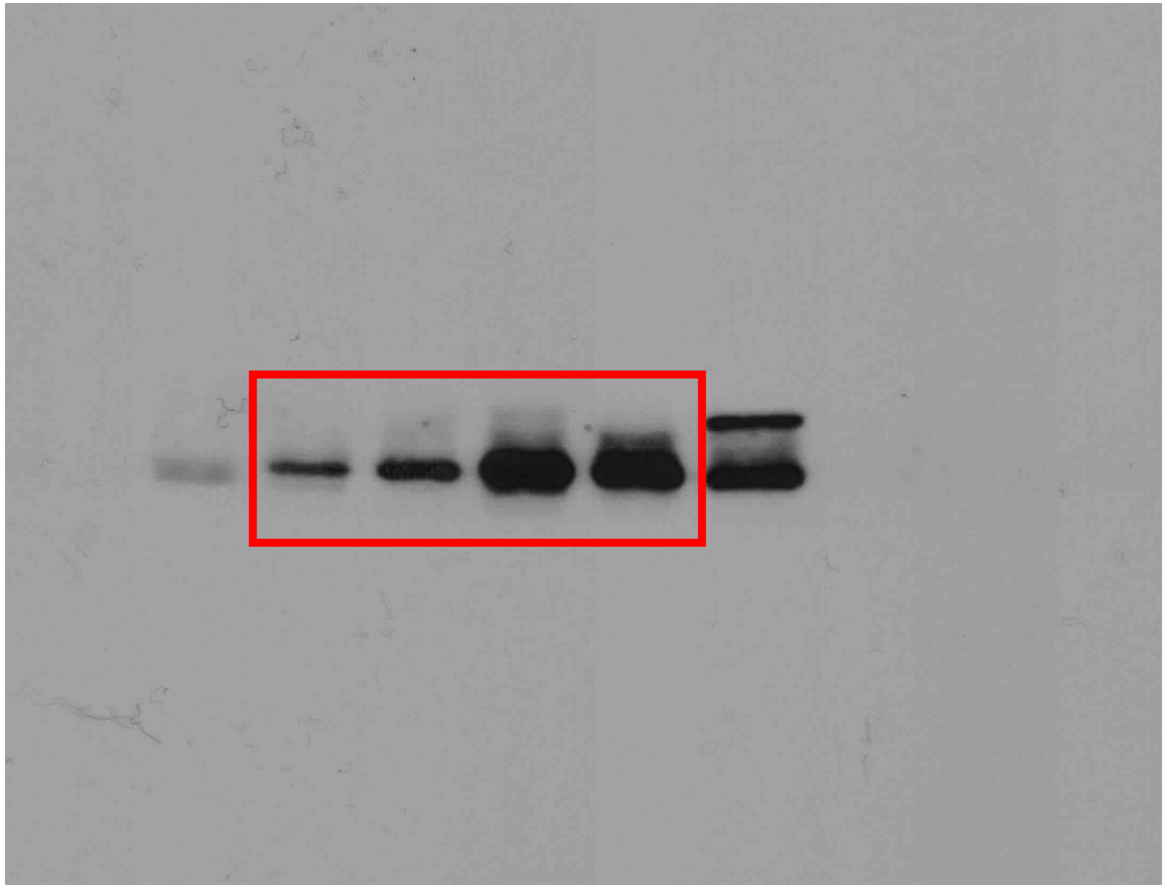

Fig. 3B; P-S6 short

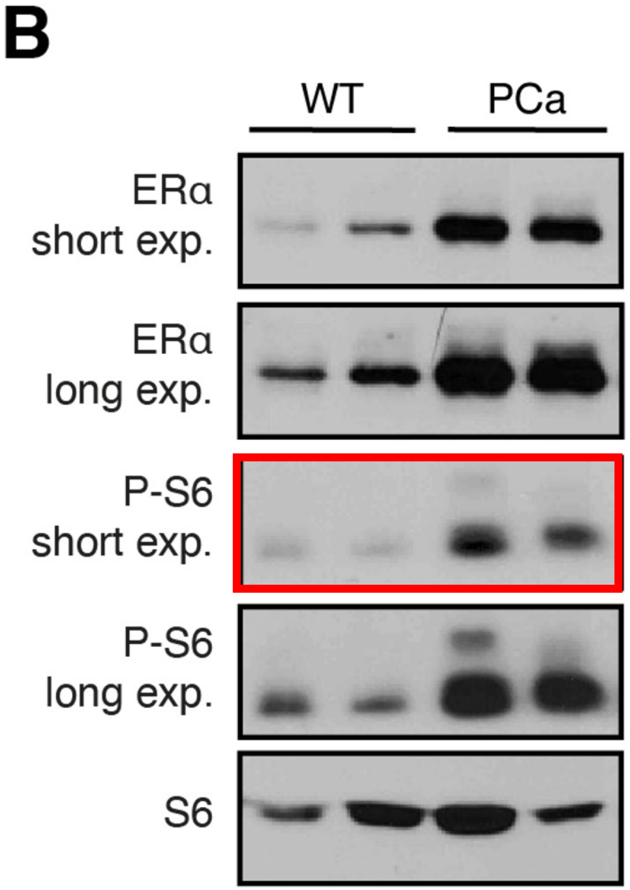

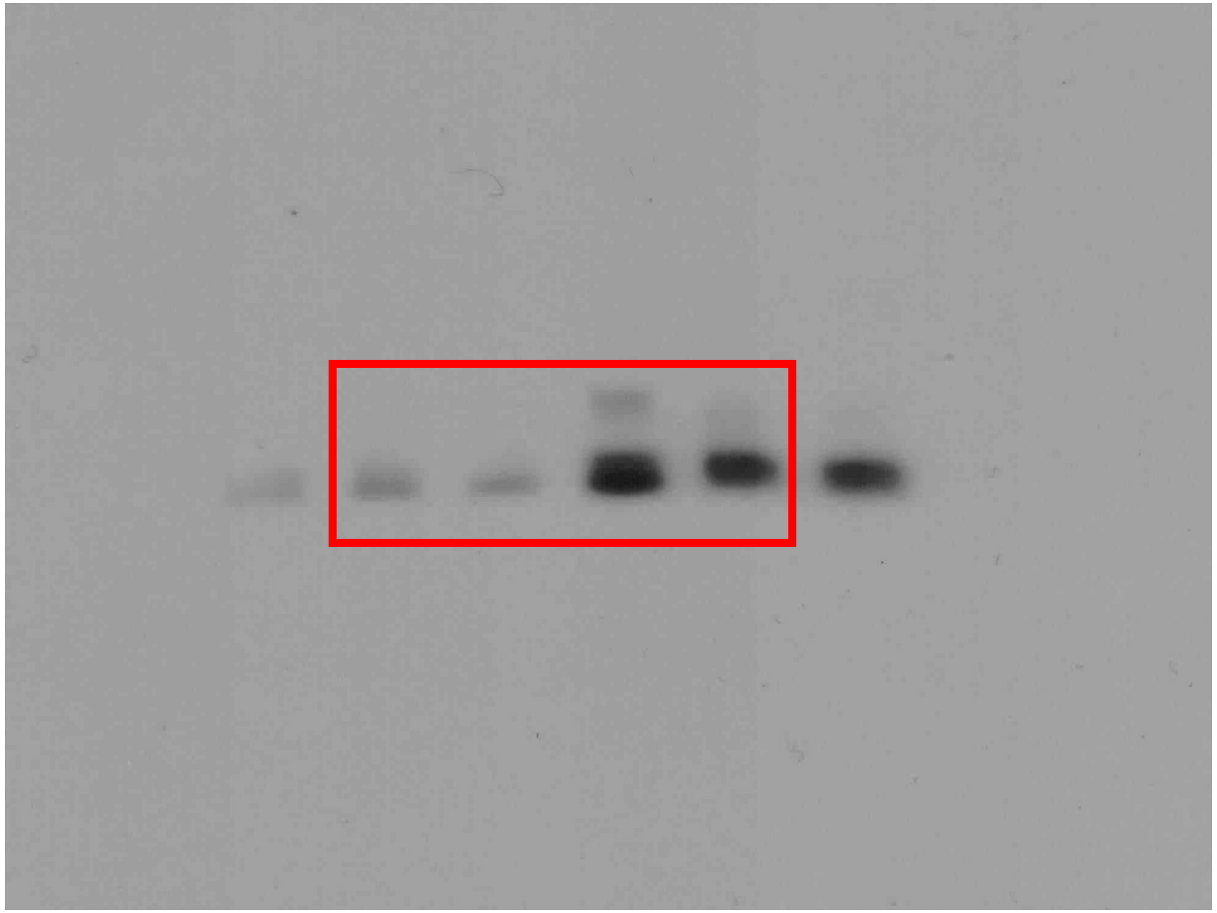

Fig. 3B; P-S6 long

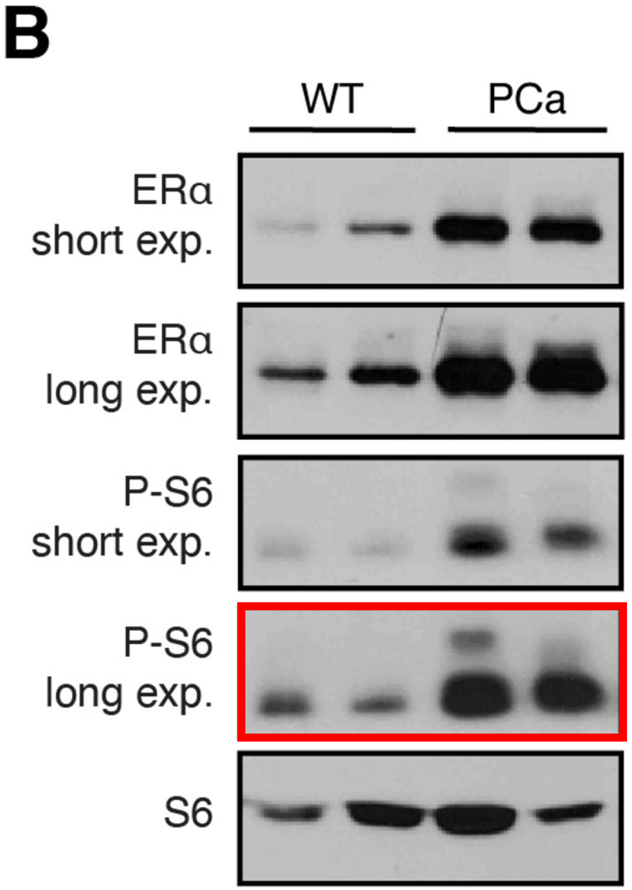

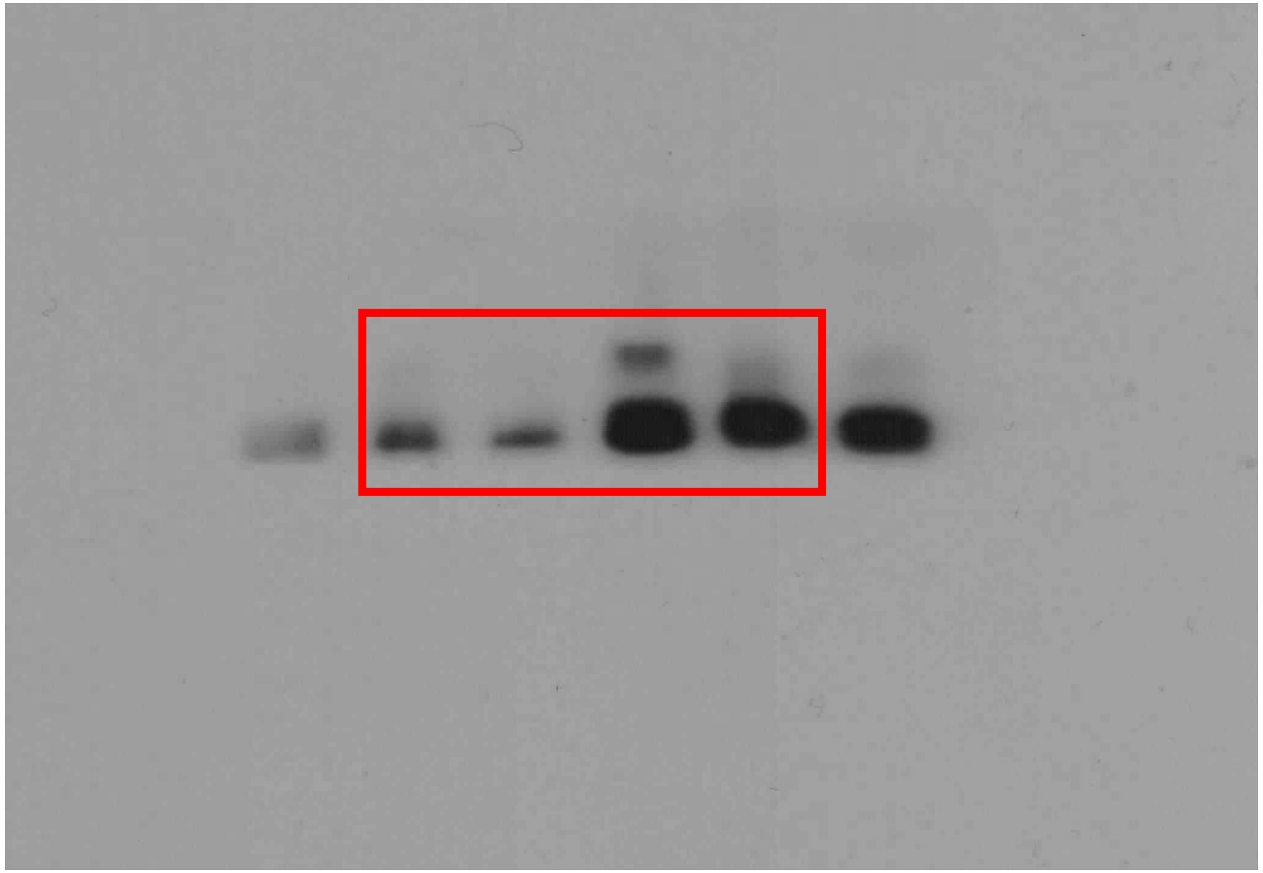

Fig. 3B; S6

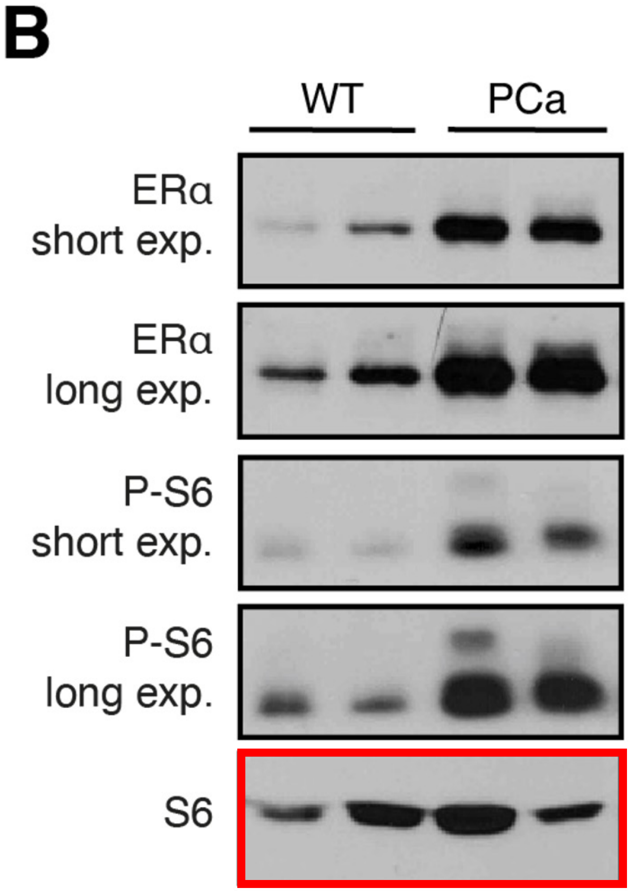
B
WT
PCa
ERα
short exp.
ERα
long exp.
P-S6
short exp.
P-S6
long exp.
S6

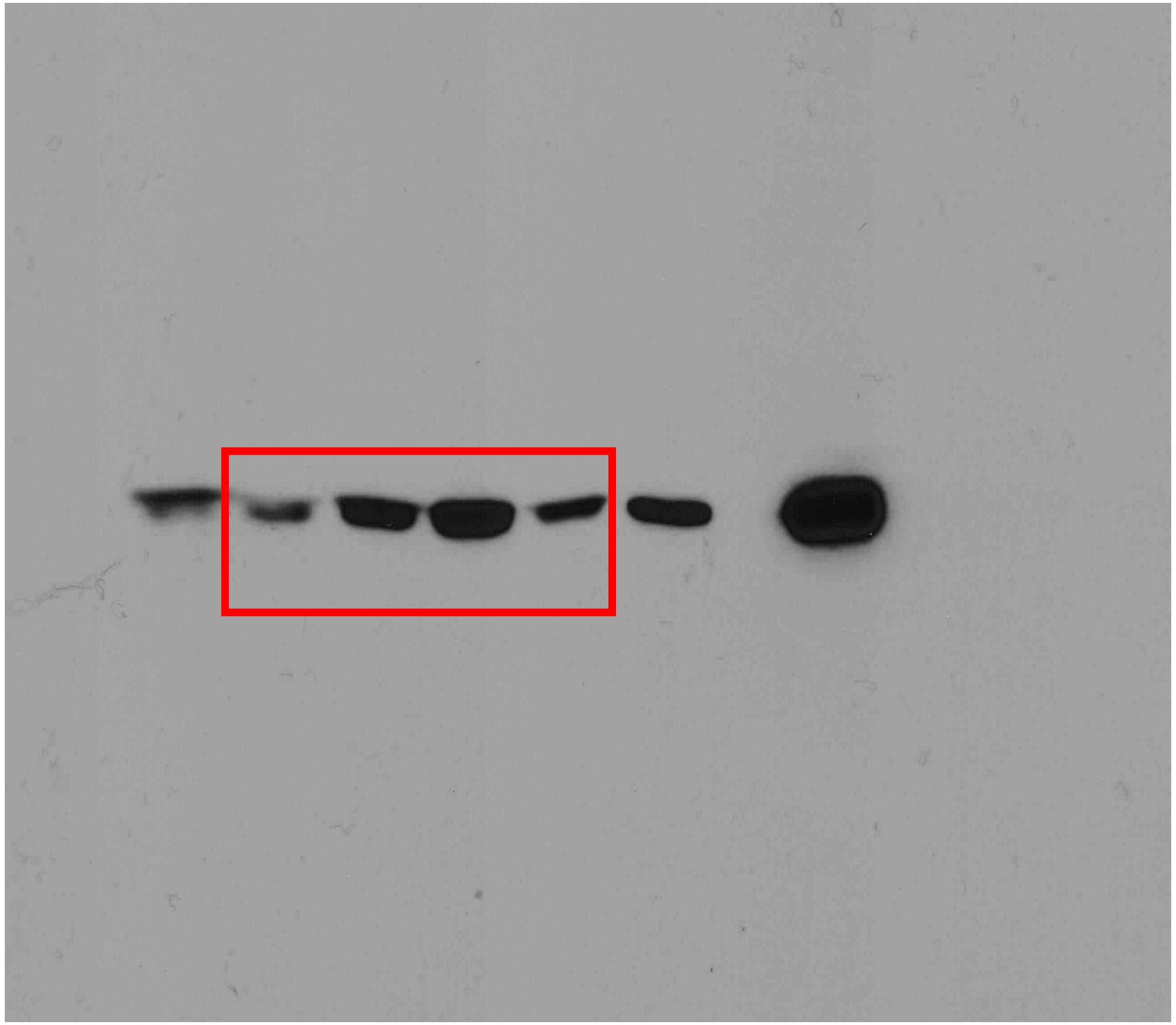

Fig. 4A; AR short

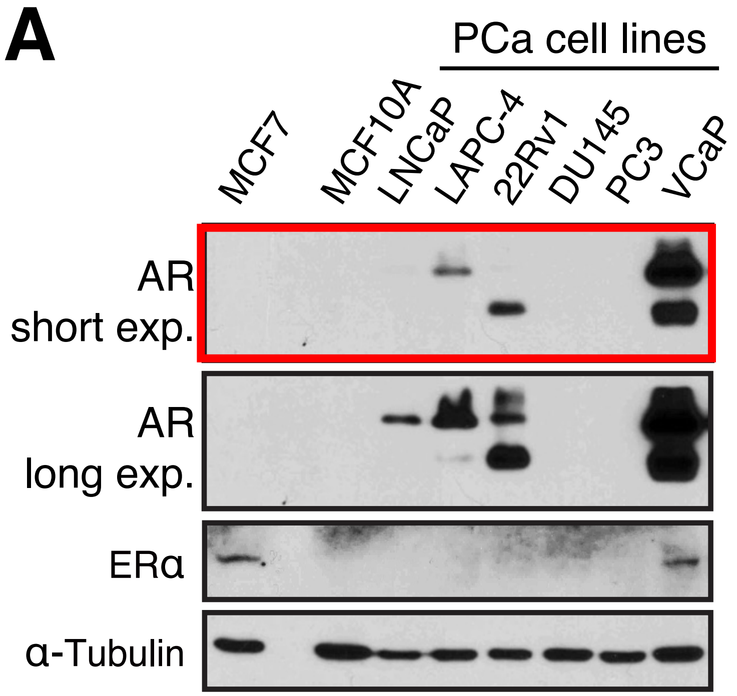

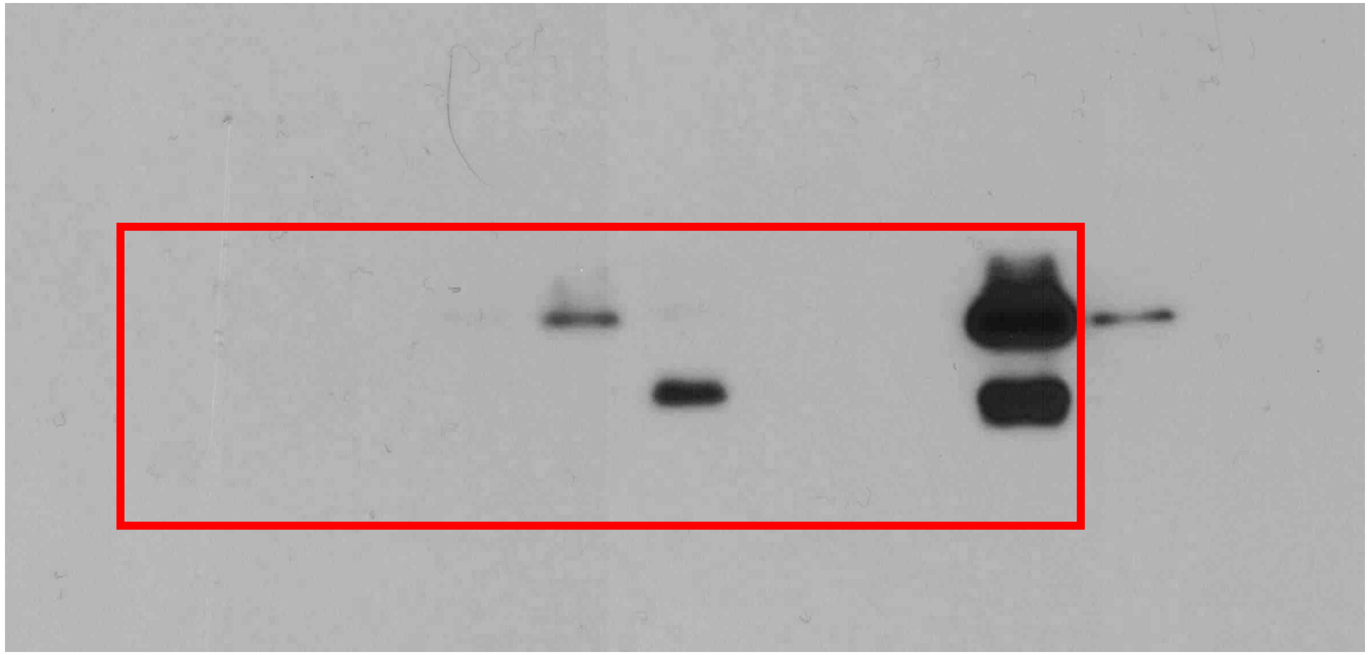

Fig. 4A; AR long

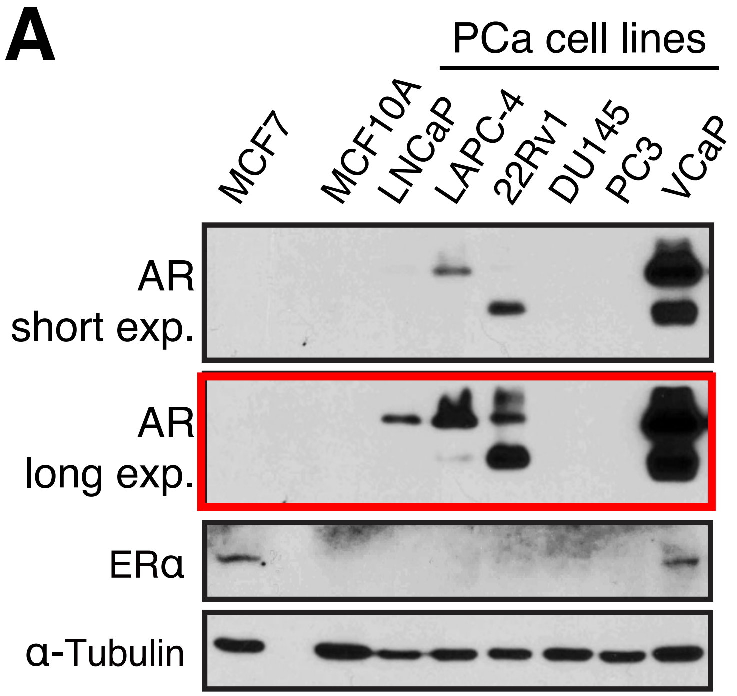

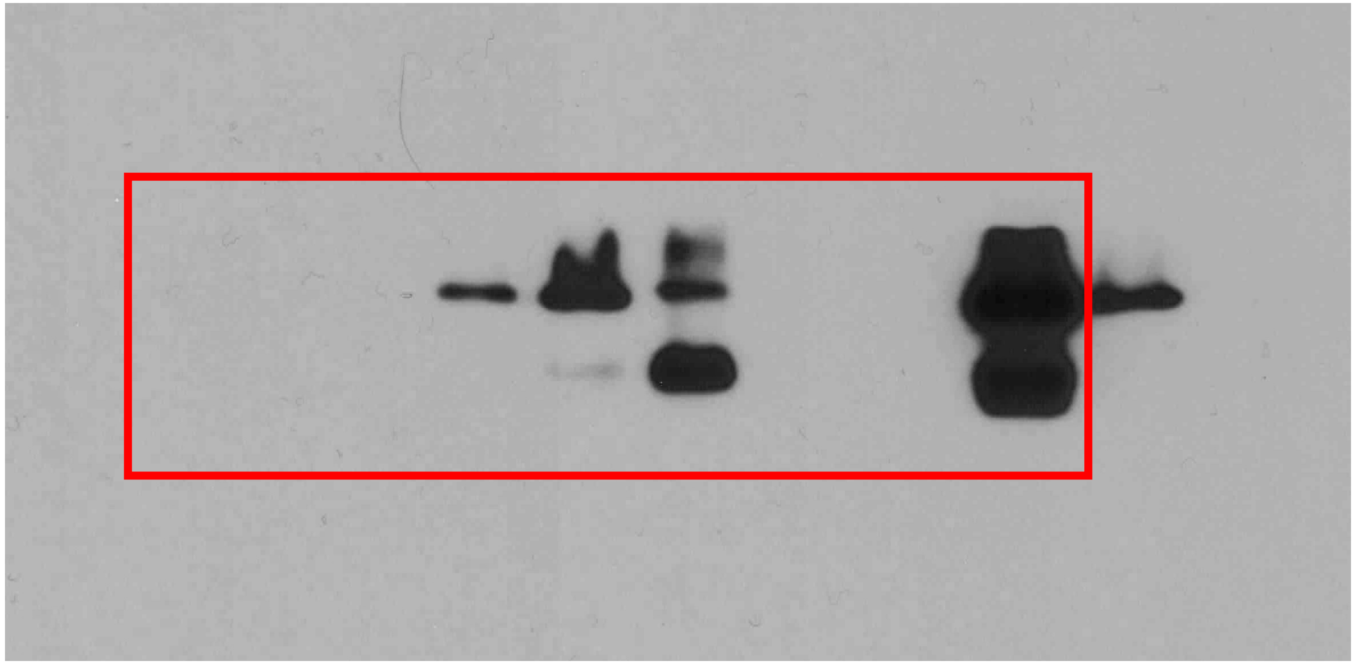

Fig. 4A; ERa

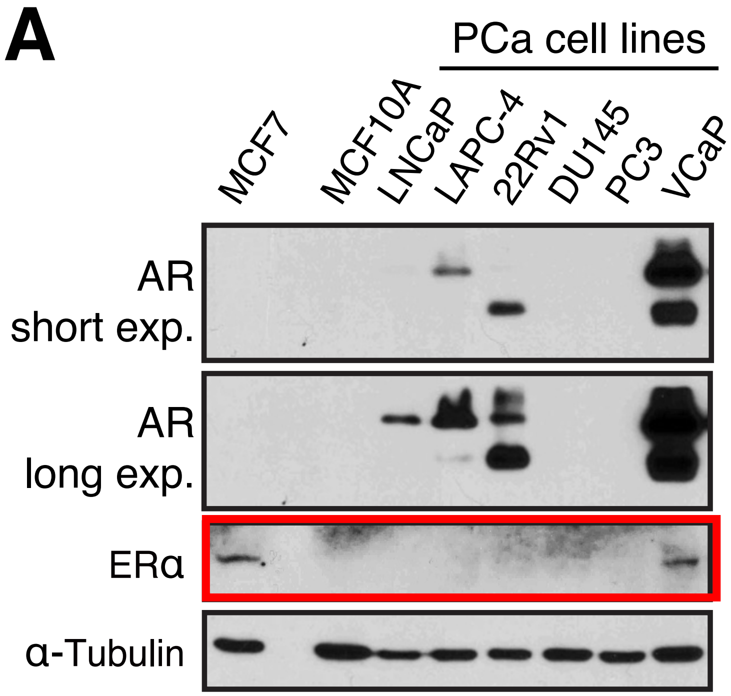
A
PCa cell lines
MCF7
MCF10A
LNCaP
LAPC-4
22Rv1
DU145
PC3
VCaP
AR
short exp.
AR
long exp.
ERα
α-Tubulin

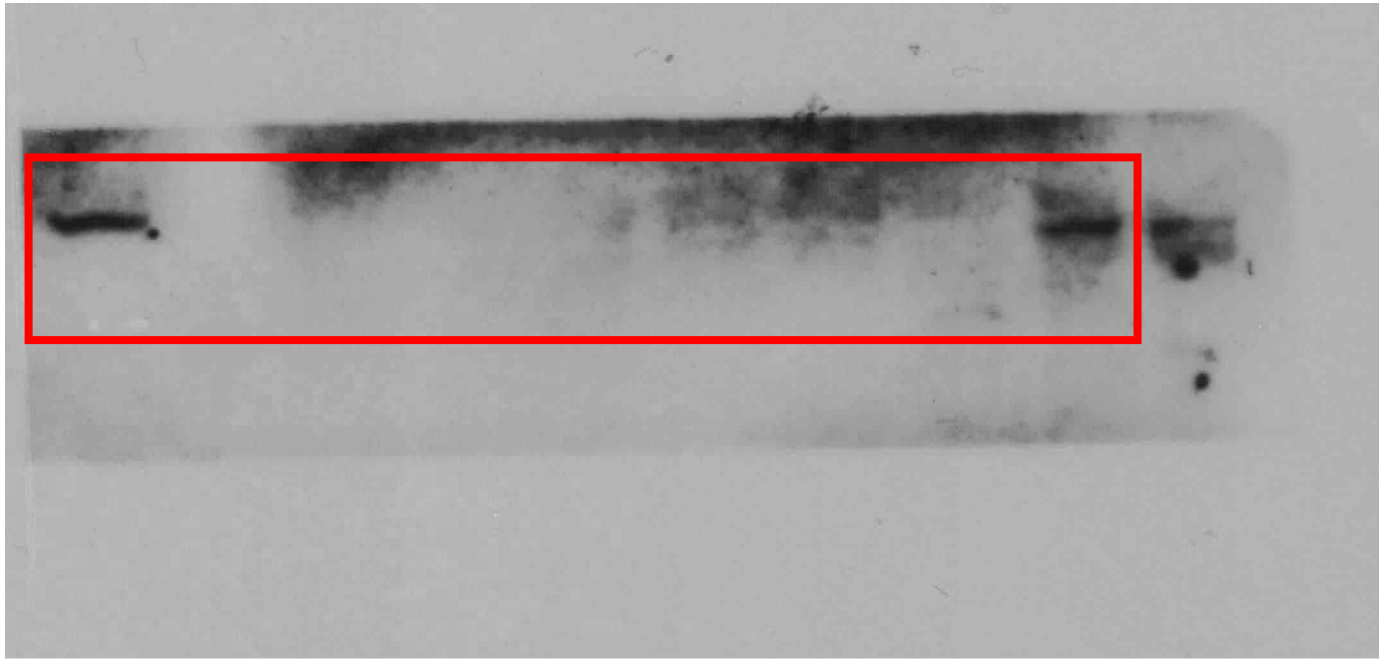

Fig. 4A; a-tubulin

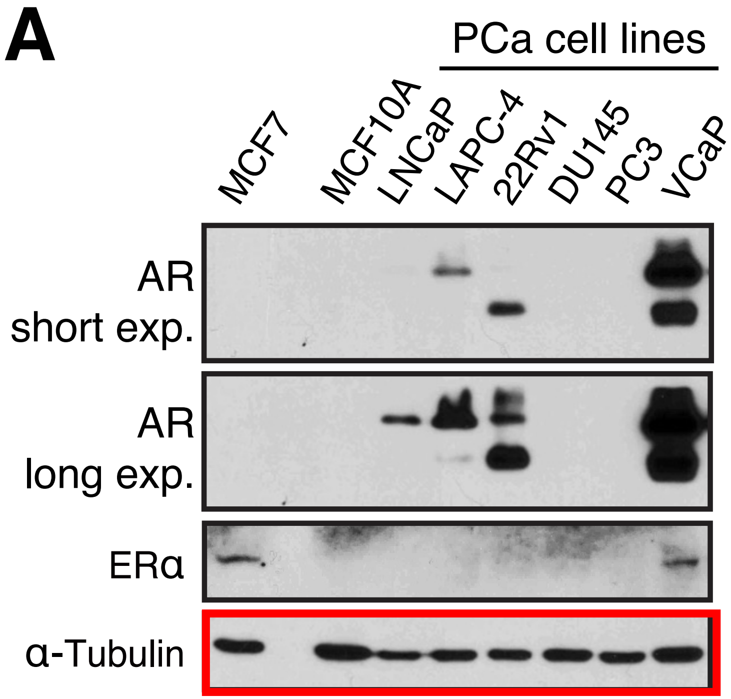

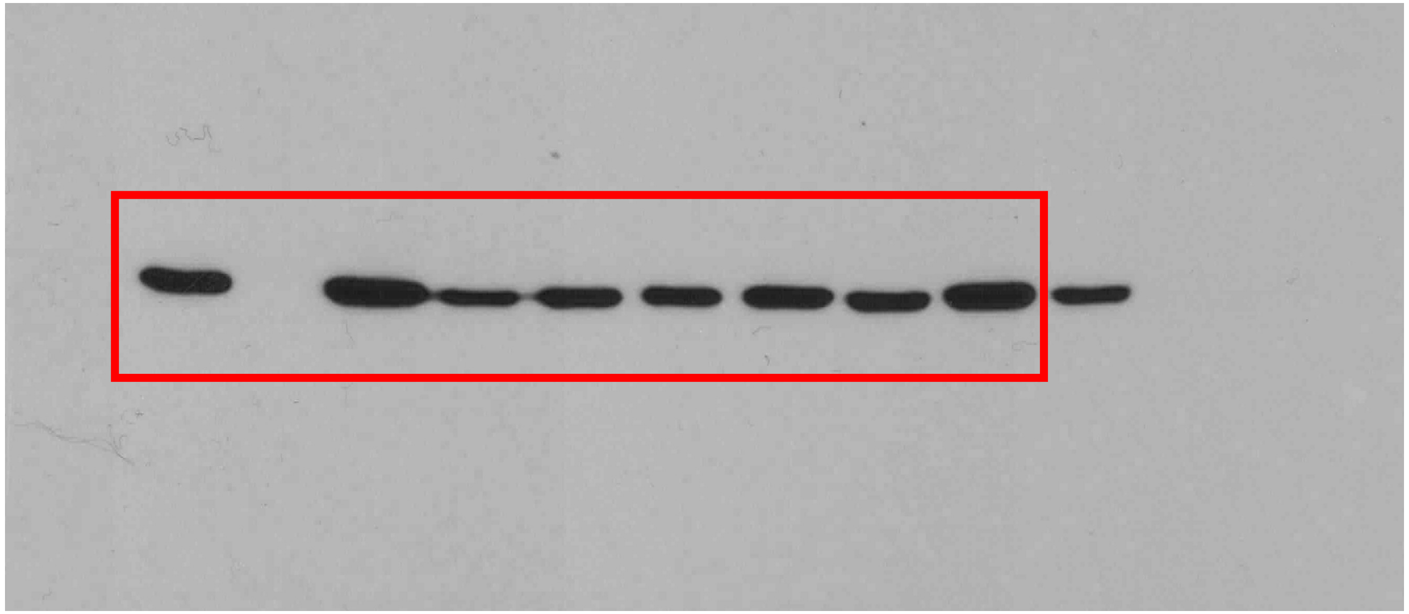

Fig. 5H; P-S6K

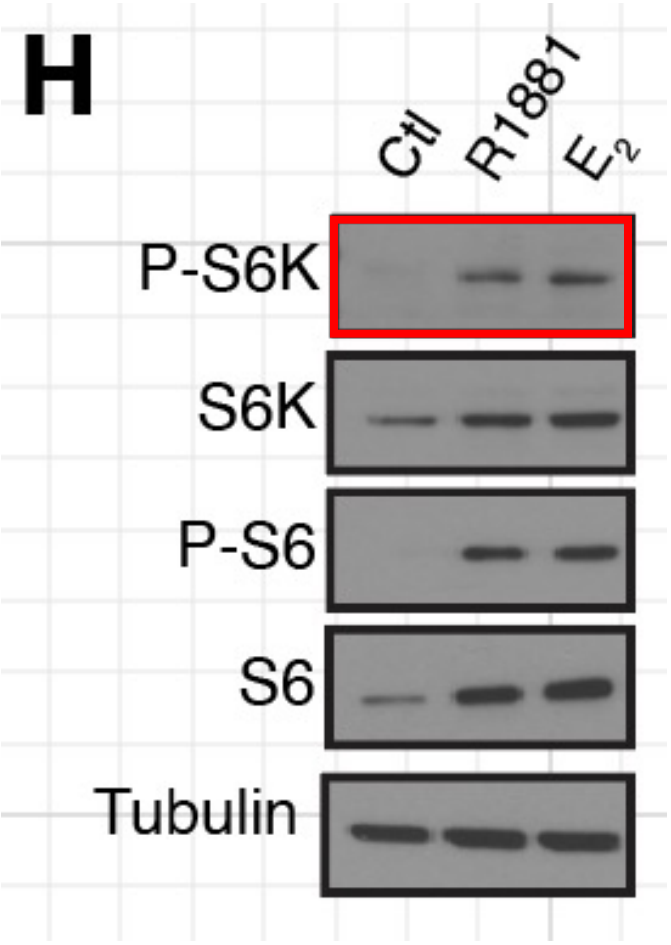

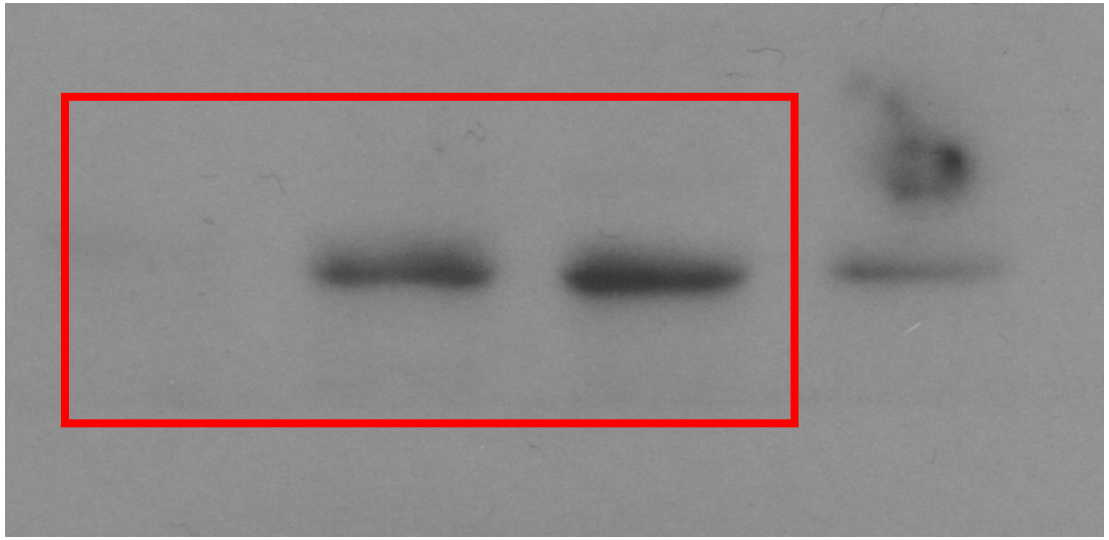

Fig. 5H; S6K

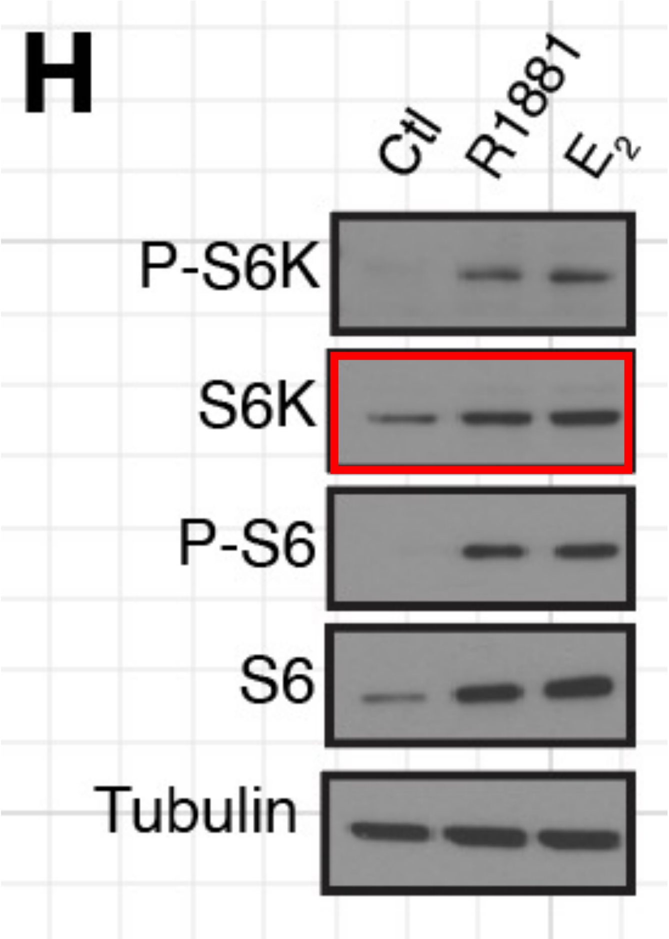

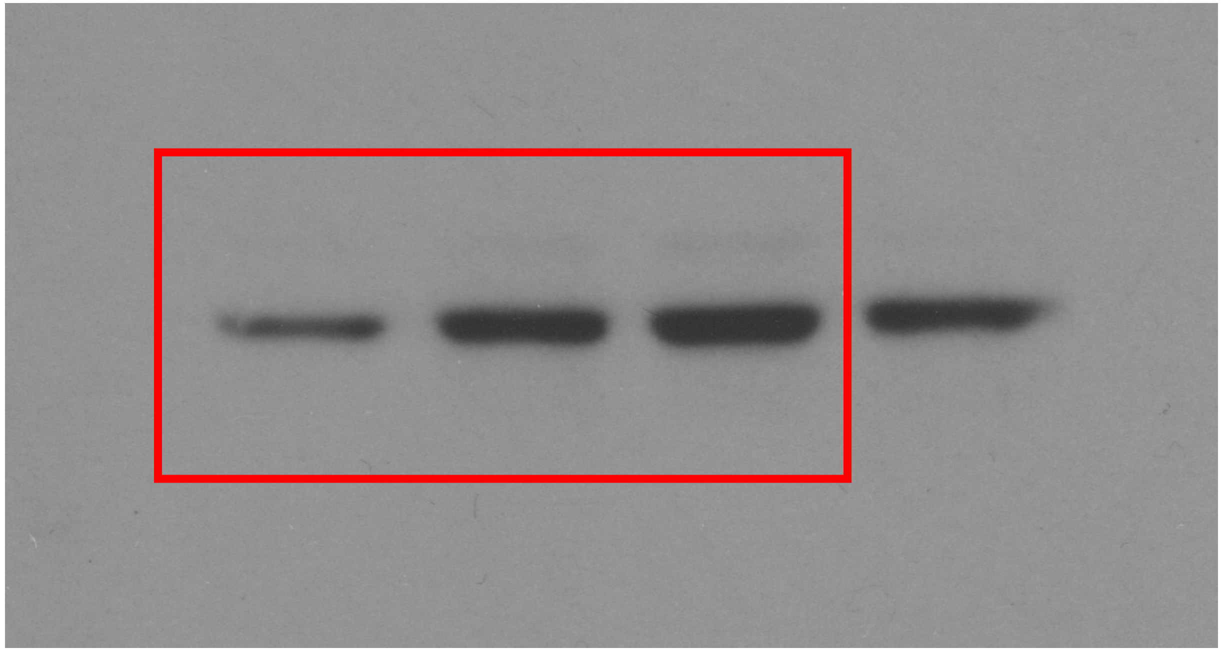

Fig. 5H; P-S6

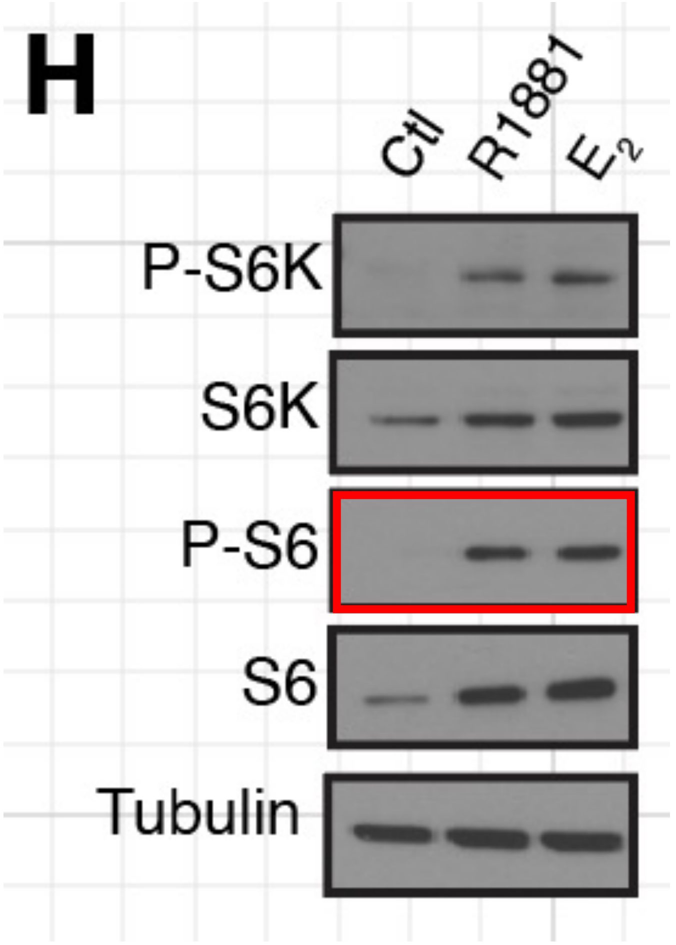

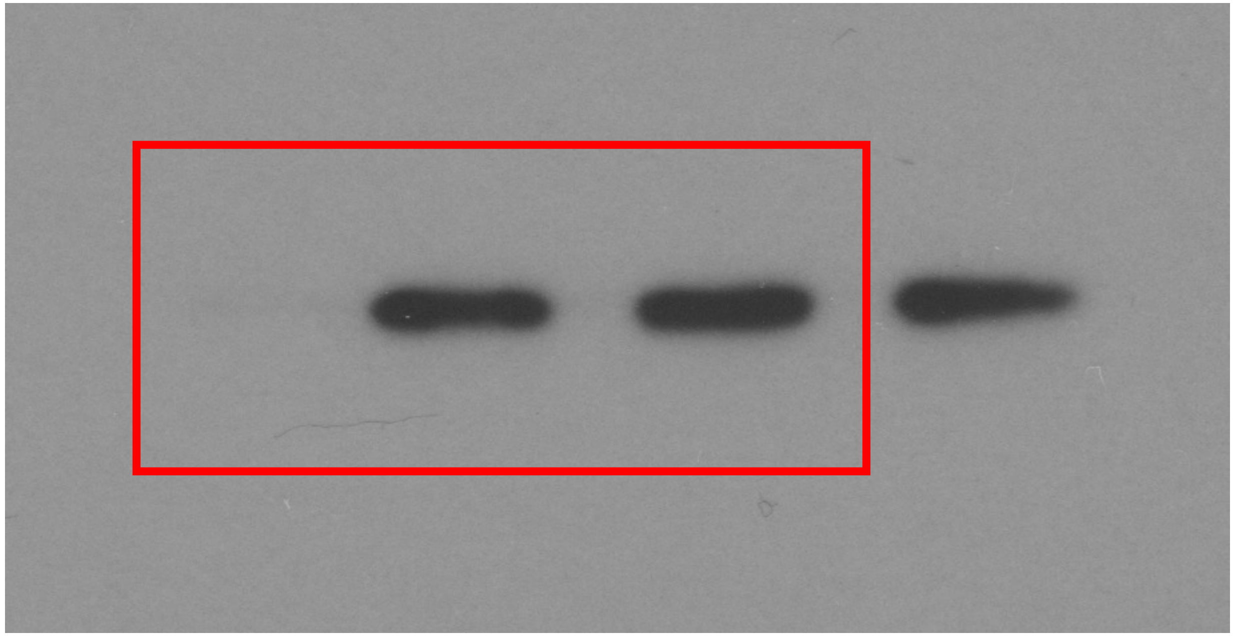

Fig. 5H; S6

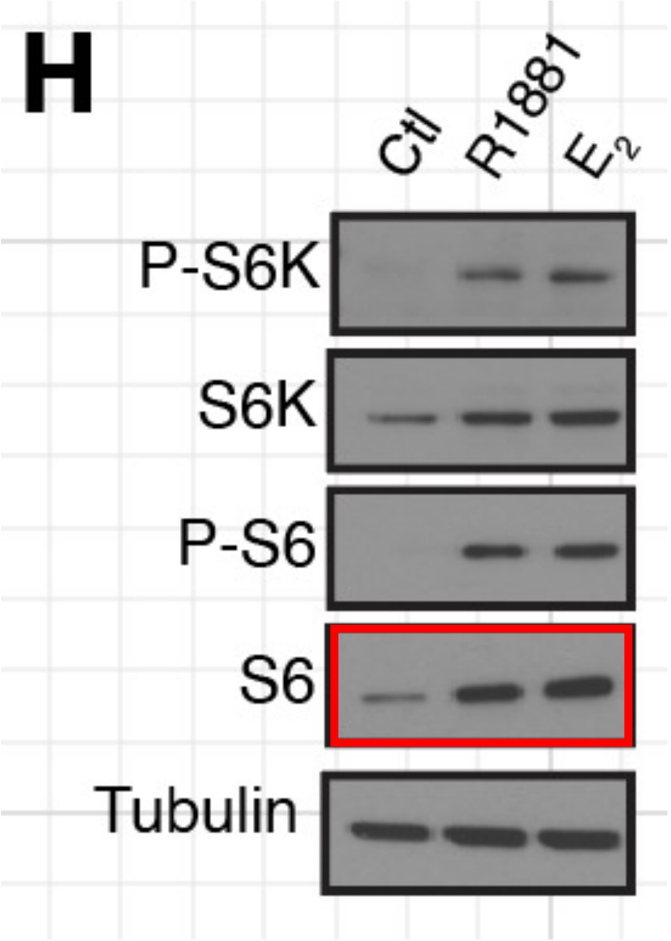

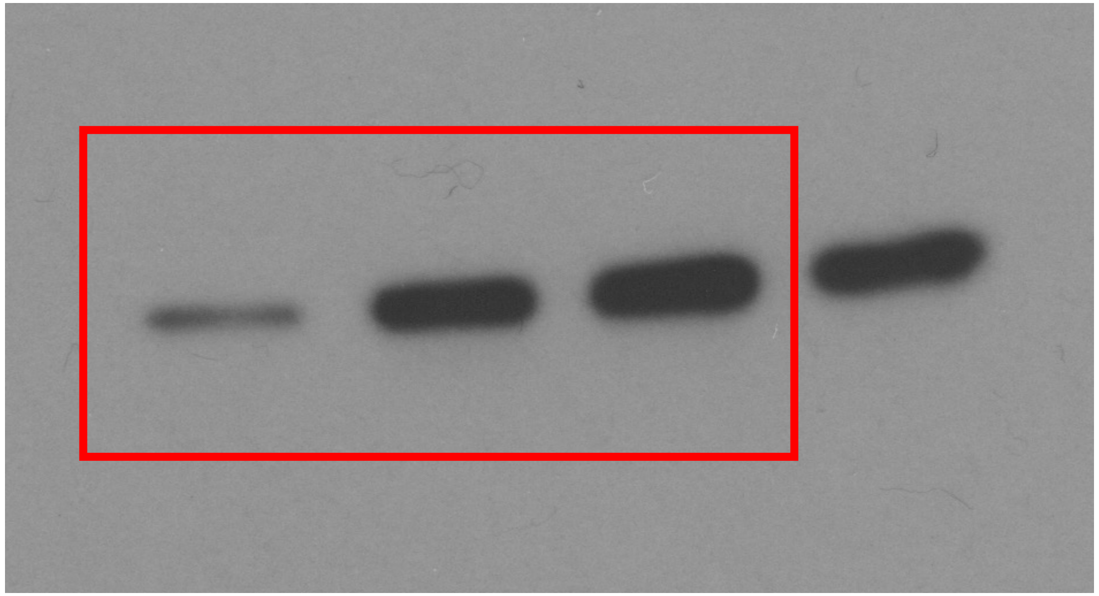

Fig. 5H; Tubulin

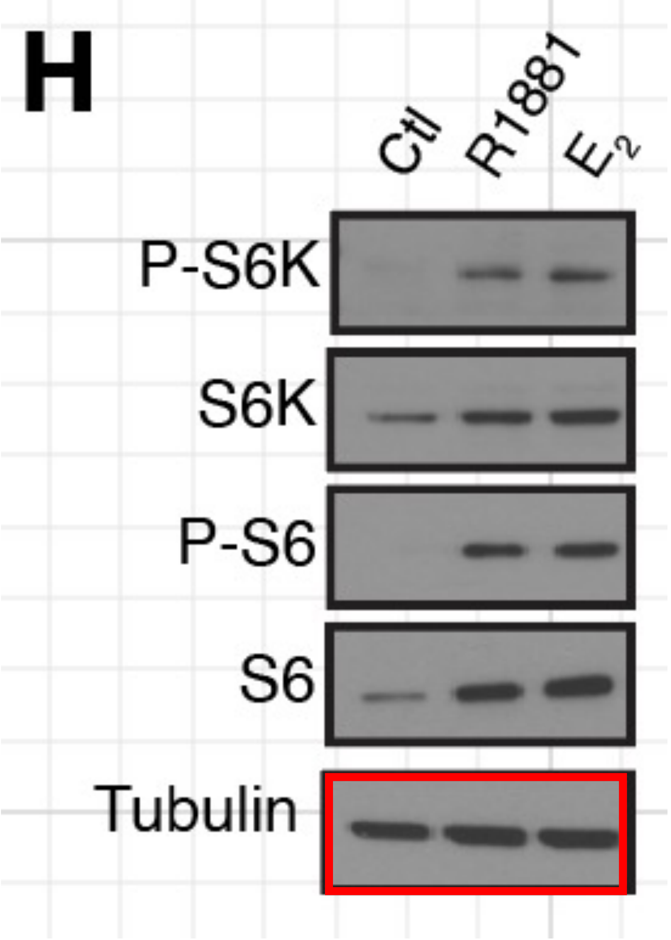

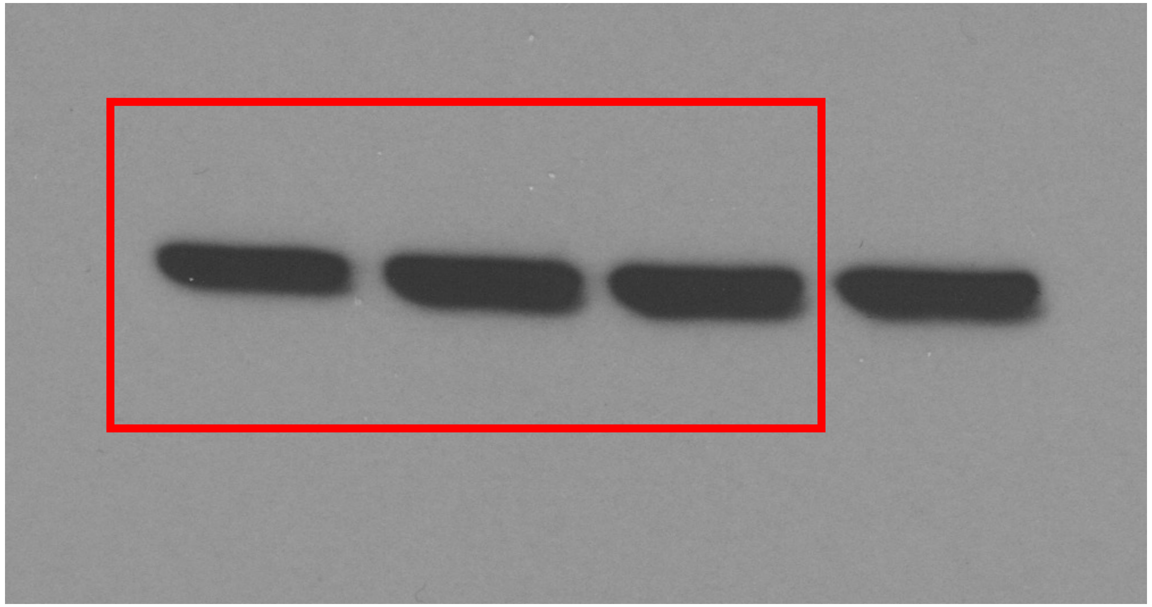

Supp. Fig. S5K; ERa short

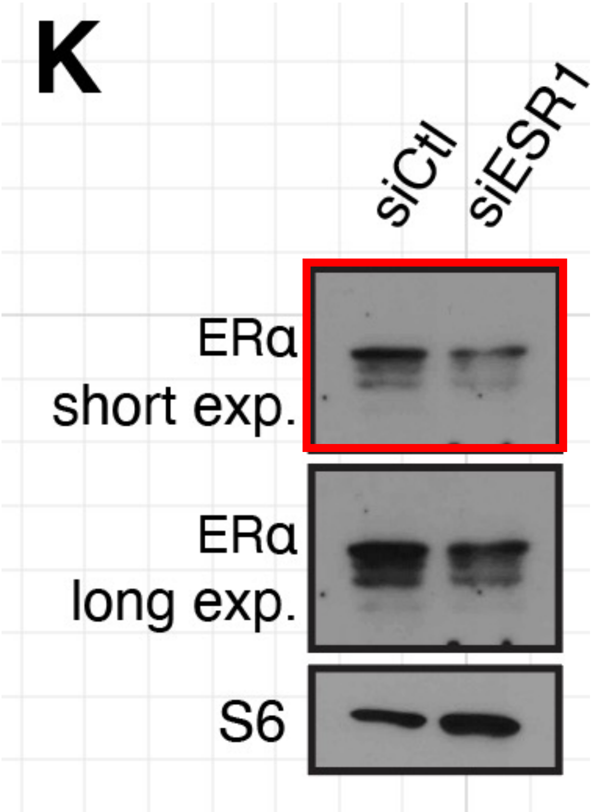

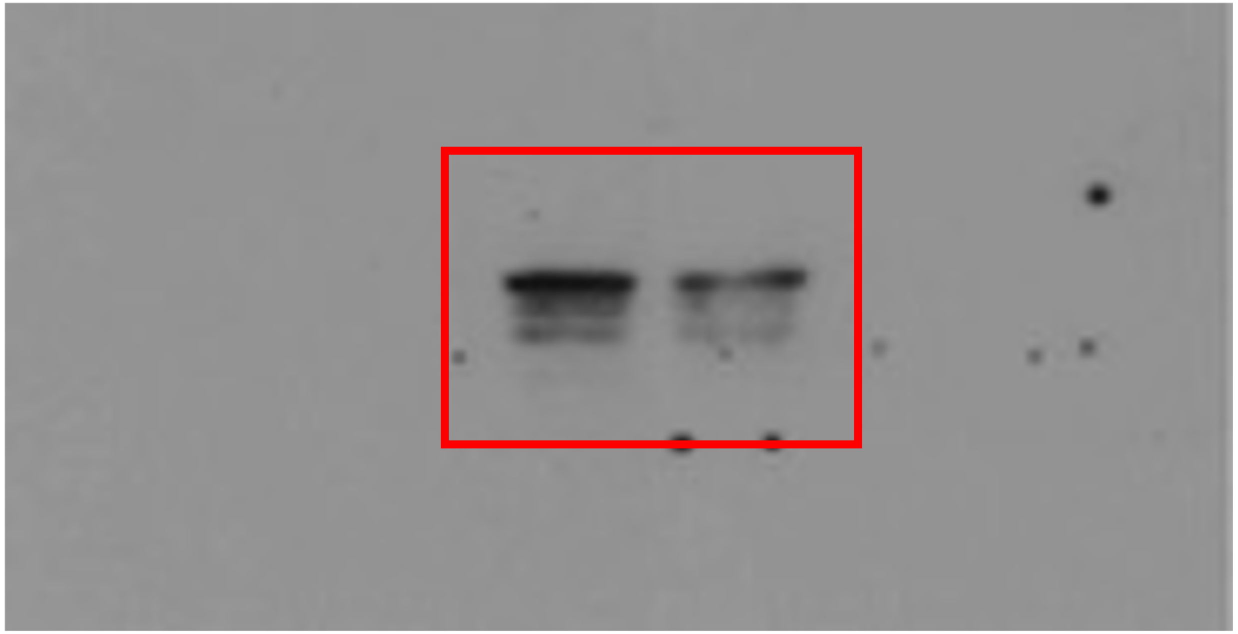

Supp. Fig. S5K; ERa long

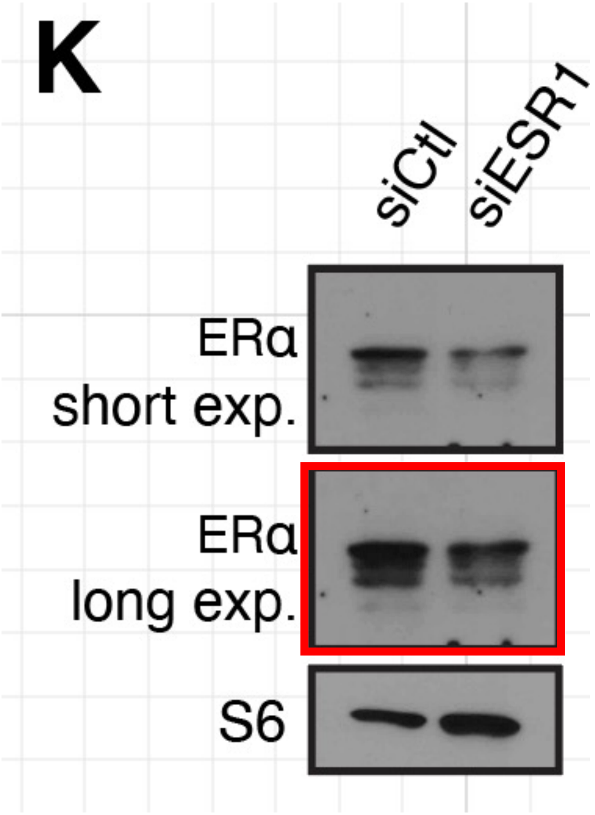

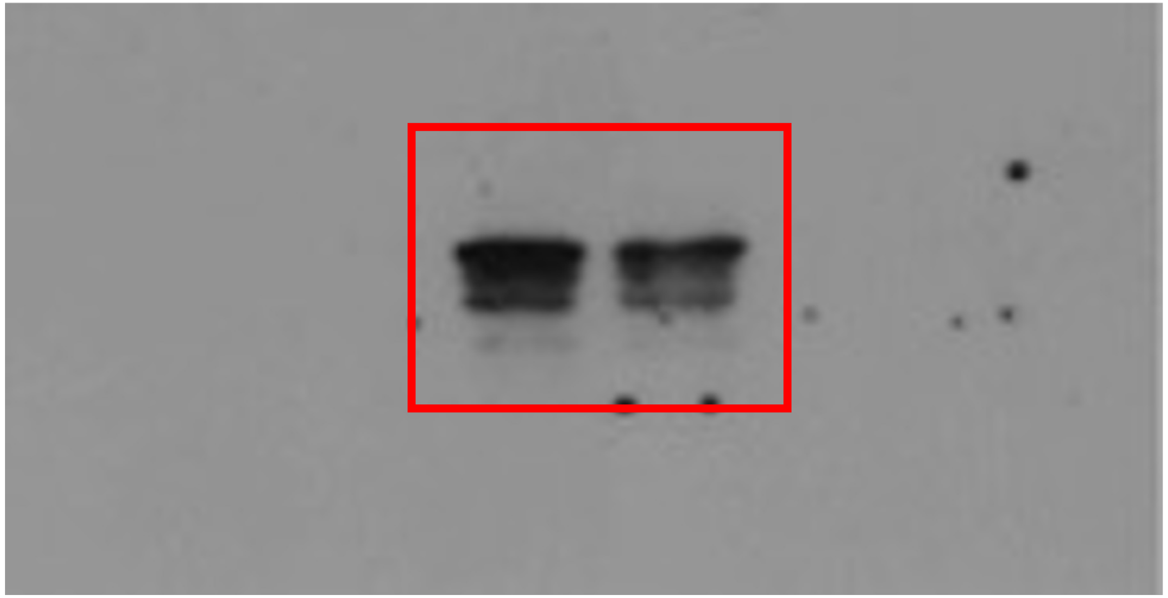

Supp. Fig. S5K; S6

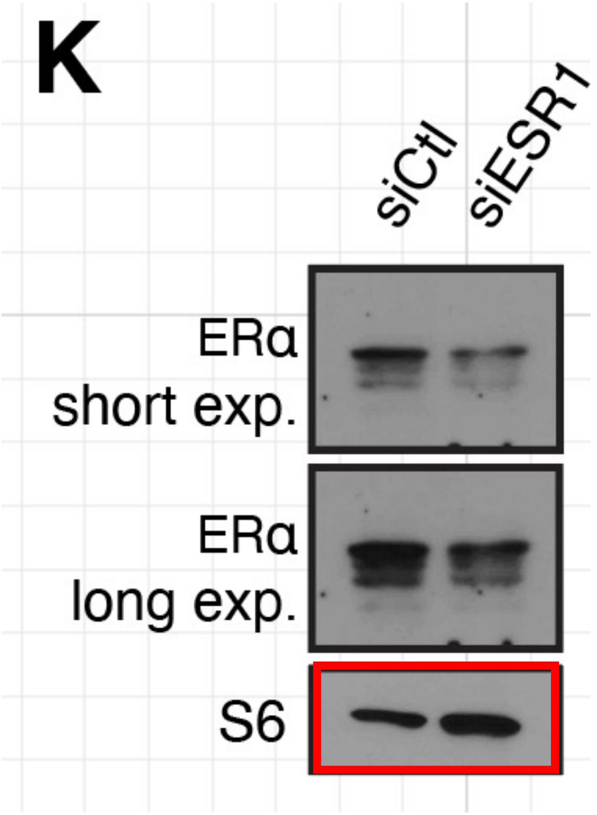

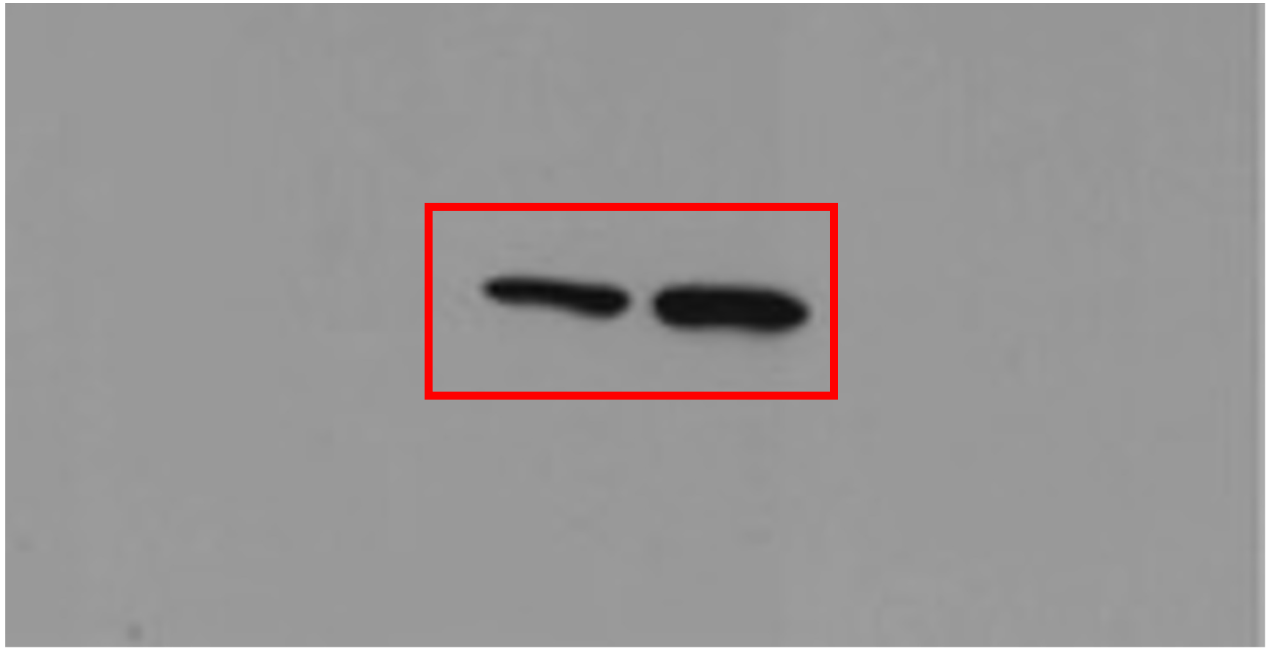

Supplement: Unedited blot and gel images [file jci-134-170809-s114.pdf]
